# Bio‐Based Surfactants via Borrowing Hydrogen Catalysis

**DOI:** 10.1002/chem.202500077

**Published:** 2025-02-13

**Authors:** Maximilian Koy, Maximilian Fellert, Chuting Deng, Michiel T. Uiterweerd, Alicia Lessentier, Minyan Wu, Mickael Cregut, Jianxia Zheng, Stephane Streiff, Juan J. de Pablo, Ben L. Feringa

**Affiliations:** ^1^ Stratingh Institute for Chemistry University of Groningen Nijenborgh 3 9747 AG Groningen, The Netherlands; ^2^ Pritzker School of Molecular Engineering University of Chicago 5640 South Ellis Avenue Chicago, Illinois 60637 United States of America; ^3^ SYENSQO/Solvay Research Innovation Center of Lyon Biomattech Platform 85 Avenue des Freres Perret 69320 Saint-Fons France; ^4^ SYENSQO Eco-Efficient Products and Processes Laboratory (E2P2L) 3966 Jindu Rd. Xinzhuang Industrial Zone Shanghai 201108 China

**Keywords:** Borrowing hydrogen, Surfactants, Green chemistry, Sustainability, Foaming agent

## Abstract

A borrowing hydrogen approach to produce bio‐based surfactants is described. The process utilizes ubiquitous amino acids and common alcohols without protecting group manipulations. Surfactants are synthesized in a single step using a commercially available ruthenium‐based catalyst in a waste‐free manner with nearly ideal atom economy. The versatility of the products is shown by further derivatization resulting in novel Gemini surfactants and a related quaternary ammonia salt. The analysis of selected compounds shows remarkable properties as surfactants. Further studies show their potential biodegradability in nature, which enhances the broad application profile of the sustainable products prepared in this study.

## Graphical abstract

A borrowing hydrogen approach to produce bio‐based surfactants is described. Using amino acids and common alcohols without protecting groups, surfactants are synthesized in one step with a ruthenium catalyst, achieving nearly ideal atom economy, including Gemini surfactants and a quaternary ammonia salt, with remarkable surfactant properties and potential biodegradability, enhancing the broad application profile of these sustainable products.

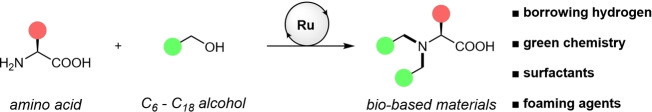

## Introduction

**Figure 1:**
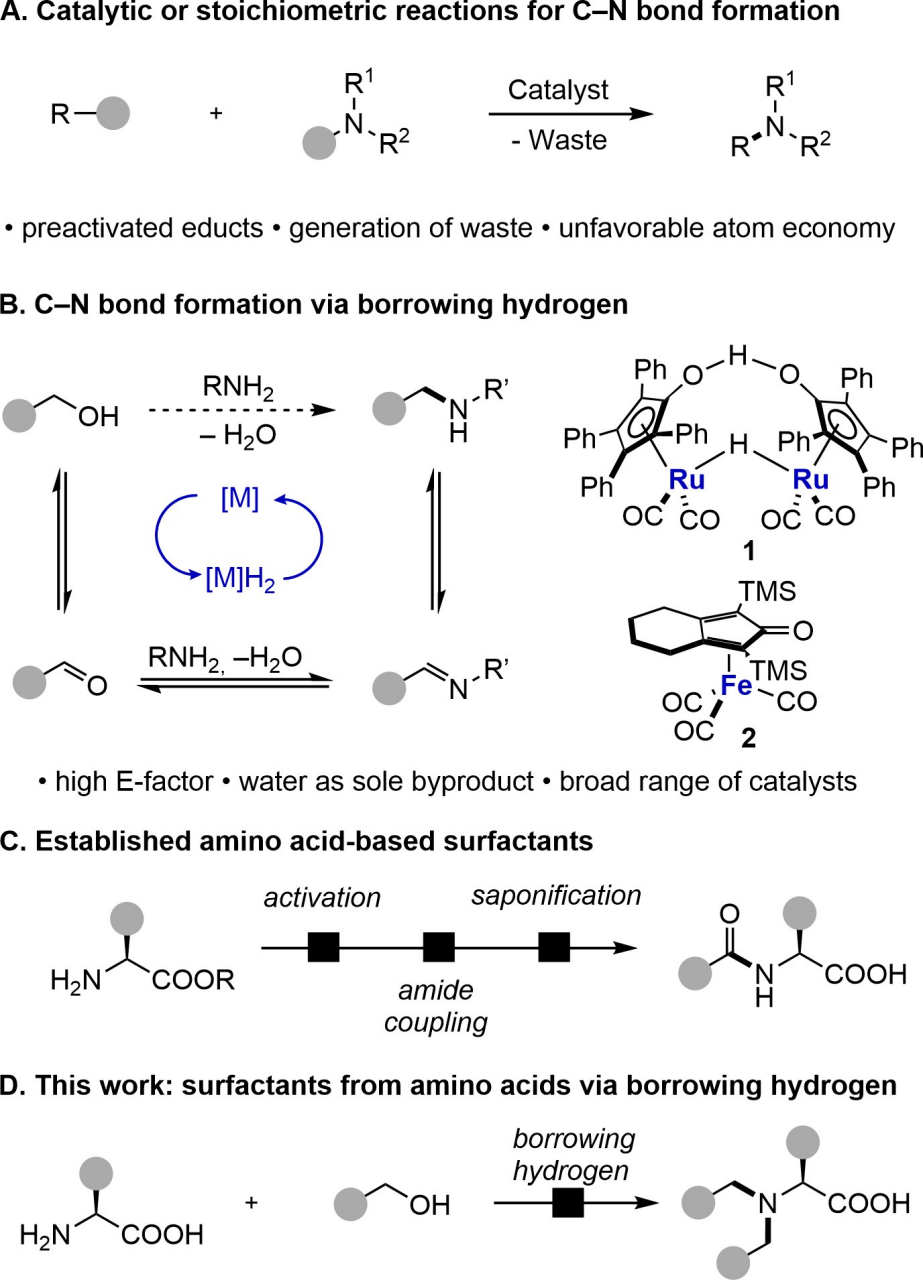
Overview of C−N bond formation procedures and context of this work. TMS=trimethylsilyl.

Modern organic synthesis needs to consider various aspects of green chemistry such as atom economy, step count, avoidance of waste and the use of bio‐based and renewable starting materials.[^1^, ^2^, ^3^] Within the field of green chemistry, atom economy and the E‐factor are important metrics to account for the preservation of reactants in the final product and reduced waste generation.[^4^, ^5^] In particular, the formation of carbon–nitrogen bonds – which plays a prominent role in active ingredients and advanced materials – is often plagued by unfavorable values for these sustainability parameters. Stoichiometric C−N bond formation reactions such as the Gabriel synthesis or reductive amination are typically hampered by activating groups with high molecular weight or the stoichiometric generation of salts as potentially toxic waste. But also, catalytic reactions including Buchwald‐Hartwig aminations or Ullmann couplings require prefunctionalized starting materials such as halides or triflates leading to unfavorable reaction step counts, low atom economy and generation of waste (see Figure 1A).[^6^, ^7^]

Catalytic borrowing hydrogen conversions allow oxidation of alcohols, in situ imine formation and reduction to the desired amine by a single catalyst in one pot with formation of water as the sole by‐product, resulting in almost ideal atom economy, E‐factor and reaction step count (see Figure 1B).[^8^, ^9^, ^10^] A wide range of transition metal catalysts based on ruthenium,[^11^, ^12^, ^13^, ^14^] iridium,[^15^, ^16^, ^17^, ^18^, ^19^] or first row transition metals such as iron,[^20^, ^21^, ^22^] manganese,[^23^, ^24^, ^25^, ^26^, ^27^] or cobalt[^28^, ^29^, ^30^] is available for this purpose.

Our group[^20^, ^21^, ^31^, ^32^] and Martin‐Matute and co‐workers^[33]^ recently reported the alkylation of natural amino acids with alcohols by Shvo's catalyst **1**, Knölker's catalyst **2**, and iridium‐based catalysts. This reaction offers a very attractive approach to a sustainable production of functional molecules such as surfactants due to favorable green chemistry metrics and the bioavailability of both amino acids and alcohols. In the literature, amino acids are typically used to prepare surfactants by introducing the lipophilic part at the *N*‐terminus of the amino acid as an amide.[^34^, ^35^] Normally, this reaction starts from long‐chain carboxylic acids, which react with amino acid esters to form the corresponding amide, followed by saponification to obtain the surfactant (see Figure 1C). Due to a three‐step synthesis, the use of amide coupling reagents or activation via acid chlorides, this results in unfavorable green chemistry metrics. In contrast, direct alkylation of amino acids with alcohols would yield only water as a by‐product in a one‐step protecting group‐free reaction (see Figure 1D). Moreover, further functionalization of surfactants prepared by amide coupling is limited to the acid function, whereas surfactants synthesized by borrowing hydrogen have two versatile functional groups, an amino group and an acid group, which allows further engineering of their structure and function. Overall, we here describe the establishment of a borrowing hydrogen‐based approach for the facile preparation of amino acid‐based surfactants and the characterization of their properties.

## Results and Discussion

**Figure 2:**
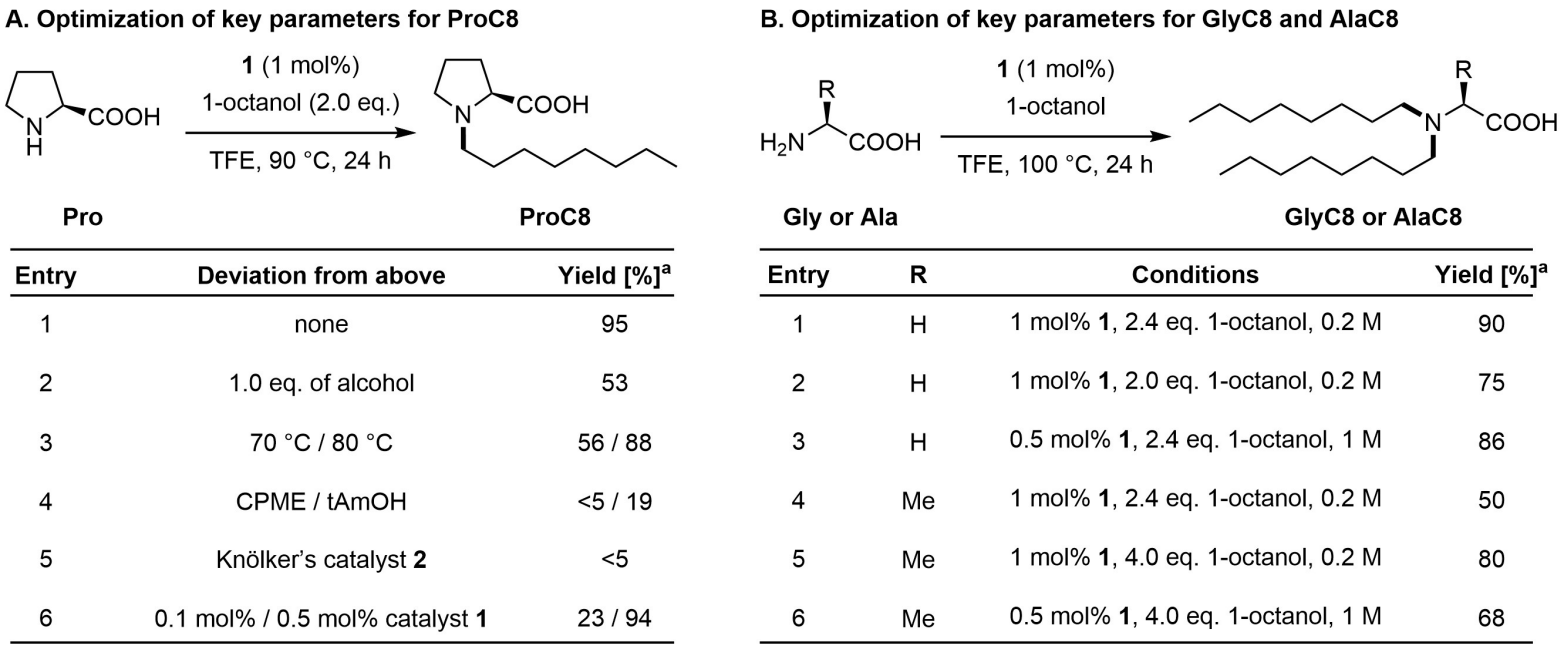
Optimization of key reaction parameters. [a] Determined by ^1^H NMR using CH_2_Br_2_ as internal standard.

Based on our previous experience, we started the optimization with proline and 1‐octanol using Shvo's catalyst **1**, since only mono‐alkylation is possible (Figure 2A).^[31]^ Under optimized conditions, product **ProC8** was obtained in 95 % yield using 1 mol % of Shvo's catalyst and a slight excess of alcohol in trifluoroethanol (TFE) at 90 °C. A lower yield of 53 % of **ProC8** was observed with equimolar stoichiometry between 1‐octanol and proline (Figure 2A, entry 2). Lower temperatures also reduce the yield of **ProC8** (Figure 2A, entry 3). In addition, cyclopentyl methyl ether (CPME) and tAmOH as greener and less toxic solvents were investigated, but this resulted in significantly lower yields (Figure 2A, entry 4). Knölker's catalyst **2** based on iron as an attractive first‐row transition metal was evaluated but yielded the product only in low yields (Figure 2A, entry 5). However, the amount of Shvo's catalyst can be decreased to 0.5 mol % with comparable yield of 94 % (Figure 2A, entry 6). Further reduction of the catalyst loading to 0.1 mol % results in insufficient yield of 23 %.

**Figure 3:**
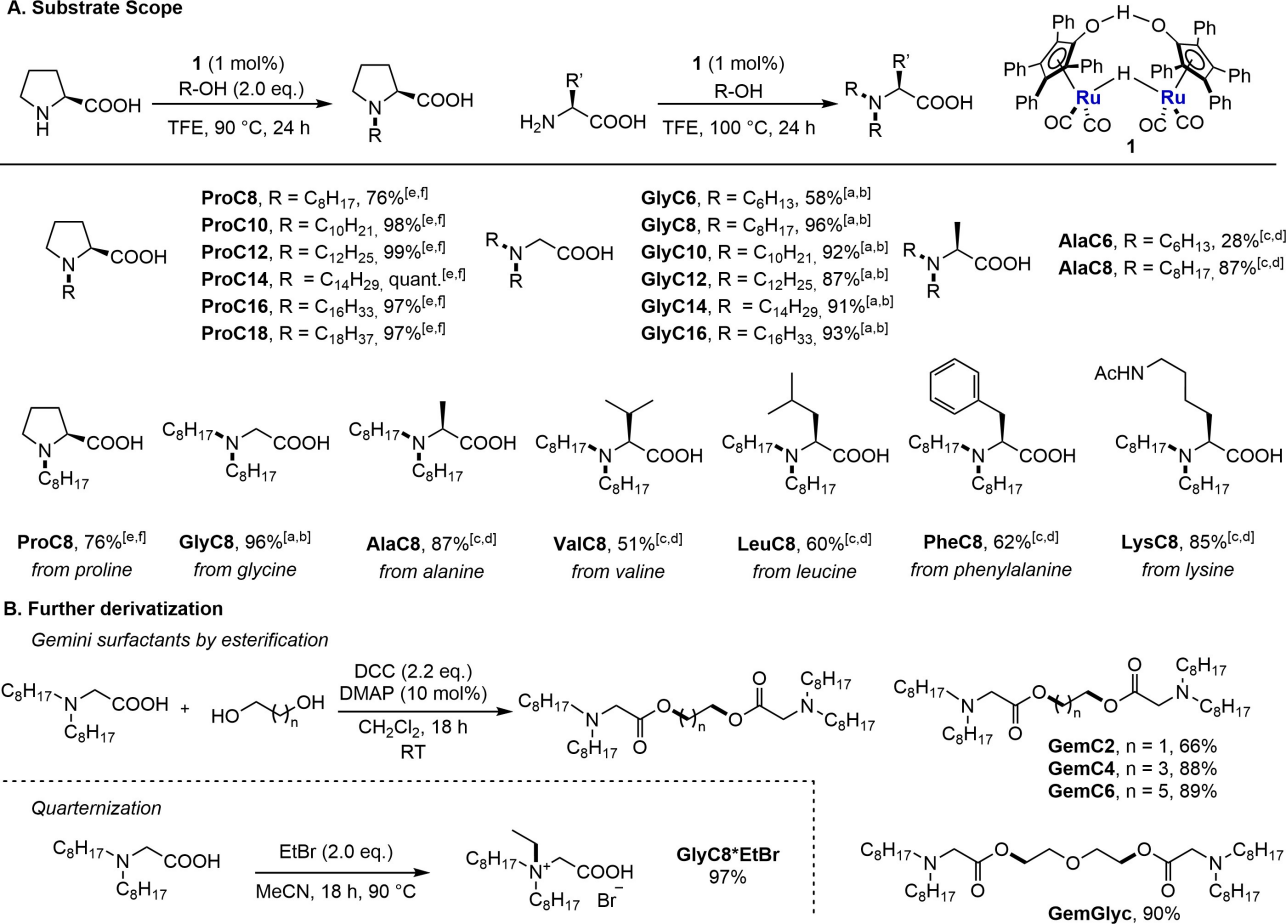
Substrate scope and further derivatization of the surfactants. [a] small scale: glycine (1.0 eq.), alcohol (2.4 eq.), **1** (1 mol %), TFE (0.2 M), 100 °C, 24 h. [b] large scale: glycine (1.0 eq.), alcohol (3.0 eq.), **1** (0.5 mol %), TFE (1.0 M), 100 °C, 24 h. [c] small scale: amino acid (1.0 eq.), alcohol (4.0 eq.), **1** (1 mol %), TFE (0.2 M), 100 °C, 24 h. [d] large scale: amino acid (1.0 eq.), alcohol (4.0 eq.), **1** (0.5 mol %), TFE (1.0 M), 100 °C, 24 h. [e] small scale: proline (1.0 eq.), alcohol (2.0 eq.), **1** (1 mol %), TFE (0.2 M), 90 °C, 24 h. [f] large scale: proline (1.0 eq.), alcohol (1.2 eq.), **1** (0.5 mol %), TFE (1.0 M), 90 °C, 24 h.

Next, the alkylation was expanded to include additional amino acids with primary amine groups. Initial experiments indicated clear selectivity for dialkylation, so more detailed optimization studies targeted dialkylation. Glycine was successfully reacted at 100 °C with only 2.4 eq. of 1‐octanol to give *N,N*‐dialkylated **GlyC8** in 90 % yield (Figure 2B, entry 1), while using 2.0 eq. resulted in a lower yield of 75 % (Figure 2B, entry 2). For glycine, the catalyst loading can as well be decreased to 0.5 mol % without observing a significant reduction in yield (Figure 2B, entry 3). On transition to alanine, **AlaC8** was formed in a reduced yield of 50 % using 2.4 eq. of 1‐octanol (Figure 2B, entry 4). Gratifyingly, by increasing the amount of 1‐octanol to 4.0 eq the bis‐alkylated product **AlaC8** was obtained in 80 % yield (Figure 2B, entry 5). A lower catalyst loading of 0.5 mol % is possible with a lower yield of 68 % (Figure 2B, entry 6). With the optimized conditions established, a range of amino acids and alcohols was screened for borrowing hydrogen reactions (Figure 3A). Proteinogenic amino acids were used in this study. Previously, we have shown that racemization only occurred to a small extent.^[31]^ First, various linear primary alcohols with different chain lengths were investigated with proline and glycine. Products derived from alcohols with distinct chain lengths from C8–C18 were isolated in excellent yields (87–quant. %). Varying the alcohol component ranging from C8–C18 allows the synthesis of tailor‐made functional materials, as this enables the properties of potential building blocks to be fine‐tuned. Subsequently, various amino acids were reacted with 1‐octanol. It was found that yields were lower with increased steric hinderance at the α‐position to the amino function. **AlaC8**, **ValC8**, **LeuC8**, and **PheC8** could nevertheless be obtained in good yields. In addition, the product from acetyl‐protected lysine was successfully isolated in very good and synthetically useful yield. The additional functional group in **LysC8** allows further manipulation of the compounds’ properties.

The products are characterized by versatile amino and acid functionalities, being handles that allow the properties of the surfactants to be adjusted. **GlyC8** was selected as a substrate for further functionalization with the aim of improving properties such as solubility (Figure 3B). First, **GlyC8** was reacted with various diols to form the corresponding Gemini surfactants by esterification. Gemini surfactants are characterized by higher solubility and often additional improved properties.^[36]^ Diols with different alkyl chain lengths could be used as linkers to obtain **GemC2**, **GemC4**, and **GemC6**, while diethylene glycol was used as a polar linker to prepare **GemGlyc** in the presence of *N,N’*‐dicyclohexylcarbodiimide (DCC) and 4‐dimethylaminopyridine (DMAP). Finally, functionalization was carried out on the amino moiety of **GlyC8** by alkylation with ethyl bromide to give product GlyC8*EtBr as quaternary ammonium salt in excellent yield.

A representative selection of the products was analyzed regarding their properties as surfactants. First, the solubility in aqueous solution was investigated. At a neutral pH=7 and an initial concentration of 1 wt %, only **ProC8** showed to be slightly water‐soluble. Adjustment of surfactant concentration and pH allowed solubilization of **GlyC6**, **AlaC6**, **AlaC8**, **LysC8**, **GemGlyc**, and GlyC8*EtBr. **GlyC6**, **AlaC6**, **AlaC8** and **LysC8** showed enhanced solubility in basic aqueous media (pH=9), while acidic media (pH=2) improved the solubility of **GemGlyc** and GlyC8*EtBr. Under basic conditions, **ProC8** could be solubilized up to a concentration of 10 wt %, surpassing the solubility of all other surfactants tested and commercially available surfactants such as cetyltrimethylammonium bromide (CTAB).^[37]^ **ProC8** is functionalized with only one alkyl chain compared to two alkyl chains in all other surfactants, increasing its polarity and leading to better solubility in water. The solubility of **GlyC8**–**GlyC14** and **PheC8** in water could not be improved regardless of pH level (pH=2–9) or temperature (25 °C–80 °C). In contrast, their monoalkylated analogues have been applied as surfactants in aqueous solution.[^38^, ^39^] To explain the differences in solubility of **GlyC8** and **AlaC8**, we used an estimation of the solvation free energy and the potential of mean force based on density functional theory calculations and molecular dynamics simulations. Overall, these simulation results suggest that the solubility trend observed experimentally is likely attributed to multiple complicated factors during the aggregation process instead of the properties of individual molecules (for details, see SI, section 5).

**Figure 4:**
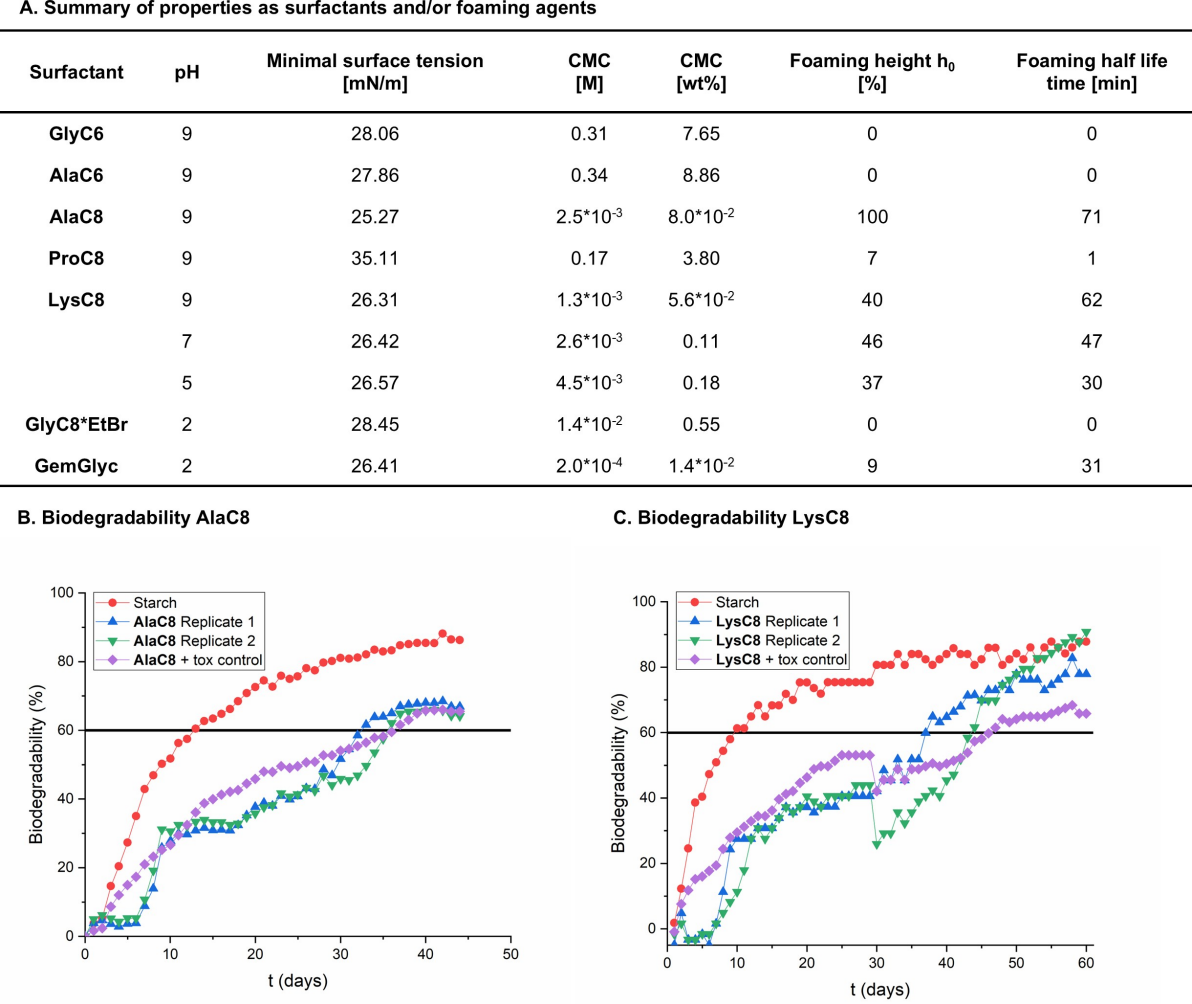
Properties of selected surfactants. A. Minimal surface tension, CMC and foaming properties. B. Biodegradability of **AlaC8**. C. Biodegradability of **LysC8**.

Subsequent characterizations focused exclusively on soluble surfactants **GlyC6**, **AlaC6**, **AlaC8**, **ProC8**, **LysC8**, GlyC8*EtBr and **GemGlyc**. Minimal surface tension values γ_cmc_ and critical micelle concentration (CMC) were determined by pending drop surface tensiometry, observing values between 25 and 35 mN m^−1^ (for details, see Figure 4A and SI, section 2). Notably, **AlaC8** and **LysC8** displayed competitive surface tensions, comparable to the ones of common polyfluorinated, silicone or sulfonic acid‐based surfactants^[40]^ without displaying the disadvantages of these surfactant classes such as limited biodegradability and instability in acidic and basic media. CMC values range from 0.002–8.86 wt % or 0.2–340 mM, respectively. Remarkably, **LysC8** displayed a low CMC over a broad pH range (pH=5–9), comparable to the CMC of the widely used surfactant CTAB (1 mM).^[41]^ **GemGlyc** showed an even lower CMC, but limited solubility and homogeneity of its solutions might impede some specific applications.

Additionally, the foaming ability was tested in terms of maximal foaming height h_max_ and foam stability as half‐life time t_1/2_. Surfactants **GlyC6**, **AlaC6**, **ProC8**, GlyC8*EtBr and **GemGlyc** showed no significant foaming, while **AlaC8** exhibited very good foaming ability (h_max_=100 %) and a half‐life time of t_1/2_=71 min. **LysC8** exhibited good foaming ability (37–46 %) and long half‐life times (t_1/2_=30–62 min) over a broad pH level ranging from pH=5–9.

Finally, the biodegradability of the most promising surfactants, **AlaC8** and **LysC8**, was assessed (Figure 4B, for details, see SI, section 4). Comparison against the theoretical oxygen demand (ThOD) shows that two replicates of **AlaC8** pass a threshold of 60 % after day 35 and 37, respectively, while the replicates of **LysC8** pass this threshold after 37 and 44 days, respectively. Starch as a positive internal reference confirms both the validity and the non‐toxicity of the assay. Therefore, we identified **AlaC8** and **LysC8** to be considered as inherently biodegradable and non‐toxic surfactants according to the OECD 301F protocol.^[42]^

## Conclusions

In conclusion, we have synthesized various bio‐based surfactants derived from amino acids in high atom and step economy using borrowing hydrogen catalysis with a commercially available ruthenium‐based catalyst. Versatility of the products has been shown by further derivatization to Gemini surfactants and quaternary ammonium salts. Surfactant properties, foaming ability, biodegradability and toxicity tests were conducted for selected surfactants, which led to the discovery of highly promising bio‐based surfactant candidates **AlaC8** and **LysC8**. We envision that this study will provide a basis for sustainable access to bio‐based chemicals and will stimulate further development using alternative first‐row transition metal catalysts and milder reaction conditions.

## Conflict of Interests

The authors declare no conflict of interest.

1

## Supporting information

As a service to our authors and readers, this journal provides supporting information supplied by the authors. Such materials are peer reviewed and may be re‐organized for online delivery, but are not copy‐edited or typeset. Technical support issues arising from supporting information (other than missing files) should be addressed to the authors.

Supporting Information

## Data Availability

The data that support the findings of this study are available in the supplementary material of this article.
