# Supplementary material for: Bio‐Based Surfactants via Borrowing Hydrogen Catalysis
Source: Chemistry. 2025 Feb 13;31(17):e202500077. doi: 10.1002/chem.202500077 (PMC11924988; doi:10.1002/chem.202500077)
Supplement: Supplementary file 1 — Supporting Information [file CHEM-31-e202500077-s001.pdf]

Chemistry–A European Journal

Supporting Information

Bio-Based Surfactants via Borrowing Hydrogen Catalysis

Maximilian Koy, Maximilian Fellert, Chuting Deng, Michiel T. Uiterweerd, Alicia Lessentier, Minyan Wu, Mickael Cregut, Jianxia Zheng, Stephane Streiff, Juan J. de Pablo, and Ben L. Feringa*

Supporting Information

Table of contents

1. Synthetic Experimental Procedures S2
   - 1.1 General Procedures S2
   - 1.2 Optimization of Reaction Conditions S3
   - 1.3 General Procedures for Alkylation of Amino Acids and Synthesis of Gemini Surfactants S7
   - 1.4 Scope and Characterization S9
2. CMC Determination S15
3. Foaming Analysis S20
4. Biodegradability Assay S21
5. Computational Studies S24
6. NMR Spectra of New Compounds S29
7. References S73

1. Synthetic Experimental Procedures

1.1 General Procedures

Commercial reagents and solvents:
All chemicals and solvents were purchased from commercial suppliers unless otherwise stated. All amino acids have been used in their enantiopure L-form.

Synthesis and purification:
Standard Schlenk techniques were used, employing nitrogen or argon as the inert gas. If they were not performed at room temperature, the reaction temperatures refer to the temperature of the heating/cooling bath or heating block.
Flash column chromatography was performed on a Biotage Select system using the indicated solvents. TLC analysis was done on Merck silica gel 60 F_{254} aluminum sheets, and compounds were visualized with a UV lamp or by staining with a $KMNO_4$ solution (254 nm or 365 nm).

NMR:
Full characterization of the newly synthesized compounds (including ^{1}H, ^{13}C, and 2D NMR experiments) was performed using a Mercury-Plus 400 (400 MHz) or Bruker Avance Neo 600 (600 MHz) spectrometer. Chemical shifts (δ) are given in parts per million (ppm) relative to TMS, using the solvent residual peak as internal standard ($CDCl_3$: δ = 7.26 for ^{1}H, δ = 77.16 for ^{13}C). Data is reported as follows: chemical shifts (δ) in ppm, multiplicity (s = singlet, bs = broad singlet, d = doublet, dd = doublet of doublets, t = triplet, q = quartet, m = multiplet), coupling constants *J* (Hz), and integration.

High-resolution mass:
HMRS spectra were recorded on a Thermofisher LTQ Orbitrap XL.

1.2 Optimization of Reaction Conditions

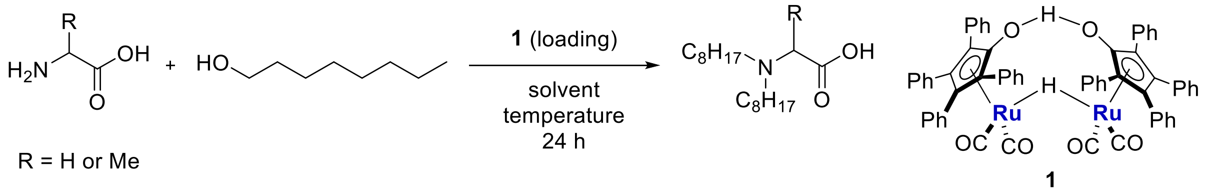

Figure S1: General equation for optimization of reaction conditions summarized in Table S1-S6 for the alkylation of glycine or alanine.

Table S1: Optimization of equivalents of alcohol for the dialkylation of glycine.[a]

Entry	Equivalents of 1-octanol	Yield[b] [%]
1	2.0	52
2	2.4	75
3	3.0	86
4	4.0	94

[a]Glycine (0.4 mmol), 1-octanol, Shvo's catalyst (1 mol%), CF_3CH_2OH (0.2 M), 90 °C, 24 h.
[b]Determined by ^1H NMR spectroscopy using CH_2Br_2 as internal standard.

TableS2: Optimization of concentration for the dialkylation of glycine.[a]

Entry	Concentration [M]	Yield[b] [%]
1	0.1	91
2	0.2	94
3	0.4	91

[a]Glycine (0.4 mmol), 1-octanol (4.0 eq.), Shvo's catalyst (1 mol%), CF_3CH_2OH (0.2 M), 90 °C, 24 h.
[b]Determined by ^1H NMR spectroscopy using CH_2Br_2 as internal standard.

Table S3: Optimization of temperature for the dialkylation of glycine.[a]

Entry	Temperature [°C]	Yield[b] [%]
1	70	91
2	80	94
3	90	91
4	100	91

[a]Glycine (0.4 mmol), 1-octanol (4.0 eq.), Shvo's catalyst (1 mol%), CF_3CH_2OH (0.2 M), 24 h.
[b]Determined by ^1H NMR spectroscopy using CH_2Br_2 as internal standard.

Table S4: Reoptimization of stoichiometry at 100 °C for the dialkylation of glycine.[a]

Entry	Equivalents of 1-octanol	Yield[b] [%]
1	2.0	75
2	2.4	90
3	3.0	91
4	4.0	91

[a]Glycine (0.4 mmol), 1-octanol, Shvo's catalyst (1 mol%), CF_3CH_2OH (0.2 M), 100 °C, 24 h.
[b]Determined by ^1H NMR spectroscopy using CH_2Br_2 as internal standard.

Table S5: Parameters for scale-up for the dialkylation of glycine.[a]

Entry	Conditions	Yield[b] [%]
1	1.0 mol% catalyst, 2.4 eq. 1-octanol	87
2	0.5 mol% catalyst, 2.4 eq. 1-octanol	86
3	0.5 mol% catalyst, 3.0 eq. 1-octanol	95

[a]Glycine (4.0 mmol), 1-octanol, Shvo's catalyst, CF_3CH_2OH (1.0 M), 100 °C, 24 h.
[b]Determined by ^1H NMR spectroscopy using CH_2Br_2 as internal standard.

Table S6: Re-optimization for alanine.[a]

Entry	Conditions	Yield[c] [%]
1[a]	2.4 eq. 1-octanol, 100 °C	50
2[a]	2.4 eq. 1-octanol, 110 °C	63
3[a]	3.0 eq. 1-octanol, 100 °C	71
4[a]	3.0 eq. 1-octanol, 110 °C	68
5[a]	4.0 eq. 1-octanol, 100 °C	80
6[a]	5.0 eq. 1-octanol, 100 °C	81
7[a]	6.0 eq. 1-octanol, 100 °C	84
8[b]	4.0 eq. 1-octanol, 100 °C, 1.0 M, 1.0 mol% catalyst	70
9[b]	4.0 eq. 1-octanol, 100 °C, 1.0 M, 0.5 mol% catalyst	68

[a]Alanine (0.4 mmol), 1-octanol, Shvo's catalyst, CF_3CH_2OH (0.2 M), 24 h.
[b]Alanine (4.0 mmol), 1-octanol, Shvo's catalyst, CF_3CH_2OH (1.0 M), 100 °C, 24 h.
[c]Determined by ^1H NMR spectroscopy using CH_2Br_2 as internal standard.

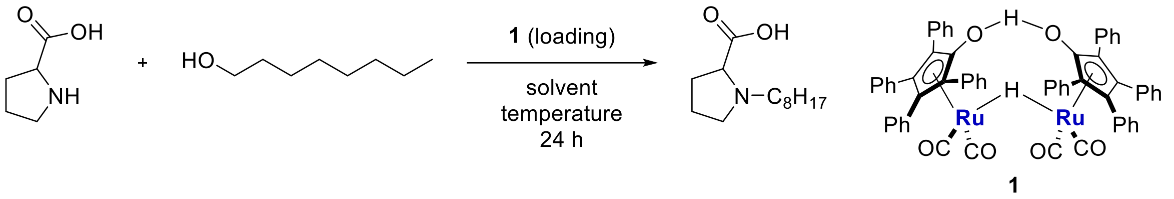

Figure S2: General equation for optimization of reaction conditions summarized in Table S7-S11 for the alkylation of proline.

Table S7: Optimization of equivalents of alcohol.

Entry	Equivalents of 1-octanol	Yield[b] [%]
1	1	53
2	1.2	93
3	2	95
4	3	94
5	4	94

[a]Conditions: proline (0.4 mmol), 1-octanol, Shvo's catalyst (1 mol%), CF_3CH_2OH (0.2 M), 90 °C, 24 h.
[b]Determined by ^1H NMR spectroscopy using CH_2Br_2 as internal standard.

Table S8: Optimization of the concentration and tolerance of water.

Entry	Concentration [M]	Yield[b] [%]
1	0.05	97
2	0.2	98
3	0.4	97
4	0.2 (5% H_2O)	96
5	0.2 (10% H_2O)	85
6	0.2 (50% H_2O)	n.d.

[a]Conditions: proline (0.4 mmol), 1-octanol (2.0 eq.), Shvo's catalyst (1 mol%), CF_3CH_2OH (and water), 90 °C, 24 h.
[b]Determined by ^1H NMR spectroscopy using CH_2Br_2 as internal standard.

Table S9: Optimization of the temperature.

Entry	Temperature [°C]	Yield[b] [%]
1	50	<5
2	60	<5
3	70	56
4	80	88
5	90	98

[a]Conditions: proline (0.4 mmol), 1-octanol, Shvo's catalyst (1 mol%), CF_3CH_2OH (0.2 M), temperature, 24 h.
[b]Determined by ^1H NMR spectroscopy using CH_2Br_2 as internal standard.

Table S10: Miscellaneous parameters.

Entry	Variation	Yield[b] [%]
1	*tert*-amyl alcohol, 90 °C	19
2	*tert*-amyl alcohol, 120 °C	<5
3	PhMe, 120 °C	<5
4	Cyclopentyl methyl ether, 120 °C	<5
5	CF_3CH_2OH, 90 °C, under air	40

[a]Conditions: proline (0.4 mmol), 1-octanol, Shvo's catalyst (1 mol%), Solvent (0.2 M), temperature, 24 h.
[b]Determined by ^1H NMR spectroscopy using CH_2Br_2 as internal standard.

Table S11: Parameters for scale-up.

Entry	Conditions	Yield[b] [%]
1	0.5 mol% catalyst	94
2	0.1 mol% catalyst	23
3	0.1 mol% catalyst, 48 h reaction time	19
4	0.1 mol% catalyst + molecular sieves	23
5	1-octanol as limiting reagent, 1.2 eq. of proline, 0.5 mol% cat	58

[a]Conditions: proline (0.4 mmol), 1-octanol (1.2 eq.), Shvo's catalyst, CF_3CH_2OH (1 M), 90 °C, 24 h.
[b]Determined by ^1H NMR spectroscopy using CH_2Br_2 as internal standard.

1.3 General Procedures for Alkylation of Amino Acids and Synthesis of Gemini Surfactants

General procedure for alkylation of glycine (GP1, *small scale*):
Shvo's catalyst (1.0 mol%), the respective alcohol (if solid, 2.4 eq.) and glycine (1.0 eq.) were added to an oven dried microwave vial equipped with a magnetic stir bar. The microwave vial was sealed and evacuated/backfilled with N_2 (3x). Trifluoroethanol (Fluorochem, not dried, 0.2 M) and the respective alcohol (if liquid, 2.4 eq.) were added. For highly viscous alcohols, a stock solution in trifluoroethanol was prepared and subsequently transferred to the reaction vial. The reaction mixture was stirred at 100 °C for 24 h. Volatile compounds were removed under reduced pressure and the crude product was further purified by silica gel column chromatography.

General procedure for alkylation of glycine (GP2, *large scale*):
Shvo's catalyst (0.5 mol%), the respective alcohol (if solid, 3.0 eq.) and glycine (1.0 eq.) were added to an oven dried microwave vial equipped with a magnetic stir bar. The microwave vial was sealed and evacuated/backfilled with N_2 (3x). Trifluoroethanol (Fluorochem, not dried, 1.0 M) and the respective alcohol (if liquid, 3.0 eq.) were added. For highly viscous alcohols, a stock solution in trifluoroethanol was prepared and subsequently transferred to the reaction vial. The reaction mixture was stirred at 100 °C for 24 h. Volatile compounds were removed under reduced pressure and the crude product was further purified by silica gel column chromatography.

General procedure for alkylation of proline (GP3, *small scale*):
Shvo's catalyst (1.0 mol%), the respective alcohol (if solid, 2.0 eq.) and the respective amino acid (1.0 eq.) were added to an oven dried microwave vial equipped with a magnetic stir bar. The microwave vial was sealed and evacuated/backfilled with N_2 (3x). Trifluoroethanol (Fluorochem, not dried, 0.2 M) and the respective alcohol (if liquid, 2.0 eq.) were added. For highly viscous alcohols, a stock solution in trifluoroethanol was prepared and subsequently transferred to the reaction vial. The reaction mixture was stirred at 90 °C for 24 h. Volatile compounds were removed under reduced pressure and the crude product was further purified by silica gel column chromatography.

General procedure for alkylation of proline (GP4, *large scale*):
Shvo's catalyst (0.5 mol%), the respective alcohol (if solid, 1.2 eq.) and the respective amino acid (1.0 eq.) were added to an oven dried microwave vial equipped with a magnetic stir bar. The microwave vial was sealed and evacuated/backfilled with N_2 (3x). Trifluoroethanol (Fluorochem, not dried, 1.0 M) and the respective alcohol (if liquid, 1.2 eq.) were added. For highly viscous alcohols, a stock solution in trifluoroethanol was prepared and subsequently transferred to the reaction vial. The reaction mixture was stirred at 90 °C for 24 h. Volatile compounds were removed under reduced pressure and the crude product was further purified by silica gel column chromatography.

General procedure for alkylation of amino acids other than glycine and proline (GP5, *small scale*):
Shvo's catalyst (1.0 mol%), the respective alcohol (if solid, 4.0 eq.) and the respective amino acid (1.0 eq.) were added to an oven dried microwave vial equipped with a magnetic stir bar. The microwave vial was sealed and evacuated/backfilled with N_2 (3x). Trifluoroethanol (Fluorochem, not dried, 0.2 M) and the respective alcohol (if liquid, 4.0 eq.) were added. For highly viscous alcohols, a stock solution in trifluoroethanol was prepared and subsequently transferred to the reaction vial. The reaction mixture was stirred at 100 °C for 24 h. Volatile compounds were removed under reduced pressure and the crude product was further purified by silica gel column chromatography.

General procedure for alkylation of amino acids other than glycine and proline (GP6, *large scale*):

Shvo's catalyst (0.5 mol%), the respective alcohol (if solid, 4.0 eq.) and the respective amino acid (1.0 eq.) were added to an oven dried microwave vial equipped with a magnetic stir bar. The microwave vial was sealed and evacuated/backfilled with N_2 (3x). Trifluoroethanol (Fluorochem, not dried, 0.2 M) and the respective alcohol (if liquid, 4.0 eq.) were added. For highly viscous alcohols, a stock solution in trifluoroethanol was prepared and subsequently transferred to the reaction vial. The reaction mixture was stirred at 100 °C for 24 h. Volatile compounds were removed under reduced pressure and the crude product was further purified by silica gel column chromatography.

General procedure for esterification of alkylated amino acids (GP7):

The respective alkylated amino acid (2.4 eq.), DMAP (0.1 eq.), DCC (2.2 eq.) and the diol (1.0 eq., if solid), were added to a vial under air.[43] CH_2Cl_2 (0.25 M, not dry) and the diol (1.0 eq., if liquid) were added under air. The reaction mixture was allowed to stir at room temperature for 18 h. Volatile compounds were removed under reduced pressure, the crude mixture was dissolved in EtOAc and filtered over a short plug of celite. The crude reaction mixture was concentrated and further purified by silica gel column chromatography.

1.4 Scope and Characterization

GlyC6

The title compound was prepared according to GP1 on a 10.7 mmol scale and was obtained after silica gel chromatography (CH_2Cl_2:MeOH 99:1 -> 9:1) as an off-white solid (1.51 g, 6.21 mmol, 58%).

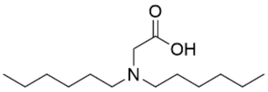

^{1}**H NMR** (400 MHz, $CDCl_3$) δ = 6.82 (bs, 1H), 3.47 (s, 2H), 3.09 – 3.03 (m, 4H), 1.72 – 1.64 (m, 4H), 1.36 – 1.23 (m, 12H), 0.87 (t, *J* = 7.1 Hz, 6H).
^{13}**C NMR** (151 MHz, $CDCl_3$) δ = 167.4, 56.7, 54.5, 31.4, 26.5, 24.0, 22.6, 14.0.
HRMS (ESI) *m/z* calc. for $C_{14}H_{29}NO_2+H^+$ 244.2271 [*M*+H]$^+$; found 244.2268.

GlyC8

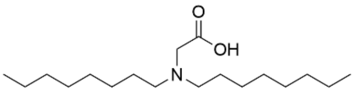

The title compound was prepared according to GP1 on a 0.400 mmol scale and was obtained after silica gel chromatography (CH_2Cl_2:MeOH 1:0 -> 5:1) as an off-white solid (115 mg, 0.383 mmol, 96%). The corresponding large-scale reaction was conducted according to GP2 on a 15.0 mmol scale (product: 3.77 g, 12.6 mmol, 84%).
^{1}**H NMR** (400 MHz, $CDCl_3$) δ = 8.32 (bs, 1H), 3.48 (s, 2H), 3.10 – 3.02 (m, 4H), 1.73 – 1.63 (m, 4H), 1.35 – 1.15 (m, 20H), 0.85 (t, J = 6.7 Hz, 6H).
^{13}**C NMR** (101 MHz, $CDCl_3$) δ = 167.7, 56.1, 54.0, 31.7, 29.1, 29.1, 26.8, 23.8, 22.6, 14.0.
HRMS (ESI) *m/z* calc. for $C_{18}H_{37}NO_2+H^+$: 300.2897 [*M*+H]$^+$; found 300.2893.

GlyC10

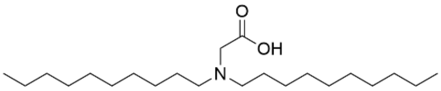

The title compound was prepared according to GP1 on a 0.400 mmol scale and was obtained after silica gel chromatography (CH_2Cl_2:MeOH 1:0 -> 10:1) as an off-white solid (130 mg, 0.366 mmol, 92%). The corresponding large-scale reaction was conducted according to GP2 on a 30.0 mmol scale (product: 9.29 g, 26.1 mmol, 87%).
^{1}**H NMR** (400 MHz, $CDCl_3$) δ = 6.82 (bs, 1H), 3.48 (s, 2H), 3.11 – 3.02 (m, 4H), 1.74 – 1.61 (m, 4H), 1.35 – 1.18 (m, 28H), 0.86 (t, *J* = 6.9 Hz, 6H).
^{13}**C NMR** (101 MHz, $CDCl_3$) δ = 167.5, 56.7, 54.4, 32.0, 29.6, 29.4, 29.3, 26.9, 24.0, 22.8, 14.2. (*Note: One ^{13}C signal is overlapping with another ^{13}C signal and cannot be distinguished.)*
HRMS (ESI) *m/z* calc. for $C_{22}H_{45}NO_2+H^+$ 356.3523 [*M*+H]$^+$; found 356.3531.

GlyC12

The title compound was prepared according to GP1 on a 0.4 mmol scale and was obtained after silica gel chromatography (CH_2Cl_2:MeOH 1:0 -> 10:1) as an off-white solid (145 mg, 0.349 mmol, 87%). The corresponding large-scale reaction was conducted according to GP2 on a 15.0 mmol scale (product: 5.13 g, 12.5 mmol, 83%).

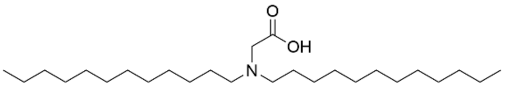

^{1}**H NMR** (400 MHz, $CDCl_3$) δ = 6.66 (bs, 1H), 3.48 (s, 2H), 3.15 – 2.93 (m, 4H), 1.74 – 1.61 (m, 4H), 1.38 – 1.15 (m, 36H), 0.86 (t, *J* = 6.8 Hz, 6H).
^{13}**C NMR** (101 MHz, $CDCl_3$) δ = 167.5, 56.8, 54.4, 32.0, 29.7, 29.6, 29.6, 29.5, 29.3, 26.9, 24.0, 22.8, 14.2. (*Note: One ^{13}C signal is overlapping with another ^{13}C signal and cannot be distinguished.)*
HRMS (ESI) *m/z* calc. for $C_{26}H_{53}NO_2+H^+$: 412.4149 [*M*+H$^+$]; found 412.4157.

GlyC14

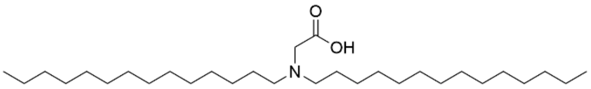

The title compound was prepared according to GP1 on a 0.400 mmol scale and was obtained after silica gel chromatography (CH_2Cl_2:MeOH 1:0 -> 10:1) as an off-white solid (169 mg, 0.362 mmol, 91%). The corresponding large-scale reaction was conducted according to GP2 on a 30.0 mmol scale (product: 12.0 g, 25.7 mmol, 88%).
^{1}H NMR (400 MHz, $CDCl_3$) δ = 6.34 (bs, 1H), 3.49 (s, 2H), 3.19 – 2.98 (m, 4H), 1.73 – 1.62 (m, 4H), 1.36 – 1.16 (m, 44H), 0.87 (t, *J* = 6.9 Hz, 6H).
^{13}C NMR (101 MHz, $CDCl_3$) δ = 167.6, 56.8, 54.4, 32.1, 29.8, 29.8, 29.8, 29.8, 29.7, 29.6, 29.5, 29.3, 26.9, 24.0, 22.8, 14.3
HRMS (ESI) *m/z* calc. for $C_{30}H_{61}NO_2+H^+$: 468.4775 [*M*+H]$^+$; found 468.4775.

ProC8

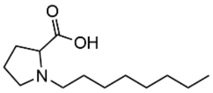

The title compound was prepared according to GP3 on a 0.200 mmol scale and was obtained after silica gel chromatography (CH_2Cl_2:MeOH 1:0 -> 1:1) as an off-white solid (46.3 mg, 0.200 mmol, quant.%). The corresponding large-scale reaction was conducted according to GP4 on a 25.0 mmol scale (product: 4.31 g, 18.9 mmol, 76%).
^{1}H NMR (400 MHz, $CDCl_3$) δ = 8.49 (bs, 1H), 4.02 – 3.92 (m, 1H), 3.76 – 3.58 (m, 1H), 3.20 – 3.08 (m, 1H), 3.02 – 2.90 (m, 1H), 2.83 – 2.72 (m, 1H), 2.39 – 2.24 (m, 1H), 2.22 – 2.12 (m, 1H), 2.02 – 1.90 (m, 2H), 1.77 – 1.63 (m, 2H), 1.33 – 1.11 (m, 10H), 0.80 (t, *J* = 6.8 Hz, 3H)
^{13}C NMR (101 MHz, $CDCl_3$) δ = 170.4, 69.9, 55.8, 54.9, 31.7, 29.5, 29.2, 29.1, 26.8, 25.9, 23.5, 22.6,14.1.
HRMS (ESI) *m/z* calc. for $C_{13}H_{25}NO_2+Na^+$: 250.1778 [*M*+Na]$^+$; found 250.1779.

ProC10

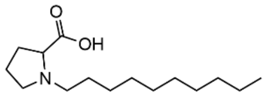

The title compound was prepared according to GP3 on a 0.400 mmol scale and was obtained after silica gel chromatography (CH_2Cl_2:MeOH 10:1 -> 3:1) as an off-white solid (100 mg, 0.393 mmol, 98%).

^{1}H NMR (400 MHz, $CDCl_3$) δ = 9.07 (bs, 1H), 3.99 – 3.88 (m, 1H), 3.68 – 3.61 (m, 1H), 3.17 – 3.05 (m, 1H), 2.99 – 2.87 (m, 1H), 2.80 – 2.69 (m, 1H), 2.36 – 2.21 (m, 1H), 2.19 – 2.07 (m, 1H), 2.01 – 1.83 (m, 2H), 1.76 – 1.63 (m, 2H), 1.36 – 1.05 (m, 14H), 0.78 (t, *J* = 6.8 Hz, 3H).
^{13}C NMR (101 MHz, $CDCl_3$) δ = 170.2, 69.8, 55.7, 54.8, 31.8, 29.4, 29.4, 29.2, 29.2, 29.2, 26.8, 25.8, 23.4, 22.6, 14.0.
HRMS (ESI) *m/z* calc. for $C_{15}H_{29}NO_2+H^+$: 256.2271 [*M*+H]$^+$; found 256.2272.

ProC12

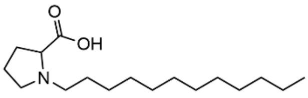

The title compound was prepared according to GP3 on a 0.400 mmol scale and was obtained after silica gel chromatography (CH_2Cl_2:MeOH 10:1 -> 3:1) as an off-white solid (113 mg, 0.397 mmol, 99%).
^{1}H NMR (400 MHz, $CDCl_3$) δ = 7.77 (bs, 1H), 4.04 – 3.94 (m, 1H), 3.76 – 3.66 (m, 1H), 3.22 – 3.11 (m, 1H), 3.05 – 2.93 (m, 1H), 2.87 – 2.75 (m, 1H), 2.42 – 2.27 (m, 1H), 2.25 – 2.14 (m, 1H), 2.06 – 1.89 (m, 2H), 1.79 – 1.69 (m, 2H), 1.38 – 1.13 (m, 18H), 0.84 (t, *J* = 6.8 Hz, 3H).
^{13}C NMR (101 MHz, $CDCl_3$) δ = 170.4, 69.9, 55.9, 55.0, 32.0, 29.7, 29.6, 29.6, 29.5, 29.4, 29.3, 26.9, 25.9, 23.6, 22.7, 14.2. (*Note: One ^{13}C signal is overlapping with another ^{13}C signal and cannot be distinguished.)*
HRMS (ESI) *m/z* calc. for $C_{17}H_{33}NO_2+H^+$: 284.2584 [*M*+H]$^+$; found 284.2584.

ProC14

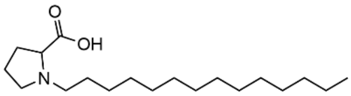

The title compound was prepared according to GP3 on a 0.400 mmol scale and was obtained after silica gel chromatography (CH_2Cl_2:MeOH 1:0 -> 3:1) as an off-white solid (124 mg, 0.399 mmol, 99%).

^{1}H NMR (400 MHz, $CDCl_3$) δ = 8.74 (bs, 1H), 4.02 – 3.92 (m, 1H), 3.70 – 3.63 (m, 1H), 3.20 – 3.08 (m, 1H), 3.01 – 2.89 (m, 1H), 2.85 – 2.71 (m, 1H), 2.38 – 2.24 (m, 1H), 2.23 – 2.10 (m, 1H), 2.04 – 1.86 (m, 2H), 1.75 – 1.66 (m, 2H), 1.33 – 1.15 (m, 22H), 0.81 (t, *J* = 6.8 Hz, 3H).
^{13}C NMR (101 MHz, $CDCl_3$) δ = 170.3, 69.9, 55.8, 54.9, 31.9, 29.7, 29.7, 29.6, 29.6, 29.5, 29.5, 29.4, 29.4, 29.2, 26.8, 25.9, 23.5, 22.7, 14.1.
HRMS (ESI) *m/z* calc. for $C_{19}H_{37}NO_2$+H^+: 312.2897 [*M*+H]$^+$; found 312.2899.

ProC16

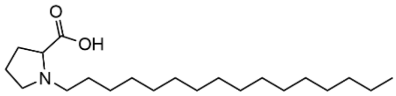

The title compound was prepared according to GP3 on a 0.400 mmol scale and was obtained after silica gel chromatography (CH_2Cl_2:MeOH 1:0 -> 3:1) as an off-white solid (132 mg, 0.388 mmol, 97%).

^{1}H NMR (400 MHz, $CDCl_3$) δ = 9.52 (bs, 1H), 3.98 – 3.88 (m, 1H), 3.64 (t, *J* = 8.1 Hz, 1H), 3.16 – 3.05 (m, 1H), 2.98 – 2.86 (m, 1H), 2.80 – 2.68 (m, 1H), 2.35 2.21 (m, 1H), 2.18 – 2.05 (m, 1H), 1.98 – 1.82 (m, 2H), 1.74 – 1.62 (m, 2H), 1.27 – 1.11 (m, 26H), 0.76 (t, *J* = 6.8 Hz, 3H).
^{13}C NMR (101 MHz, $CDCl_3$) δ = 170.3, 69.7, 55.7, 54.8, 31.8, 29.6, 29.6, 29.6, 29.6, 29.5, 29.5, 29.4, 29.3, 29.3, 29.1, 26.7, 25.8, 23.4, 22.6, 14.0.(*Note: One ^{13}C signal is overlapping with another ^{13}C signal and cannot be distinguished.)*
HRMS (ESI) *m/z* calc. for $C_{21}H_{41}NO_2$+H^+: 340.3210 [*M*+H]$^+$; found 340.3213.

ProC18

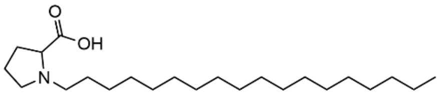

The title compound was prepared according to GP3 on a 0.400 mmol scale and was obtained after silica gel chromatography (CH_2Cl_2:MeOH 20:1 -> 4:1) as an off-white solid (146 mg, 0.397 mmol, 99%).

^{1}H NMR (400 MHz, $CDCl_3$) δ = 7.61 (bs, 1H), 4.05 – 3.94 (m, 1H), 3.72 – 3.65 (m, 1H), 3.22 – 3.11 (m, 1H), 3.03 – 2.92 (m, 1H), 2.84 – 2.74 (m, 1H), 2.41 – 2.27 (m, 1H), 2.26 – 2.16 (m, 1H), 2.06 – 1.89 (m, 2H), 1.80 – 1.70 (m, 2H), 1.42 – 1.07 (m, 30H), 0.84 (t, *J* = 6.6 Hz, 3H).
^{13}C NMR (101 MHz, $CDCl_3$) δ = 170.4, 70.0, 55.9, 55.0, 32.0, 29.8, 29.8, 29.8, 29.7, 29.7, 29.6, 29.6, 29.5, 29.4, 29.3, 26.9, 26.0, 23.6, 22.8, 14.2. (*Note: Three ^{13}C signals are overlapping with other ^{13}C signals and cannot be distinguished.)*
HRMS (ESI) *m/z* calc. for $C_{23}H_{45}NO_2$+H^+: 368.3532 [*M*+H]$^+$; found 368.3522.

AlaC6

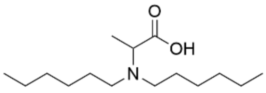

The title compound was prepared according to GP6 on a 10.0 mmol scale and was obtained after silica gel chromatography (CH_2Cl_2:MeOH 95:5 -> 9:1) as an off-white solid (0.708 g, 2.75 mmol, 26%).

^{1}H NMR (600 MHz, $CDCl_3$) δ = 6.58 (bs, 1H), 3.60 (q, *J* = 7.1 Hz, 1H), 3.11 – 3.03 (m, 2H), 2.96 – 2.87 (m, 2H), 1.77 – 1.69 (m, 2H), 1.67 – 1.57 (m, 2H), 1.47 (d, *J* = 7.1 Hz, 3H), 1.35 – 1.23 (m, 12H), 0.87 (t, *J* = 6.9 Hz, 6H).
^{13}C NMR (151 MHz, $CDCl_3$) δ = 170.8, 62.9, 51.1, 31.4, 26.7, 24.3, 22.6, 14.0, 12.6.
HRMS (ESI) *m/z* calc. for $C_{15}H_{31}NO_2$+H^+: 258.2428 [*M*+H]$^+$; found 258.2426.

AlaC8

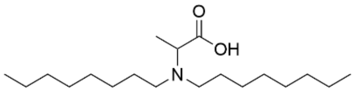

The title compound was prepared according to GP5 on a 0.400 mmol scale and was obtained after silica gel chromatography (CH_2Cl_2:MeOH 1:0 -> 10:1) as an off-white solid (110 mg, 0.350 mmol, 87%). The corresponding large-scale reaction was conducted according to GP6 on a 10.0 mmol scale (product: 2.31 g, 7.37 mmol, 74%).

^{1}H NMR (600 MHz, $CDCl_3$) δ = 9.07 (bs, 1H), 3.54 (q, *J* = 7.3 Hz, 1H), 3.08 – 2.96 (m, 2H), 2.94 – 2.81 (m, 2H), 1.73 – 1.48 (m, 4H), 1.39 (d, *J* = 6.9 Hz, 3H), 1.29 – 1.02 (m, 20H), 0.78 (t, *J* = 6.8 Hz, 6H).
^{13}C NMR (151 MHz, $CDCl_3$) δ = 170.7, 62.7, 50.9, 31.6, 29.1, 29.1, 26.9, 24.0, 22.5, 14.0, 12.8.
HRMS (ESI) *m/z* calc. for $C_{19}H_{39}NO_2$+H^+: 314.3054 [*M*+H]$^+$; found 314.3058.

ValC8

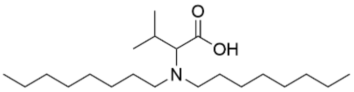

The title compound was prepared according to GP5 on a 0.400 mmol scale and was obtained after silica gel chromatography (CH_2Cl_2:MeOH 1:0 -> 10:1) as an off-white solid (76.0 mg, 0.202 mmol, 51%).

^{1}H NMR (400 MHz, $CDCl_3$) δ = 9.77 (bs, 1H), 3.25 (d, *J* = 5.9 Hz, 1H), 2.98 – 2.69 (m, 4H), 2.16 – 1.96 (m, 1H), 1.64 – 1.47 (m, 4H), 1.32 – 1.19 (m, 20H), 1.10 (d, *J* = 6.6 Hz, 3H), 0.97 (d, *J* = 6.6 Hz, 3H), 0.85 (t, *J* = 6.7 Hz, 6H).
^{13}C NMR (101 MHz, $CDCl_3$) δ = 170.9, 72.1, 50.2, 32.1, 29.3, 27.0, 26.6, 25.1, 22.6, 20.6, 18.2, 14.1
HRMS (ESI) *m/z* calc. for $C_{21}H_{43}NO_2$+H^+: 342.3367 [*M*+H]$^+$; found 342.3371.

LeuC8

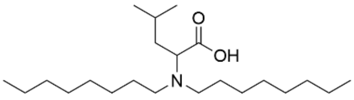

The title compound was prepared according to GP5 on a 0.400 mmol scale and was obtained after silica gel chromatography (CH_2Cl_2:MeOH 1:0 -> 10:1) as an off-white solid (85.3 mg, 0.240 mmol, 60%).

^{1}H NMR (600 MHz, $CDCl_3$) δ = 6.43 (bs, 1H), 3.47 (dd, *J* = 10.2, 4.1 Hz, 1H), 3.05 – 2.99 (m, 2H), 2.94 – 2.88 (m, 2H), 2.02 – 1.92 (m, 1H), 1.90 – 1.83 (m, 1H), 1.73 – 1.56 (m, 4H), 1.35 – 1.20 (m, 21H), 0.99 – 0.93 (m, 6H), 0.87 (t, *J* = 7.0 Hz, 6H)
^{13}C NMR (151 MHz, $CDCl_3$) δ = 170.8, 66.0, 50.9, 36.2, 31.8, 29.3, 29.2, 27.0, 25.8, 24.6, 23.8, 22.7, 21.9, 14.2.
HRMS (ESI) *m/z* calc. for $C_{22}H_{45}NO_2$+H^+: 356.3523 [*M*+H]$^+$; found 356.3527.

PheC8

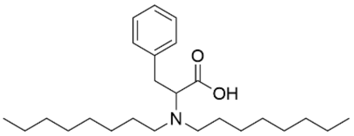

The title compound was prepared according to GP5 on a 0.400 mmol scale and was obtained after silica gel chromatography (CH_2Cl_2:MeOH 1:0 -> 10:1) as an off-white solid (97.2 mg, 0.249 mmol, 62%).

^{1}H NMR (400 MHz, $CDCl_3$) δ = 8.87 (bs, 1H), 7.24 – 7.16 (m, 4H), 7.16 – 7.04 (m, 1H), 3.82 (dd, *J* = 8.2, 5.1 Hz, 1H), 3.58 – 3.46 (m, 1H), 2.96 – 2.84 (m, 3H), 2.72 – 2.61 (m, 2H), 1.53 (m, 2H), 1.46 – 1.32 (m, 2H), 1.24 – 1.06 (m, 20H), 0.77 (t, *J* = 6.9 Hz, 6H).j
^{13}C NMR (101 MHz, $CDCl_3$) δ = 170.6, 138.2, 128.9, 128.8, 126.9, 67.7, 52.0, 33.8, 31.8, 29.2, 26.9, 25.3, 22.7, 14.1. (*Note: One ^{13}C signal is overlapping with another ^{13}C signal and cannot be distinguished.)*
HRMS (ESI) *m/z* calc. for $C_{25}H_{43}NO_2$+H^+: 390.3367 [*M*+H]$^+$; found 390.3372.

LysC8

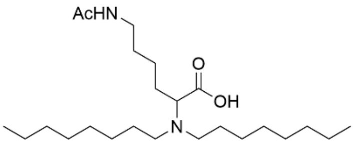

The title compound was prepared according to GP5 on a 0.4 mmol scale and was obtained after silica gel chromatography (CH_2Cl_2:MeOH 1:0 -> 5:1) as an off-white solid (139 mg, 0.338 mmol, 85%). The corresponding large-scale reaction was conducted according to GP6 on a 5 mmol scale (product: 1.80 g, 4.36 mmol, 87%).

^{1}H NMR (400 MHz, $CDCl_3$) δ = 9.24 (bs, 1H), 7.03 (bs, 1H), 3.42 – 3.34 (m, 1H), 3.25 – 2.83 (m, 6H), 1.90 (s, 3H), 1.78 – 1.32 (m, 10H), 1.31 – 1.09 (m, 20H), 0.79 (t, *J* = 6.7 Hz, 6H).
^{13}C NMR (101 MHz, $CDCl_3$) δ = 170.6, 170.2, 67.3, 50.4, 38.8, 31.6, 29.1, 29.1, 28.9, 26.9, 26.6, 24.1, 23.6, 23.1, 22.5, 14.0.
HRMS (ESI) *m/z* calc. for $C_{24}H_{48}N_2O_3+H^+$: 413.3738 [*M*+H]$^+$; found 413.3743.

GemC2

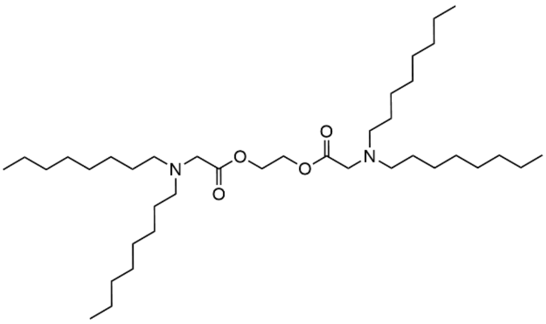

The title compound was prepared according to GP7 on a 0.500 mmol scale and was obtained after silica gel chromatography (pentane:EtOAc 100:1 -> 10:1) as a colorless oil (0.207 g, 0.332 mmol, 66%). The corresponding large-scale reaction was performed according to GP5 on a 10.0 mmol scale (product: 5.12 g, 8.19 mmol, 85%).

^{1}H NMR (400 MHz, $CDCl_3$) δ = 4.30 (s, 4H), 3.34 (s, 4H), 2.59 – 2.51 (m, 8H), 1.47 – 1.39 (m, 8H), 1.34 – 1.19 (m, 40H), 0.87 (t, *J* = 6.7 Hz, 12H).
^{13}C NMR (101 MHz, $CDCl_3$) δ = 171.6, 62.0, 55.0, 54.6, 32.0, 29.7, 29.5, 27.7, 27.6, 22.8, 14.2.
HRMS (ESI) *m/z* calc. for $C_{38}H_{76}N_2O_4+H^+$: 625.5878 [*M*+H]$^+$; found 625.5880.

GemC4

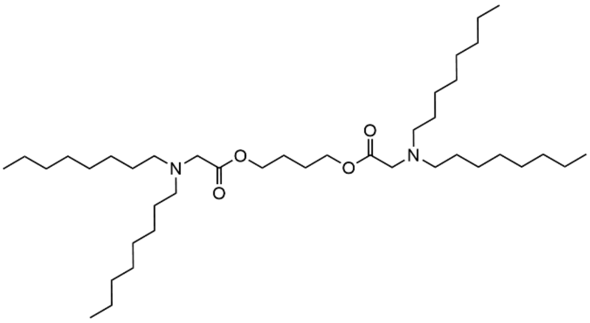

The title compound was prepared according to GP7 on a 0.500 mmol scale and was obtained after silica gel chromatography (pentane:EtOAc 20:1 -> 10:1) as a colorless oil (289 mg, 0.442 mmol, 88%).

^{1}H NMR (400 MHz, $CDCl_3$) δ = 4.14 – 4.04 (m, 4H), 3.28 (s, 4H), 2.55 – 2.47 (m, 8H), 1.72 – 1.64 (m, 4H), 1.44 – 1.34 (m, 8H), 1.31 – 1.16 (m, 40H), 0.84 (t, *J* = 6.7 Hz, 12H).
^{13}C NMR (101 MHz, $CDCl_3$) δ = 171.8, 63.7, 55.1, 54.6, 31.9, 29.6, 29.4, 27.6, 27.5, 25.5, 22.7, 14.2.
HRMS (ESI) *m/z* calc. for $C_{40}H_{80}N_2O_4+H^+$: 653.6191 [*M*+H]$^+$; found 653.6198.

GemC6

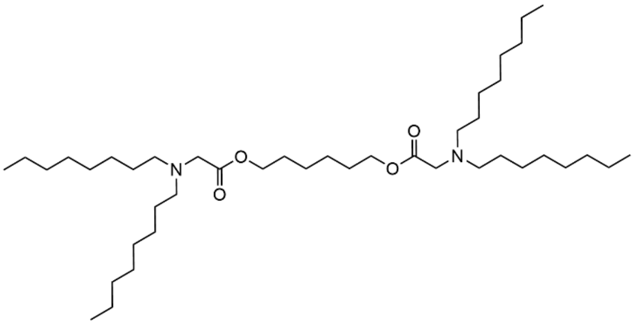

The title compound was prepared according to GP7 on a 0.500 mmol scale and was obtained after silica gel chromatography (pentane:EtOAc 40:1 -> 10:1) as a colorless oil (304 mg, 0.446 mmol, 89%).

^{1}H NMR (400 MHz, $CDCl_3$) δ = 4.09 (t, *J* = 6.7 Hz, 4H), 3.31 (s, 4H), 2.58 – 2.50 (m, 8H), 1.68 – 1.60 (m, 4H), 1.47 – 1.33 (m, 12H), 1.33 – 1.19 (m, 40H), 0.87 (t, *J* = 6.8 Hz, 12H).
^{13}C NMR (101 MHz, $CDCl_3$) δ = 172.0, 64.3, 55.3, 54.6, 32.0, 29.7, 29.4, 28.7, 27.6, 27.6, 25.8, 22.8, 14.2.
HRMS (ESI) *m/z* calc. for $C_{42}H_{84}N_2O_4$+H^+: 681.6504 [*M*+H]$^+$; found 681.6506.

GemGlyc

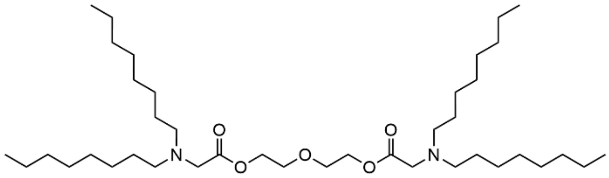

The title compound was prepared according to GP5 on a 0.500 mmol scale and was obtained after silica gel chromatography (pentane:EtOAc 20:1 -> 8:1) as a colorless oil (0.300 g, 0.448 mmol, 90%). The corresponding large-scale reaction was performed according to GP7 on a 10 mmol scale (product: 5.19 g, 7.76 mmol, 97%).
^{1}H NMR (400 MHz, $CDCl_3$) δ = 4.28 – 4.21 (m, 4H), 3.72 – 3.65 (m, 4H), 3.35 (s, 4H), 2.59 – 2.47 (m, 8H), 1.47 – 1.37 (m, 8H), 1.33 – 1.20 (m, 40H), 0.87 (t, *J* = 6.9 Hz, 12H).
^{13}C NMR (101 MHz, $CDCl_3$) δ = 171.7, 69.2, 63.2, 55.1, 54.6, 32.0, 29.7, 29.5, 27.7, 27.6, 22.8, 14.2.
HRMS (ESI) *m/z* calc. for [$C_{40}H_{80}N_2O_5$+H^+: 669.6140 [*M*+H]$^+$;found 669.6144.

GlyC8*EtBr

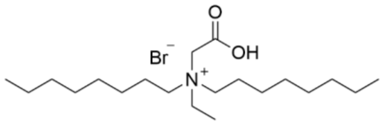

Dioctylglycine (4.20 g, 14.0 mmol), MeCN (not dry, 35.0 mL) and bromoethane (2.10 mL, 28.1 mmol) were added to a screw-cap vial under air. The reaction mixture was allowed to stir at 90 °C overnight. Volatile compounds were removed under reduced pressure and the desired product was obtained as a yellow solid (5.55 g, 13.6 mmol, 97%).

^{1}H NMR (400 MHz, $CDCl_3$) δ = 11.60 (bs, 1H), 4.28 (q, *J* = 7.1 Hz, 2H), 3.93 – 3.90 (m, 2H), 3.36 – 3.19(m, 4H), 2.04 – 1.89 (m, 2H), 1.85 – 1.70 (m, 2H), 1.49 – 1.08 (m, 23H), 0.87 (t, *J* = 6.8 Hz, 6H).
^{13}C NMR (101 MHz, $CDCl_3$) δ = 164.7, 62.7, 54.3, 50.0, 31.6, 29.0, 28.9, 26.8, 24.5, 22.6, 14.1, 14.0.
HRMS (ESI) *m/z* calc. for $BrC_{20}H_{40}NO_2^-$: 406.2326 [*M*-H]$^-$, found 406.2324.

2. CMC Determination

The critical micelle concentration (CMC) was determined by pending drop tensiometry using a Bioline Theta Lite Optical Tensiometer. For sample preparation, a stock solution of the surfactant was prepared by suspending the surfactant in deionized water, addition of conc. aqueous NaOH or aqueous HCl solution – depending on the indicated pH at which the measurement was taken – and vigorous shaking. Once all solids or droplets were dissolved, the pH was adjusted by addition of a 1 M. aqueous solution of NaOH or HCl, while the pH value was checked using a pH meter. Subsequently, the stock solution was diluted to various concentrations by addition of an aqueous solution with pH = 2, 5, 7 or 9, respectively. All tensiometric measurements were performed as triplicates, using their mean value as the surface tension for CMC determination. The CMC was determined by plotting the surface tension against the surfactant concentration and calculation of the intersection between the regression curve corresponding to the lower plateau of the s-shaped curve (blue line) and the regression curve corresponding to the slope of the s-shaped curve (red line).

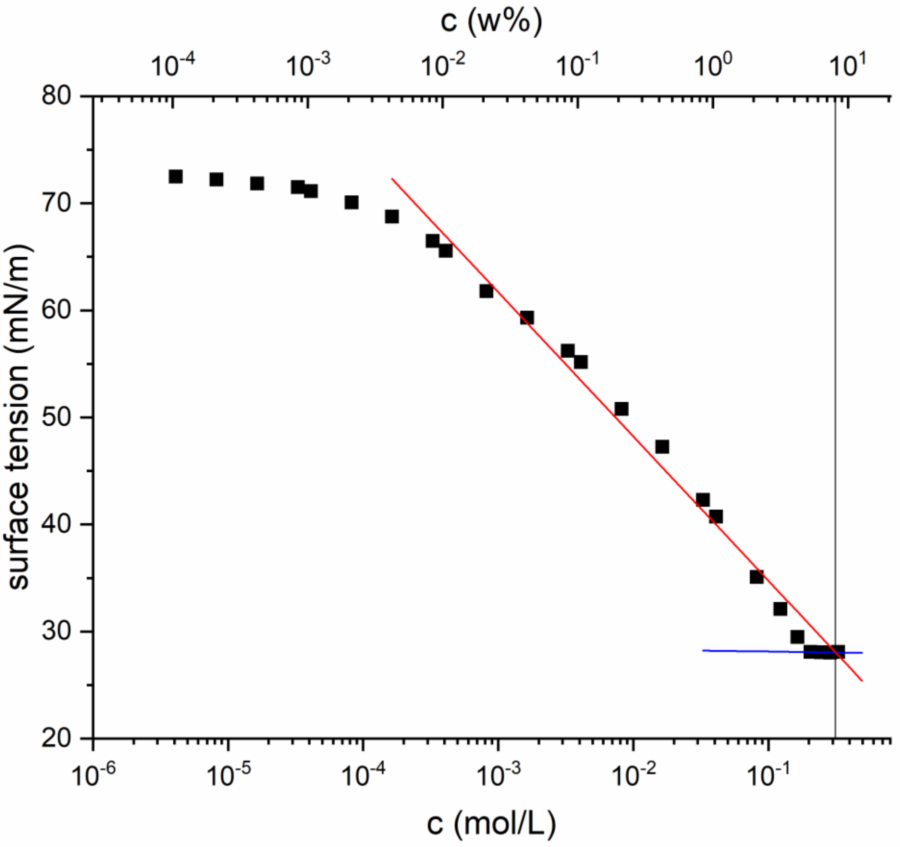

Figure S3: CMC determination of **GlyC6**.

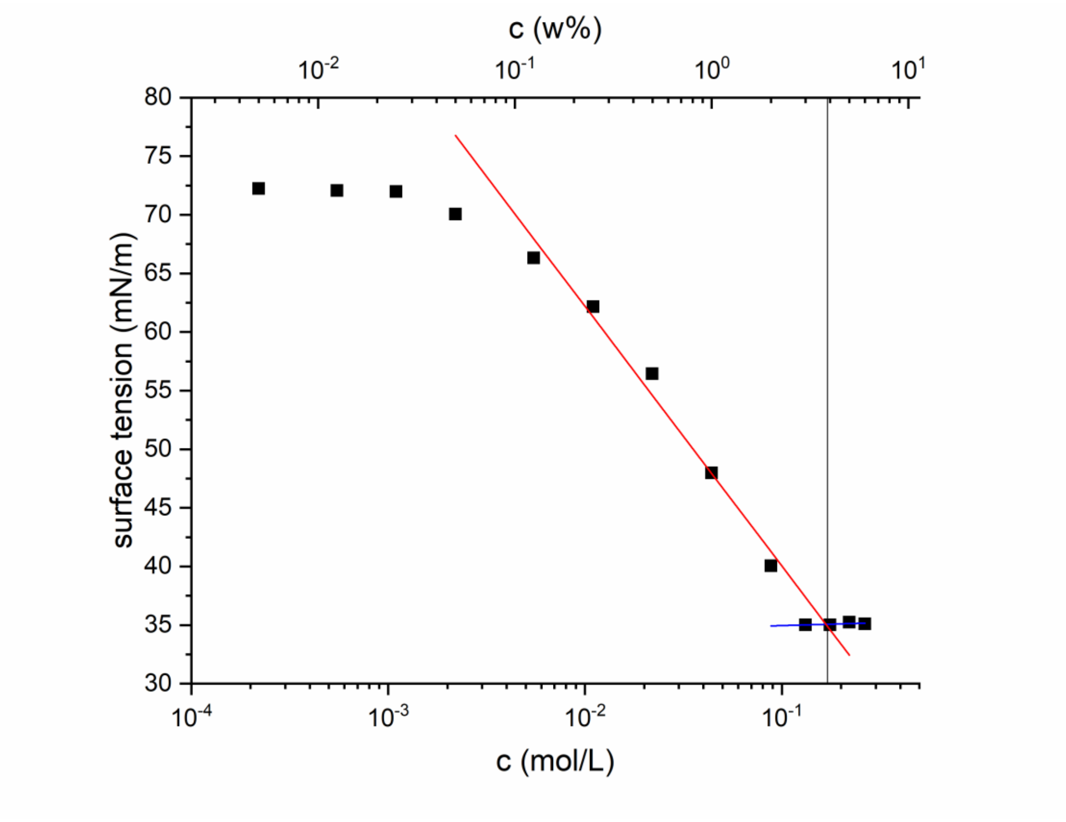

Figure S4: CMC determination of **ProC8**.

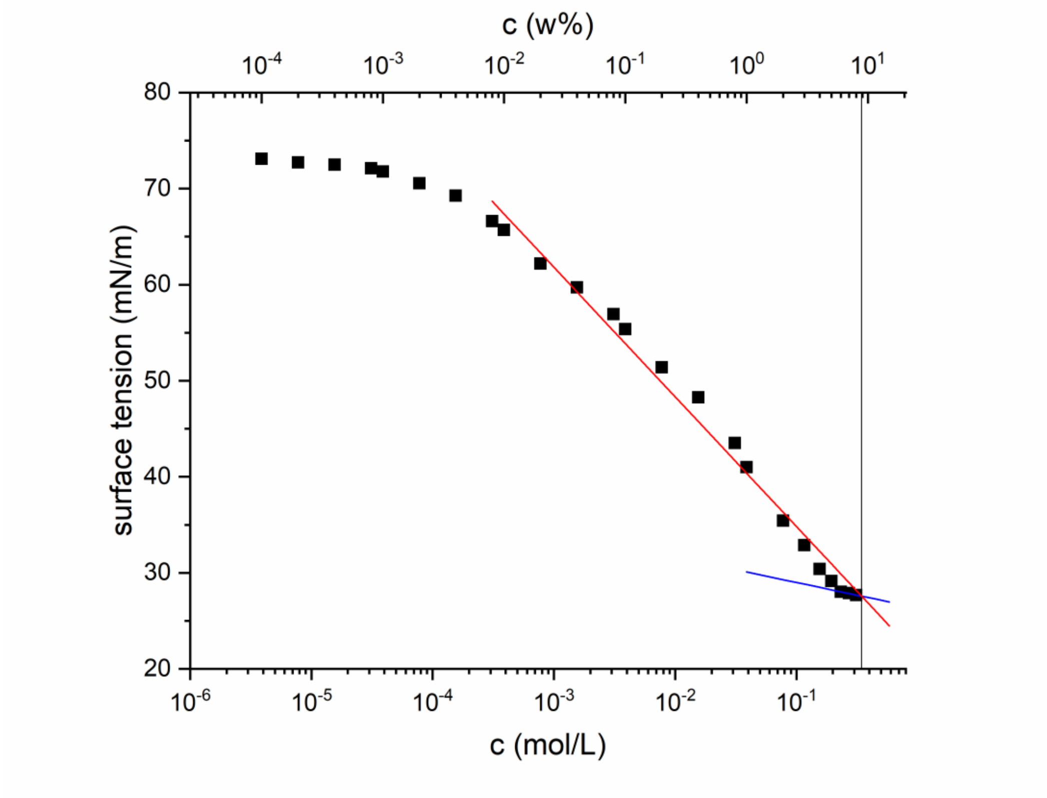

Figure S5: CMC Determination of **AlaC6**.

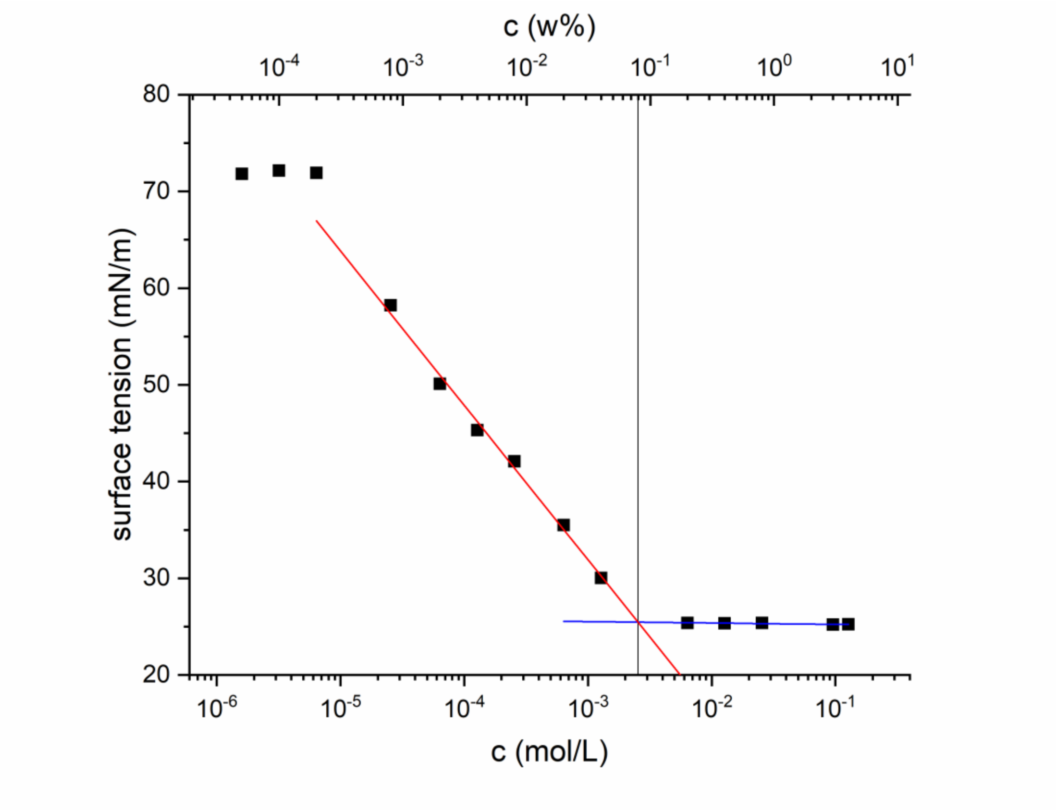

Figure S6: CMC determination of **AlaC8**.

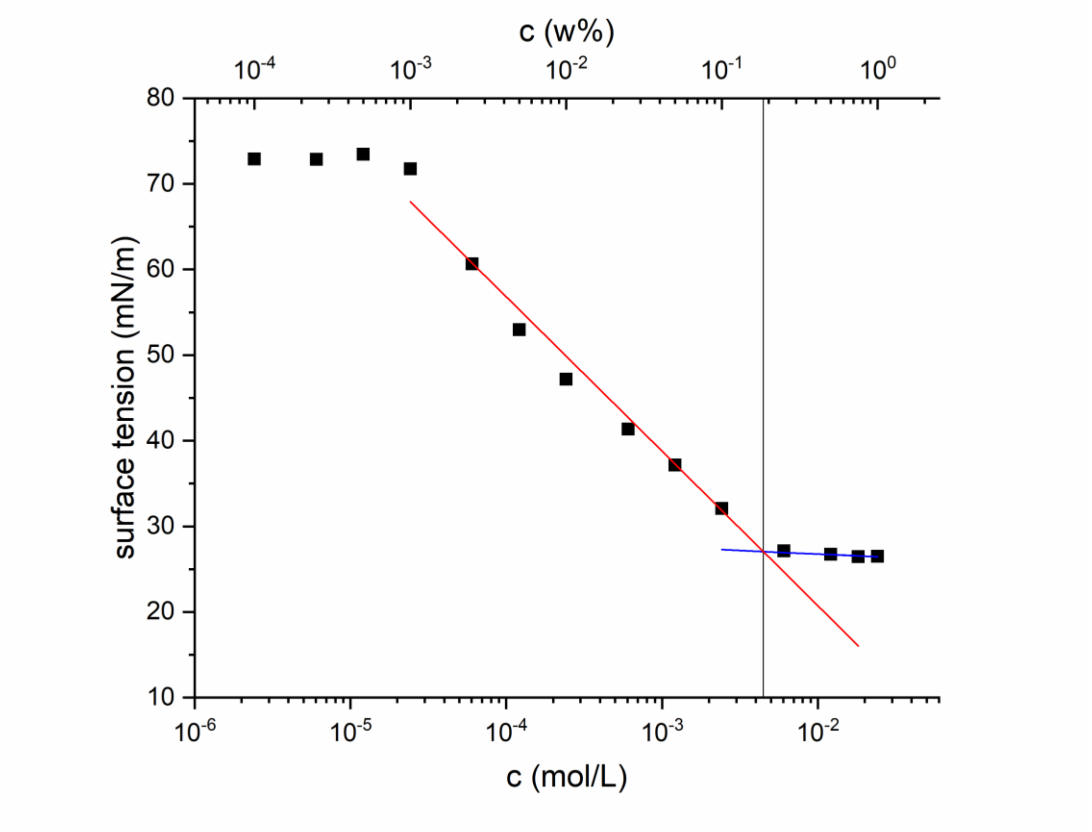

Figure S7: CMC determination of **LysC8** at pH = 5.

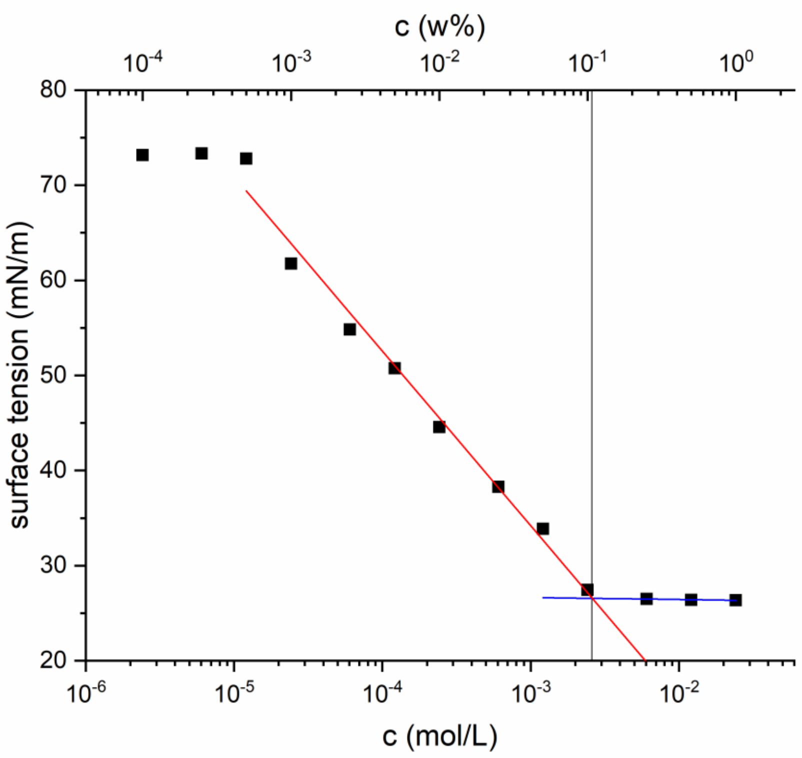

Figure S8: CMC determination of **LysC8** at pH = 7.

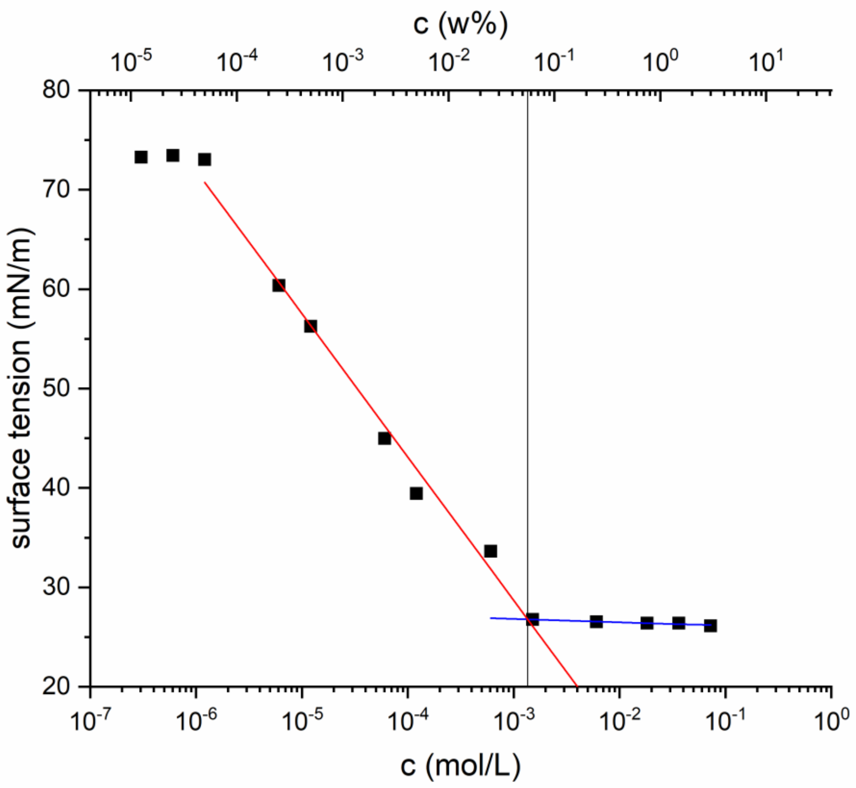

Figure S9: CMC determination of **LysC8** at pH = 9.

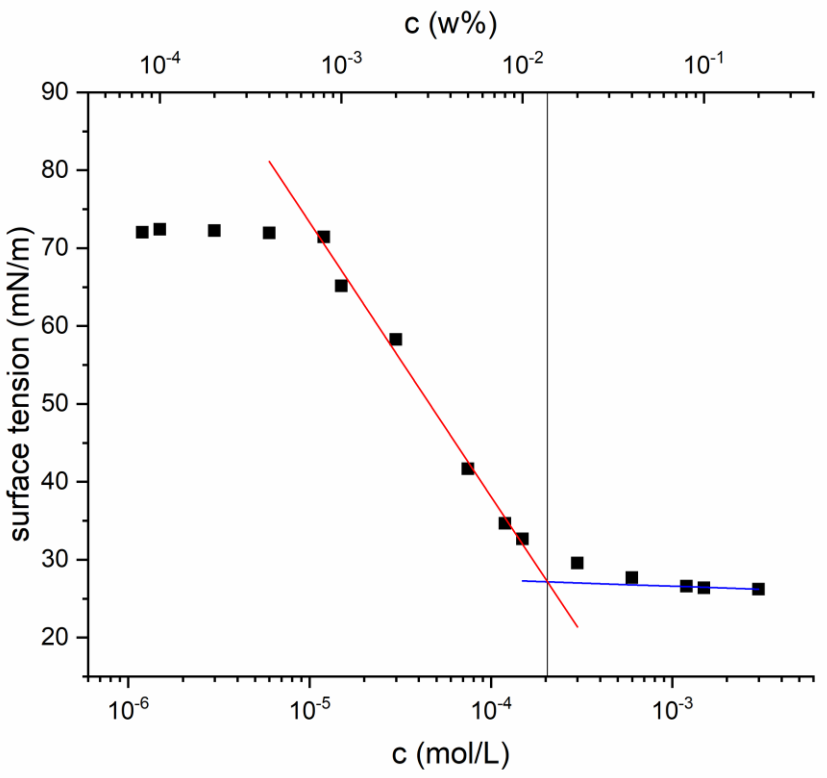

Figure S10: CMC determination of **GemGlyc**.

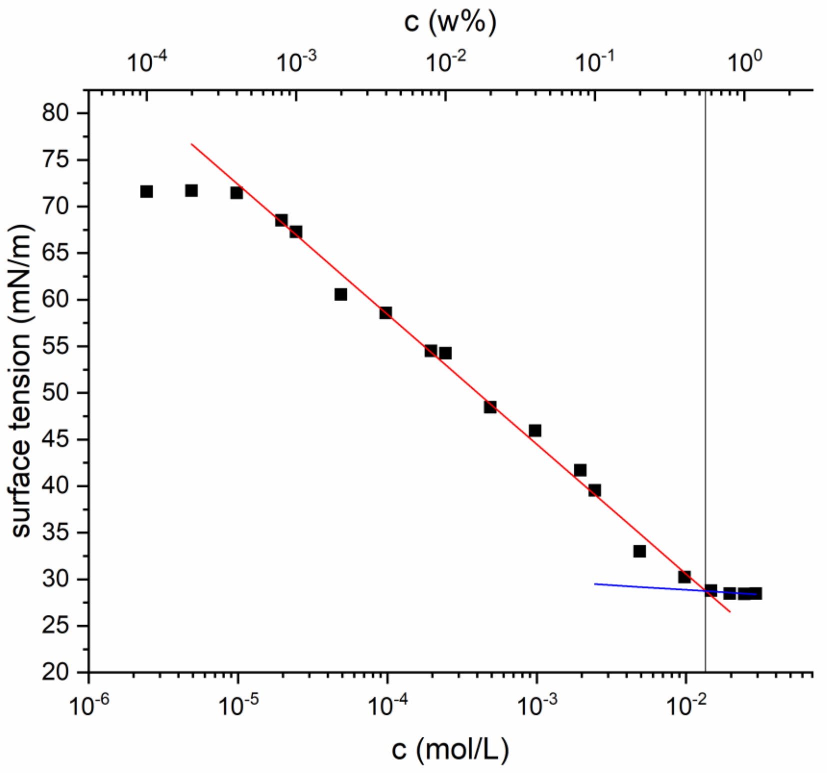

Figure S11: CMC determination of **GlyC8*EtBr**.

3. Foaming Analysis

The foaming behavior of the surfactants was analyzed by means of their foaming height h_0 and their foaming half-life time $t_{1/2}$. These values were determined as follows: A stock solution of the surfactant with concentration above the CMC was prepared. 0.5 mL of this solution were transferred into a 4 mL screw cap vial. The vial was shaken for 10 s using a Vortex Genie 2 on highest intensity. Subsequently, each vial was immediately positioned in front of a LED backlight screen and a photo was taken using a Dino Lite Digital Microscope. Over the course of 120 min, one picture was taken per minute. All samples were measured as triplicates. All pictures were analyzed using the software *ImageJ*. The initial foaming height h_0 is defined as the ratio between the height of the foam at t = 0 min h_{foam} and the overall height of the vial h_{vial}. The half-life time is defined as the time, after which h_{foam} has reached half of its value, e.g. $h_{foam1/2}$.

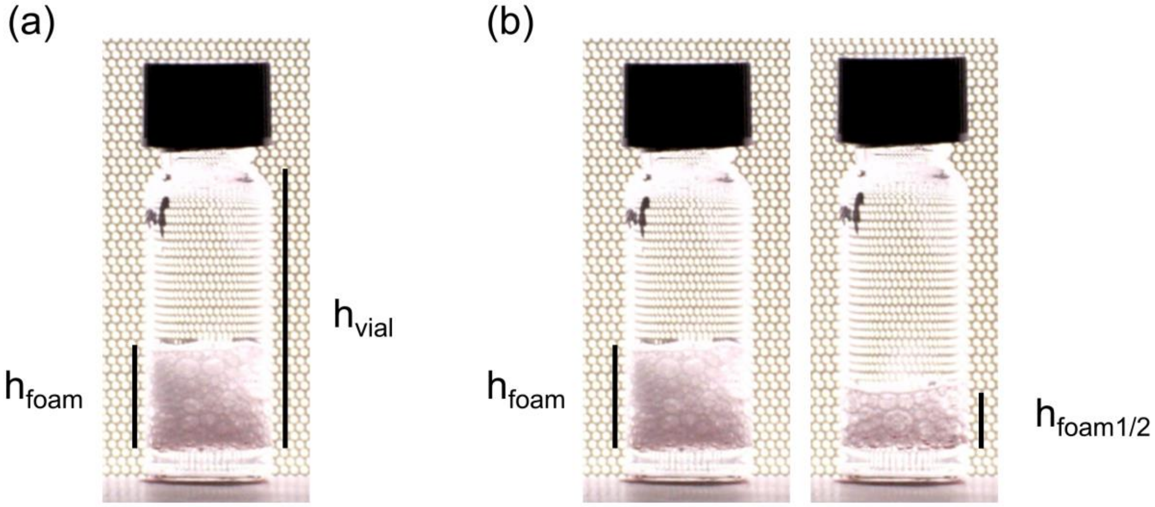

Figure S12: Foaming analysis measures: (a) initial foaming height (b) foaming half-life time.

4. Biodegradability Assay

AlaC8

Biodegradability of **AlaC8** was determined using the standardized manometric assay OECD 301F test,[42], using OxiTop®-IDS sensor available from Xylem Analytics (France). The inoculum used in this experiment was composed of a washed activated sludge sampled from La Courly, a wastewater treatment plant from Pierre-Bénite (France), with the GPS coordinates: 45.694917, 4.836628. The inoculum was sampled on August 18th, 2022, and was aerated under agitation overnight to decrease organic carbon content and consequently endogenous respiration as preconized by the OECD 301 guideline.

After overnight incubation, a final sample preparation was performed to remove the remaining excessive organic carbon. To do this, three successive centrifugation steps (6000 rpm, 15 min at room temperature) were done to remove the liquid content and resuspend the inoculum at 5 g L^{-1} of MLSS (mixed liquor suspended solids) in the mineral medium of the biodegradation assay. The composition of the medium of the biodegradation assay was 85 mg L^{-1} KH_2PO_4, 217.5 mg L^{-1} K_2HPO_4, 334 mg L^{-1} Na_2HPO_4, 27.5 mg L^{-1} $CaCl_2$, 11.5 mg L^{-1} $MgSO_4$ and 0.100 mg L^{-1} $FeCl_3$. The pH of the medium was adjusted to 7.2 by the addition of a 1 M aqueous solution of HCl.

A stock solution was prepared by overnight agitation (250 rpm) at room temperature of the sample at a final concentration of 1 g L^{-1} in deionized water. A biodegradation assay was performed by incubating the washed inoculum at a final concentration of 26.7 mg MLSS L^{-1} in the presence of the sample at an equivalent of 69 mg O_2 L^{-1} consumed to mineralize the totality of the sample expressed in ThOD, respectively. Biodegradation was assessment in BOD flasks incubated at 21 °C +/-1 °C using the TS608/4i incubation chambers (Xylem Analytics; France). Biodegradation measurements are based on the variation of the atmospheric pressure, which was registered daily with the use of the OxiTop®-IDS sensors. Biodegradability was then expressed in percentage according to the quantity of oxygen required to mineralize the sample. Additional testing conditions were carried out using a reference substance used in the assay to conclude both on the functionality of the inoculum and about a non-relevant or low toxicity of the sample. To confirm the functionality of the inoculum, biodegradability of starch was followed at a final concentration of 52 mg O_2 L^{-1} ThOD as the reference control. Finally, to confirm the non-toxicity of the sample both starch and the item were added at the initial concentration, in an assay and the biodegradability of the mixture was assessed as the toxicity control.

Biodegradability of the sample was assessed in an enhanced ready biodegradability test, meaning that the biodegradation was followed more than 28 days and less than 60 days, assay stopped after 45 days. During the procedure, a set of five quality criteria were assessed to status on the quality of the biodegradation assessment. The reference control highlighted an adequate functionality of the inoculum which degraded more than 62.7% (ThOD) of the starch in less than 14 days with the pass-level fixed at a minimum of 60%. The sample appeared as non-toxic as the toxicity control at day 14 is 36.1% (ThOD) and thus exceeding the lower biodegradability limit of 25% for non-toxic samples. The repeatability of the replicates at 2.1% is in accordance with the guideline as well as the respiration of the sludge without any carbon sources at 17 mg O_2 L^{-1}, lower than 30 mg O_2 L^{-1} at the end of the incubation period of 28 days as needed for a valid assay. The pH at the end of the assay was 7.1 which is in accordance with the accepted range of pH = 6.0 - 8.5.

After a latency phase of 7 days the biodegradability of the sample started and presented two consecutive biodegradation kinetics: (i) a fast kinetic ranging from 4.5% to ~29% in 4 days (day 7 to 11), (ii) a slow kinetic ranging from 31.7% to 66.5% in 21 days (day 18 to 39), respectively in ThOD. After 39 days, the biodegradation stabilized and indicates ultimate biodegradability under the stringency of the OECD 301F guideline in less than 60 days. Therefore. In alignment with OECD Guidelines for the Testing of Chemicals revised version,[44] the test item can be considered as inherent ultimate biodegradable as it is necessary to extend the test from 28 days to 60 days.

LysC8

Biodegradability of **LysC8** was determined using the standardized manometric assay OECD 301F test,[2] using the OxiTop® Control measuring system, consisting of the OxiTop® OC110 controller, the OxiTop® -C measuring heads, the PF600 measuring bottles, the IS12 stirring unit, and the TS606- 6/4-i thermostat cabinet from WTW (Germany). The inoculum used in this experiment was composed of a washed activated sludge sampled from and provided by the sewage treatment plant Shanghai (China). The inoculum was aerated under agitation overnight to decrease organic carbon content and consequently endogenous respiration as preconized by the OECD 301 guideline.

After overnight incubation, a final sample preparation was performed to remove the remaining excessive organic carbon. The aerobic activated sludge used for this study was deposited for 15 min, washed by centrifugation and the supernatant liquid phase was decanted. The solid material was re-suspended in test water and centrifuged again. This procedure was done three times. An aliquot of the final sludge suspension was weighed, dried and the ratio of wet sludge to its dry weight was determined. Based on this ratio calculated aliquots of washed sludge suspension corresponding to 4.0 g dry material per liter were mixed with test water and aerated overnight. This suspension was used for the experiment.

The composition of the medium of the biodegradation assay was assessed using analytical grade salts added to ultrapure water to prepare the following stock solutions:

a) 8.5 g KH_2PO_4, 21.75 g K_2HPO_4, 33.4 g $Na_2HPO_4•2H_2O$, 0.5 g NH_4Cl filled up with pure water to 1000 mL volume; the pH-value was 7.4

b) 11.25 g $MgSO_4•7\ H_2O$ filled up with pure water to 500 mL volume

c) 18.2 g $CaCl_2•2\ H_2O$ filled up with pure water to 500 mL volume

d) 0.125 g $FeCl_3•6\ H_2O$ filled up with pure water to 500 mL volume

40 mL of stock solution a) and 4 mL each of the stock solutions b) to d) were combined and filled up to a final volume of 4000 mL with ultrapure water, the pH was controlled to 7.4.

The test flasks were prepared as described above and were incubated at 22°C ± 2°C. The pressure decrease in the reaction vessels was measured over the complete experimental phase of 28 days (In this test, the test period was extended to 60 days). The test flasks were closed gas-tight by a measuring head. Sodium hydroxide was used for trapping the produced carbon dioxide. The amount of O_2 consumed by the activated sludge was calculated from the decrease of pressure in the reaction vessel.

The purpose of this study was to determine the ready biodegradability of the test item **LysC8** in a ready biodegradability screening test using test concentrations (25 mg/L, 64 mg ThOD/L). The test item was exposed to aerobic-activated sludge from the aeration tank of a domestic wastewater treatment plant for 28 days (The study may be prolonged beyond 28 days if the biodegradation has started but has not yet reached a plateau). The biodegradation was followed by the oxygen uptake of the microorganisms during exposure. The extent of biodegradation achieved within 28 days should be used for the evaluation of ready biodegradability and degradation after 28 days would allow the test substance to be classified as inherently biodegradable. As a reference item starch was tested simultaneously under the same conditions as the test item and functioned as an activity control.

The oxygen uptake of the inoculum blank is normally 20-30 mg O_2/L and should not be greater than 60 mg/l in 28 days. Values higher than 60 mg/l require critical examination of the data and experimental technique. If the pH value is outside the range 6.0-8.5 and the oxygen consumption by the test substance is less than 60%, the test should be repeated with a lower concentration of test substance.

The mean biodegradation percentage at the end of the 28-day and 60-day exposure period was 42.2% and 84.4%, respectively (mean value, based on ThOD). The reference item starch was sufficiently degraded to 61.3% after 10 days and to 86.0% after 60 days of incubation. The percentage biodegradation of the reference item confirms the suitability of the used aerobic activated sludge

inoculum. A toxicity control (test substance in combination with the positive control substance) was also studied at a mean combined concentration of 75 mg/L. The guideline[2] states that a test substance will be considered inhibitory if the toxicity test systems, containing both the test and positive control substance, reach less than 25% biodegradation by day 14. The toxicity control systems exceeded 25% on day 9, which suggests that the test substance was not inhibitory at the tested concentration of approximately 25 mg/L.

The 28-day biodegradation of LysC8 was 42.2% and the 60-day biodegradation was 84.4% on 25 mg/L in an OECD 301F test. This test substance was not readily but ultimately inherently biodegradable according to the OECD guideline[2] definition.

5. Computational Studies

Atomistic Model and Interaction Parameters:

Atomistic molecular dynamics (MD) simulations were carried out for **AlaC8** and **GlyC8** in zwitterionic (pH = 7) and -1 states (pH = 9), totaling four sets of simulations. Each set includes the calculation of solvation free energy (*ΔF*) and the potential of mean force (PMF) for two-molecule fusion. The simulations used the GROningen MAchine for Chemical Simulations (GROMACS) package[45], with the initial configuration and topology files generated using the CGenFF tool[46]. The surfactants were modeled using the CHARMM36 forcefield[46] and water was modeled using the TIP3P model[47]. The partial charges of the surfactant molecules were refined through DFT calculations using the B3LYP functionals and the 6-311++G(d,p) basis set, performed with GAUSSIAN 09 program[48]. The Polarizable Continuum Model (PCM)[49] using water as the solvent was applied. The charge fitting used the ChElPG calculation scheme. The resulting partial charges were averaged over two symmetric -C_8 tails. In MD simulation, the Lorentz-Berthelot mixing rule was used for unlike non-bonded interactions. Non-bonded interactions were calculated using a 12 Å cutoff distance. Long-range electrostatic interactions were handled using fast smooth Particle-Mesh Ewald (SPME)[50] with a 0.12 nm Fourier spacing. Covalent bonds involving hydrogens were constrained using the LINCS algorithm.[51] The simulations were integrated using the leap-frog algorithm with a 1-fs timestep. Temperature coupling and pressure coupling used Nosé–Hoover thermostat with a time constant of 0.1 ps and Parrinello-Rahman barostat with a time constant of 1 ps, respectively.

Solvation Free Energy:

The Thermodynamic Integration (TI) method combined with Bennett Acceptance Ratio (BAR) method[52] was used to estimate the solvation free energy *ΔF*. Briefly, the non-bonded interactions between the solute and water was gradually turned off to transform the system from a solvated state to a reference state. Along the transformation, the coupling parameter λ was varied from 0 to 1, and the phase space was sampled at grided values of λ. Free energy was subsequently estimated by integrating the ensemble-averaged potential energy gradient along λ. In this study, the grided windows of λ were chosen so that the Coulombic interactions between the surfactant and water were gradually decoupled over the first 10 windows, and the van der Waals interactions were decoupled over the following 19 windows. At each sampling window, the starting configuration was prepared by placing a single surfactant at the center of a cubic box of length 7 nm and subsequently solvating it with water. Sodium and chloride ions were inserted to ensure charge neutrality. The starting simulation box first underwent an energy minimization to remove unphysical contacts. Then it was equilibrated under the canonical (NVT) ensemble at 300 K for 100 ps and under the isothermal-isobaric (NPT) ensemble at 300 K, 1 bar for 100 ps. The production run was subsequently carried out under the NPT ensemble at 300 K, 1 bar for 3 ns. Estimates of solvation free energies were observed to converge by 1.5 ns of production runs at each sampling window.

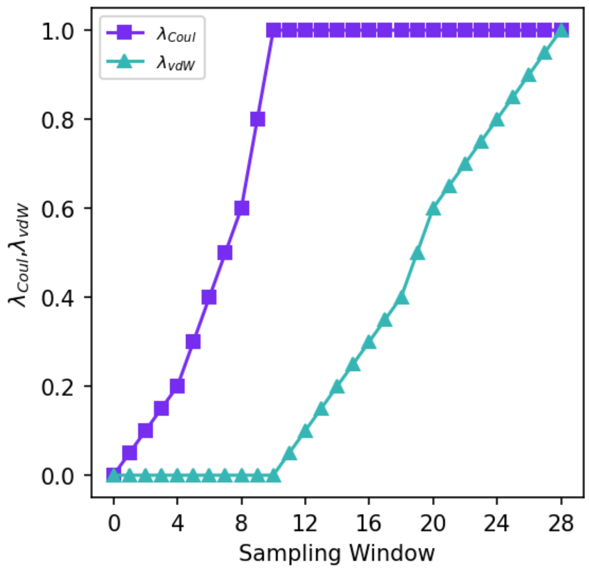

Figure S13: Values of coupling parameter for Coulombic interactions, λ_{Coul}, and van der Waals interactions λ_{vdW}. A λ of unity means the interaction is fully turned off.

Potential of Mean Force for Two-Molecule Fusion:

The PMF for two surfactant molecules to fuse was obtained using Umbrella Sampling (US). In this study, the distance between the two nitrogen atoms *d* was chosen as the reaction coordinate. The simulation box was prepared according to a similar protocol described above. Two surfactant molecules were packed in a cubic box of length 10 nm, reflecting a concentration of 0.003 mol L^{-1}. Then, six additional pairs of sodium chloride were added to the box to ensure charge neutrality and screen the long-range charge correlation. To perform the US, the two molecules were pulled apart along the reaction coordinate at a rate of 0.001 m ns^{-1} using a harmonic biasing force with a spring constant of 1000 kJ mol^{-1} nm^{-2}. Along the transition, *d* varied from 0.7 nm to 5 nm. The transition was then evenly divided into 20 sampling windows, and 20 snapshots at corresponding *d* were extracted from the pulling trajectory to use as the starting configuration for each sampling window. At each sampling window, the system first underwent a 1 ns equilibration under a harmonic potential with a spring constant of 30000 kJ mol^{-1} nm^{-2}. Then the production run was performed for 20 ns under a harmonic potential with a spring constant of 1000 kJ mol^{-1} nm^{-2}, to ensure sufficient overlap in the conformation spaces between windows. Finally, the Weighted Histogram Analysis Method (WHAM) was used to determine the PMF from the outputs of a series of US simulations.

Simulation Results:
The solvation free energy shows that both surfactants are more soluble at high pH, indicated by a much more negative *ΔF*. At each pH, **GlyC8** has a statistically lower ΔF than **AlaC8** by 8-10 kJ mol^{-1}.

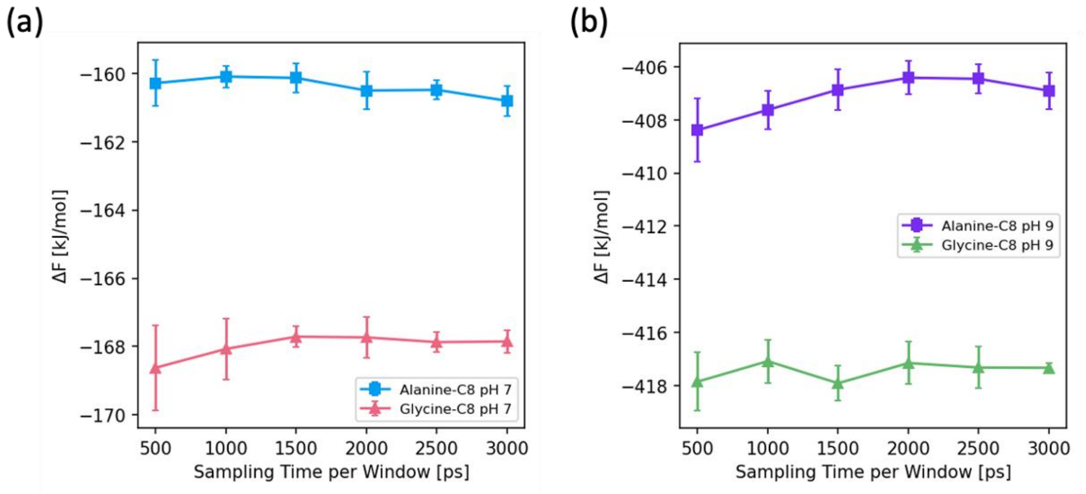

Figure S14. Solvation free energy ΔF for **AlaC8** and **GlyC8** at (a) pH = 7 and (b) pH = 9 as a function of simulation time at 300 K. 2 ns of simulation per sampling window is sufficient to produce reliable statistics, as indicated by the converging values of ΔF by 2 ns. The error bars are estimated from the BAR analysis.

Table S12 Solvation free energy ΔF in kJ mol^{-1} for **AlaC8** and **GlyC8** at pH = 7 and pH = 9 at 300 K.

	ΔF [kJ mol^{-1}]	
	pH 7	pH 9
Alanine-C8	-160.8 ± 0.4	-406.9 ± 0.7
Glycine-C8	-167.9 ± 0.3	-417.3 ± 0.2

The PMF informs us the tendency and the associated energetic barrier for two surfactant molecules to fuse together. At pH = 7, the result shows that it is not favorable for the surfactant molecules to fuse, as indicated by the rising PMF as the two molecules approach each other. At pH = 9, the free energy difference between fused and separated states is 2.33 kJ mol^{-1} for **AlaC8** and -0.48 kJ mol^{-1} for **GlyC8**. This indicates that both surfactant species tend to form or nucleate an aggregate, with **AlaC8** having a slightly stronger tendency to do so. The energy barrier associated with forming a two-molecule “aggregate” is around 5 kJ/mol for both **AlaC8** and **GlyC8**, which originates from the breaking and re-formation of the water shell surrounding the molecules.

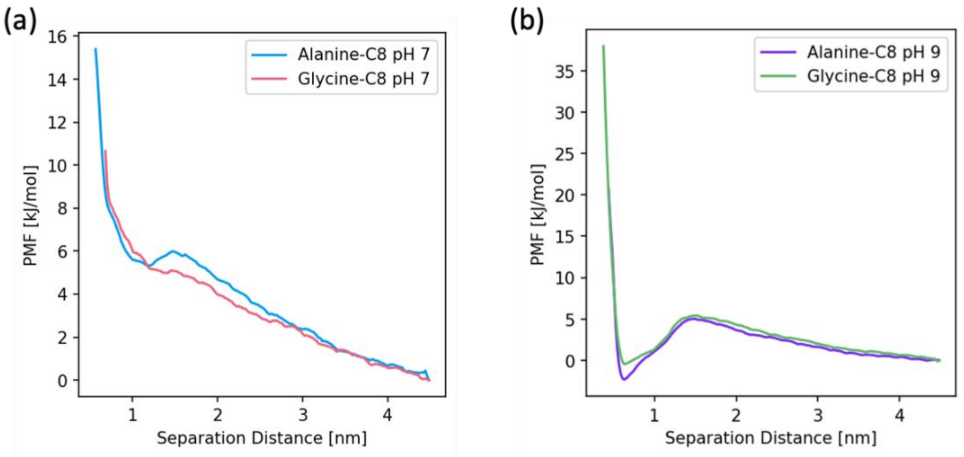

Figure S15: Potential mean force for two surfactant molecules to fuse, for **AlaC8** and **GlyC8** at (a) pH = 7 and (b) pH = 9 as a function of separation distance between their nitrogen atoms. The production runs are 20 ns long for each sampling window. The surfactant molecules are modeled as zwitterionic at pH = 7 and -1 charged at pH = 9.

Overall, simulation results suggest that the solubility trend observed in experiment is likely attributed to a few complicated factors during the aggregation process instead of the properties of individual molecules.

Simulation Visualization:

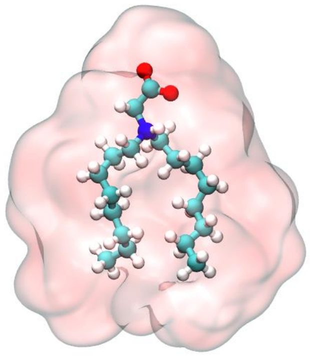

Figure S16: A snapshot of **GlyC8** at pH = 7. A diffused surface representing the surrounding water within 5 Å from the molecules is shown. Red: Oxygen. Cyan: Carbon. Blue: Nitrogen. White: Hydrogen.

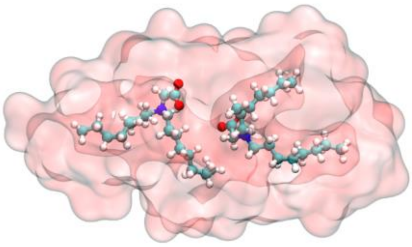

Figure S17: A snapshot of two **GlyC8** molecule at pH 7, under a harmonic biasing potential placed at d = 9 Å. A diffused surface representing the surrounding water within 5 Å from the molecules is shown. Red: Oxygen. Cyan: Carbon. Blue: Nitrogen. White: Hydrogen.

Partial Charge Assignment:

The partial charges obtained from DFT calculations were adjusted such that the charges on the two C_8 tails are symmetric.

Table S13: Partial charges assigned to different atom types. N refers to the nitrogen in the amino group; HN refers to the hydrogen attached to it. C, O1, and O2 belong to the carboxylic group. CA refers to the α-C, HCA refers to the hydrogen on α-C. CB and HCB belong to the side chain methyl group in Alanine. C1-C8 refers to the eight carbons along the C_8 tail, with C1 being closest to the amino group. HC1-HC8 refers to the corresponding hydrons attached to C1-C8, respectively.

Atom Type	Glycine-C8 pH7	Glycine-C8 pH9	Alanine-C8 pH7	Alanine-C8 pH9
N	0.096278	-0.724689	-0.07866	-0.82166
CA	-0.170613	0.131087	0.205216	0.475634
C	0.921004	0.984338	0.822205	0.874568
O1	-0.830876	-0.894567	-0.82616	-0.89217
O2	-0.840227	-0.958325	-0.81565	-0.93202
HN	0.273774	N/A	0.302767	N/A
HCA	0.081925	-0.0205015	-0.00331	-0.08714
CB	N/A	N/A	-0.25566	-0.14144
HCB	N/A	N/A	0.075446	0.00962
C1	-0.034946	0.388203	-0.03079	0.33179
C2	-0.113025	-0.171982	-0.09274	-0.20632
C3	0.088738	0.0601255	0.037786	0.20414
C4	0.028413	0.0882645	0.052597	0.000534
C5	-0.070313	-0.090781	-0.05778	0.014253
C6	0.059895	0.106294	0.049906	0.066788
C7	0.192385	0.190141	0.208314	0.211127
C8	-0.189451	-0.1877075	-0.20346	-0.19265
HC1	0.076808	-0.03685075	0.08396	-0.02033
HC2	0.047221	0.029918	0.04856	0.024435
HC3	-0.000554	-0.0062145	0.006122	-0.00526
HC4	0.00004	-0.020005	-0.00595	-0.01933
HC5	0.003799	-0.00190025	0.000815	-0.0161
HC6	-0.018244	-0.03204525	-0.01728	-0.0241
HC7	-0.044529	-0.0469555	-0.04841	-0.04764
HC8	0.034209	0.032375833	0.037326	0.032917

6. NMR spectra of new compounds

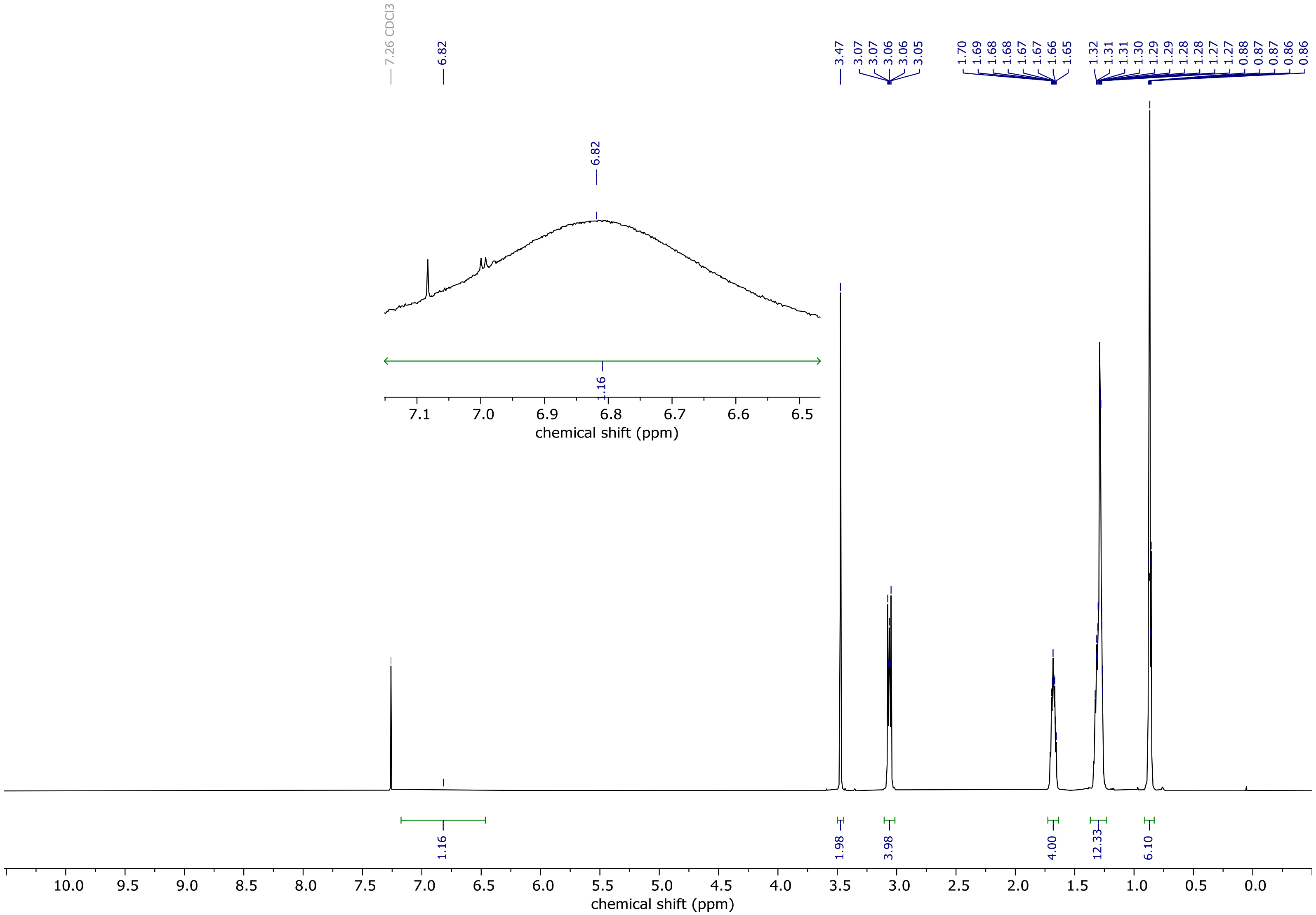

^{1}H-NMR of compound **GlyC6** (600 MHz, $CDCl_3$, 25 °C).

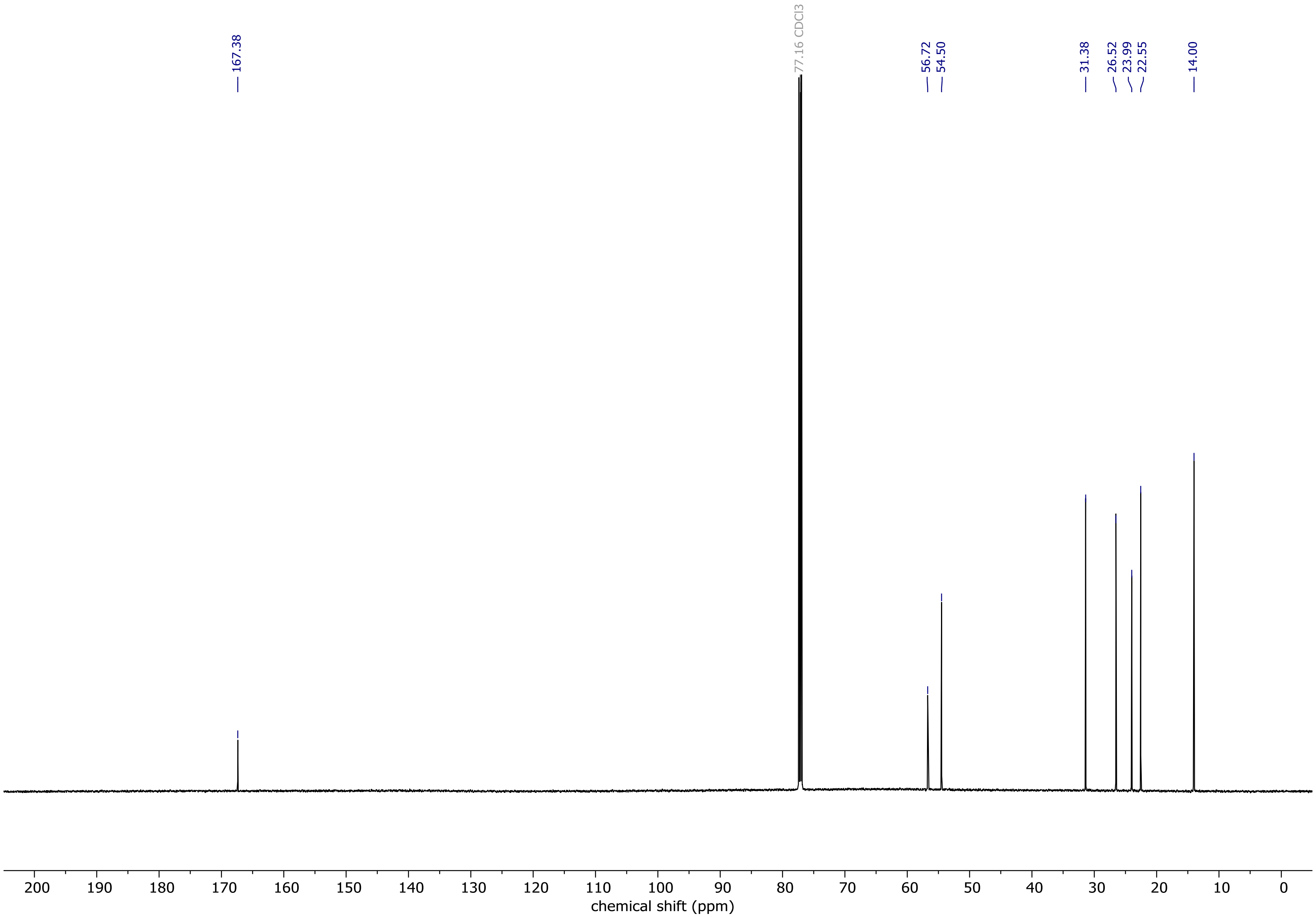

$^{13}C\{^{1}H\}$-NMR of compound **GlyC6** (151 MHz, $CDCl_3$, 25 °C).

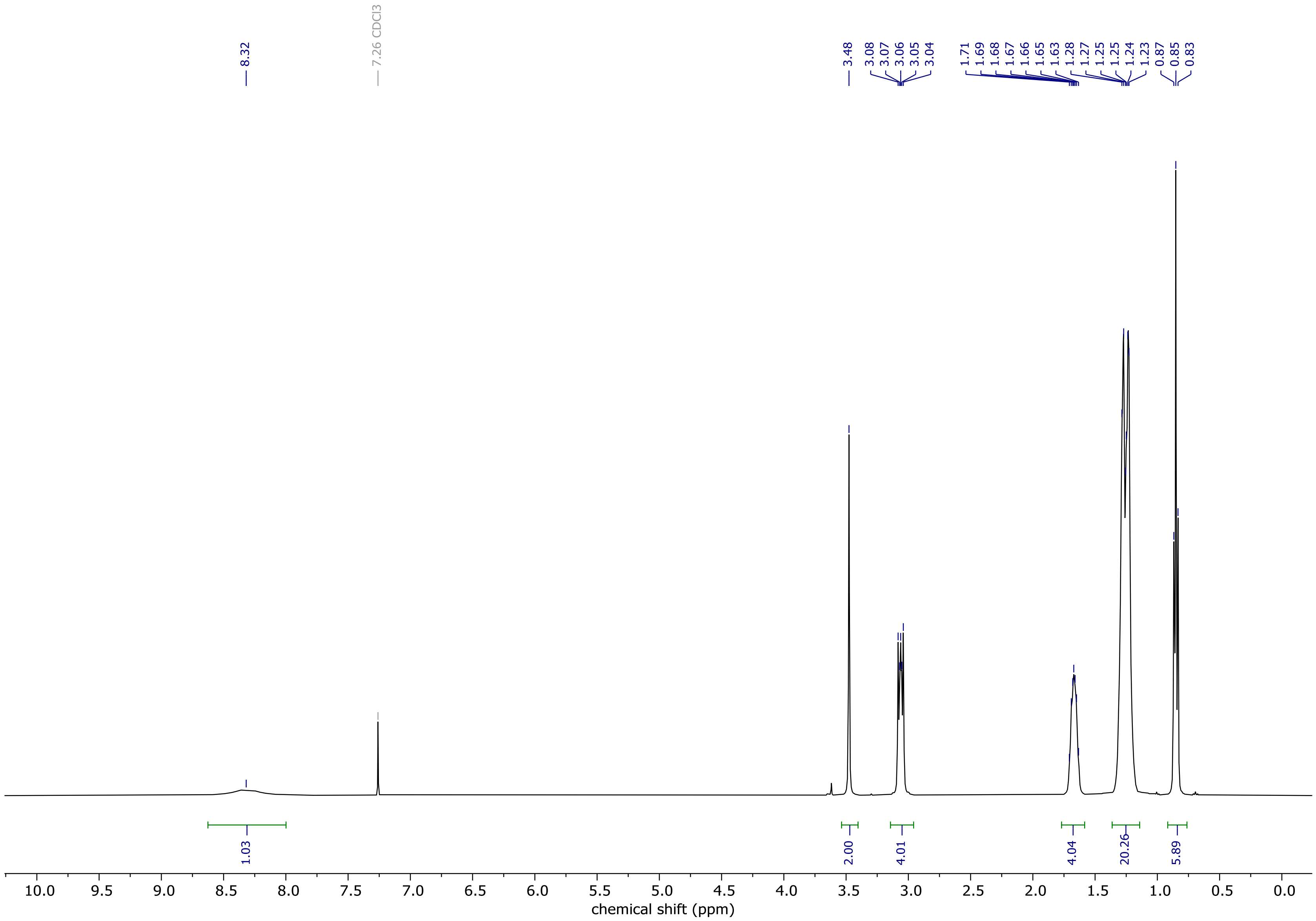

^{1}H-NMR of compound **GlyC8** (400 MHz, $CDCl_3$, 25 °C).

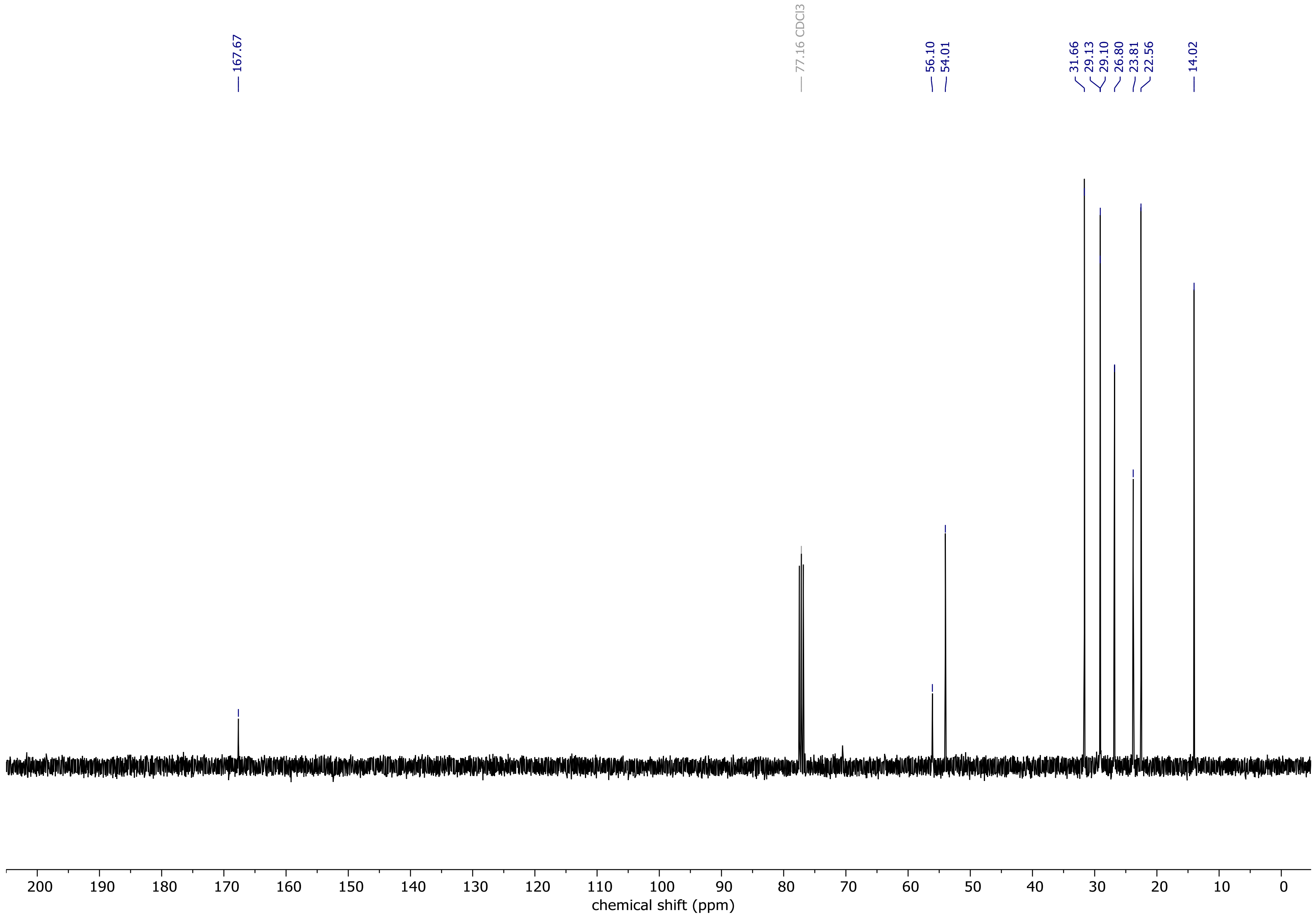

$^{13}C\{^{1}H\}$-NMR of compound **GlyC8** (101 MHz, $CDCl_3$, 25 °C).

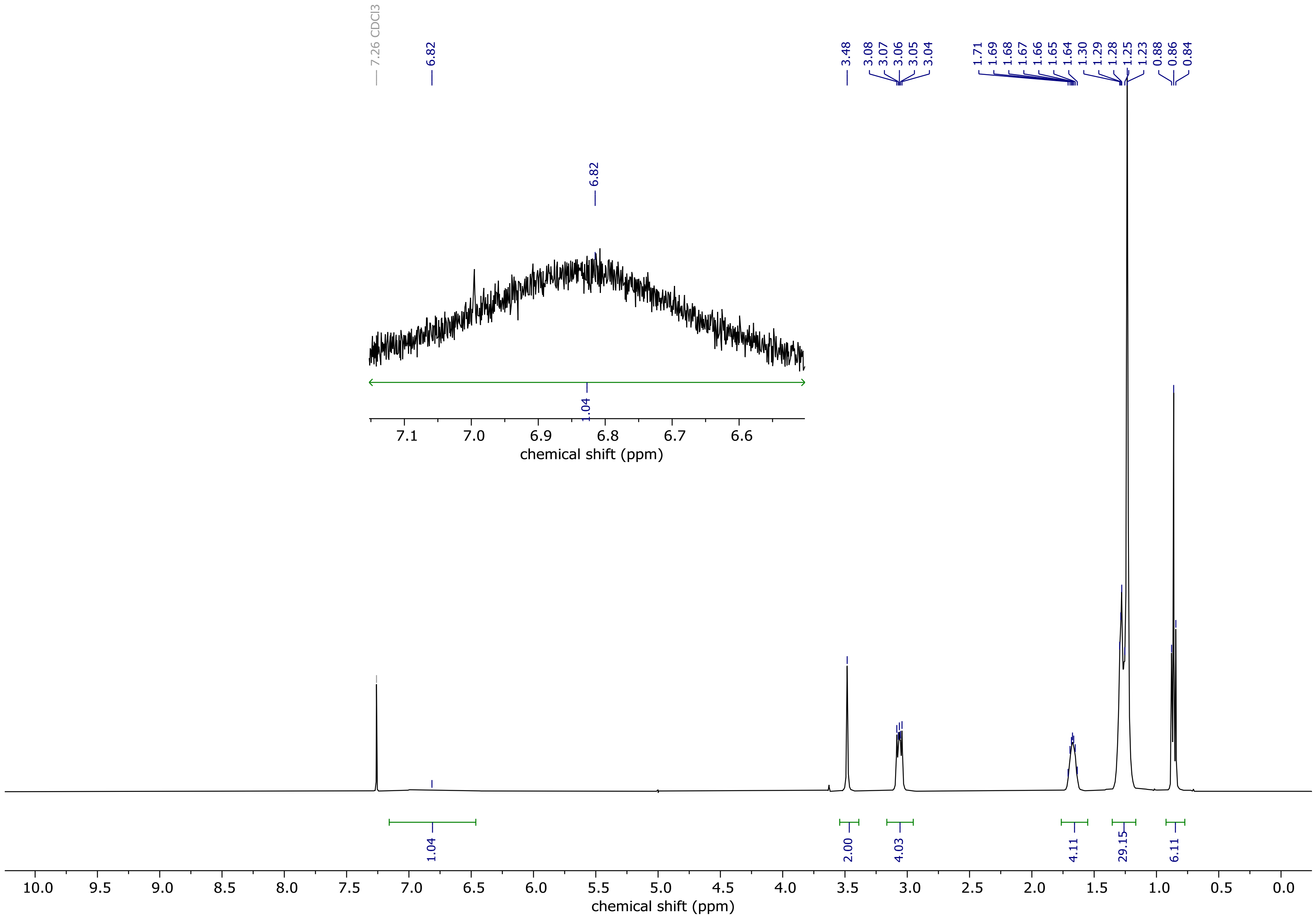

^{1}H-NMR of compound **GlyC10** (400 MHz, $CDCl_3$, 25 °C).

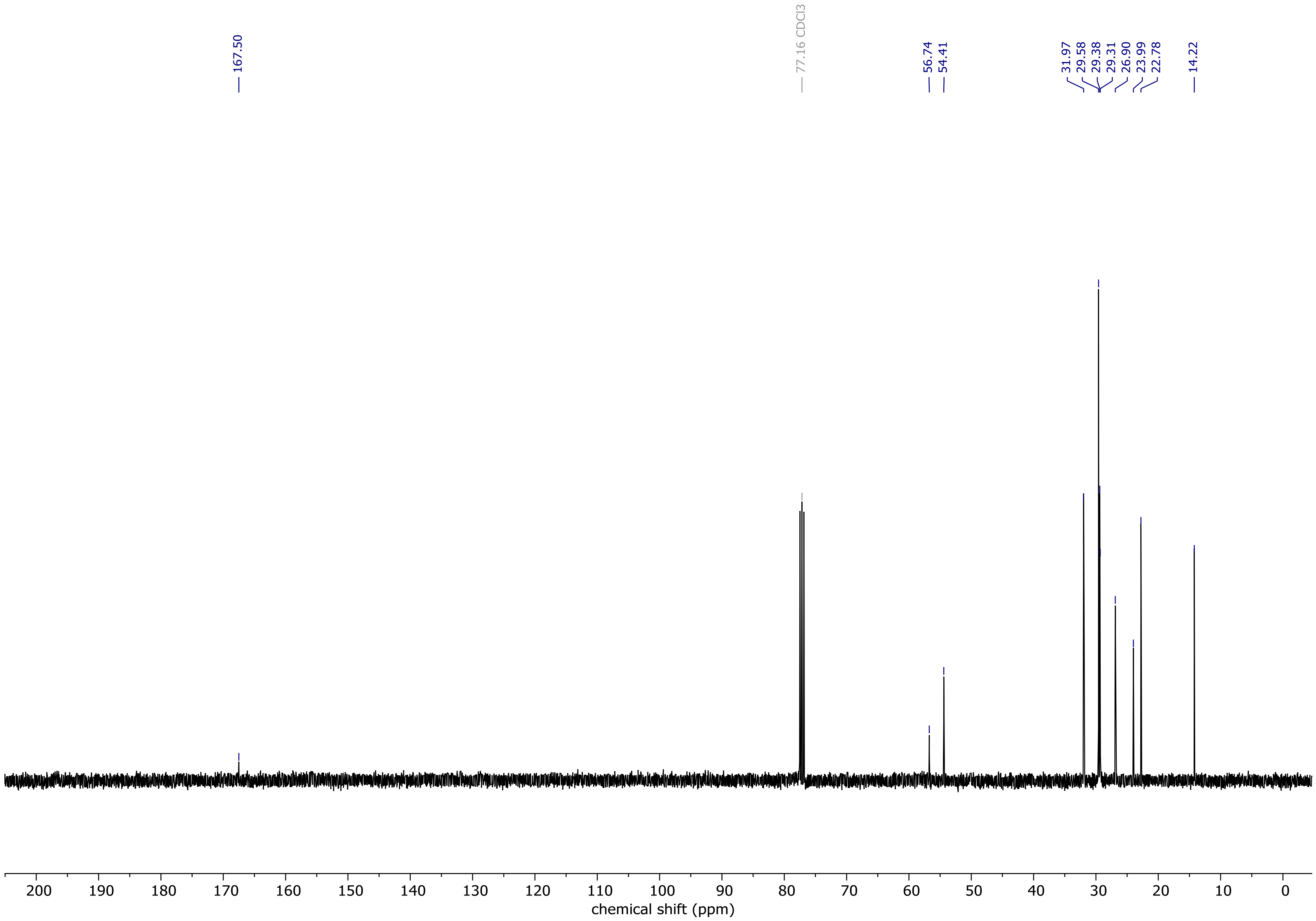

$^{13}C\{^1H\}$-NMR of compound **GlyC10** (101 MHz, $CDCl_3$, 25 °C).

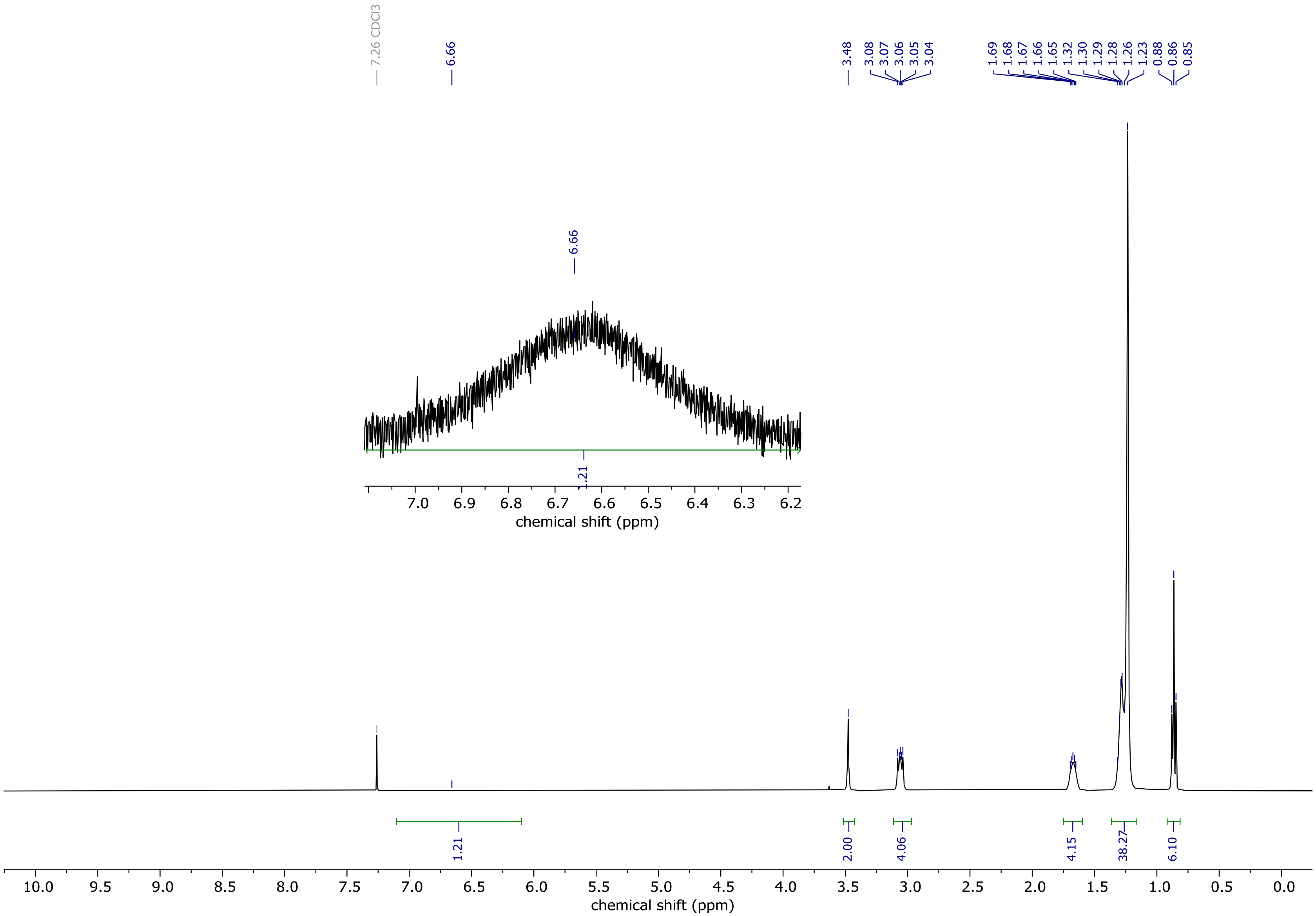

^{1}H-NMR of compound **GlyC12** (400 MHz, $CDCl_3$, 25 °C).

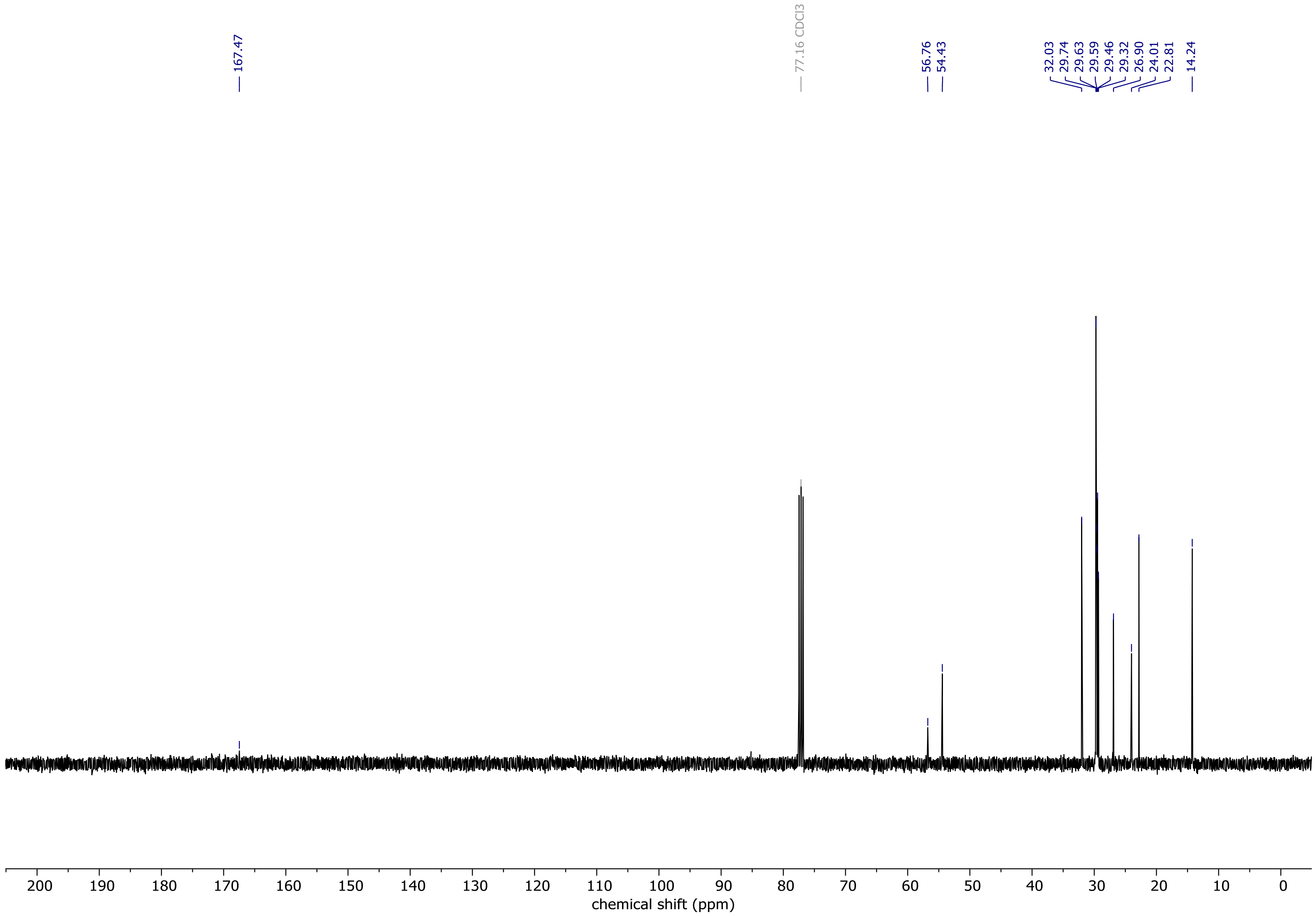

$^{13}C\{^{1}H\}$-NMR of compound **GlyC12** (101 MHz, $CDCl_3$, 25 °C).

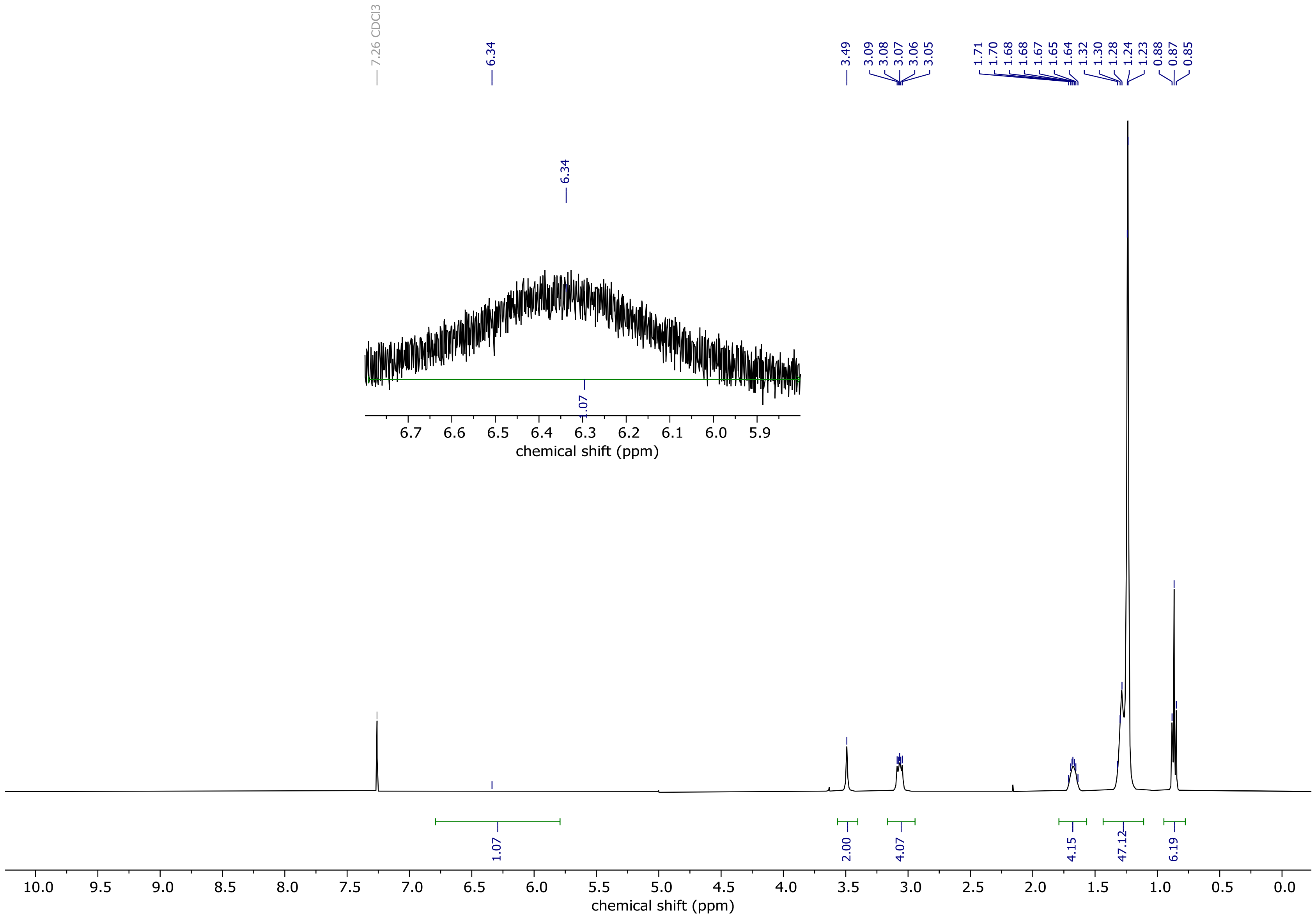

^{1}H-NMR of compound **GlyC14** (400 MHz, $CDCl_3$, 25 °C).

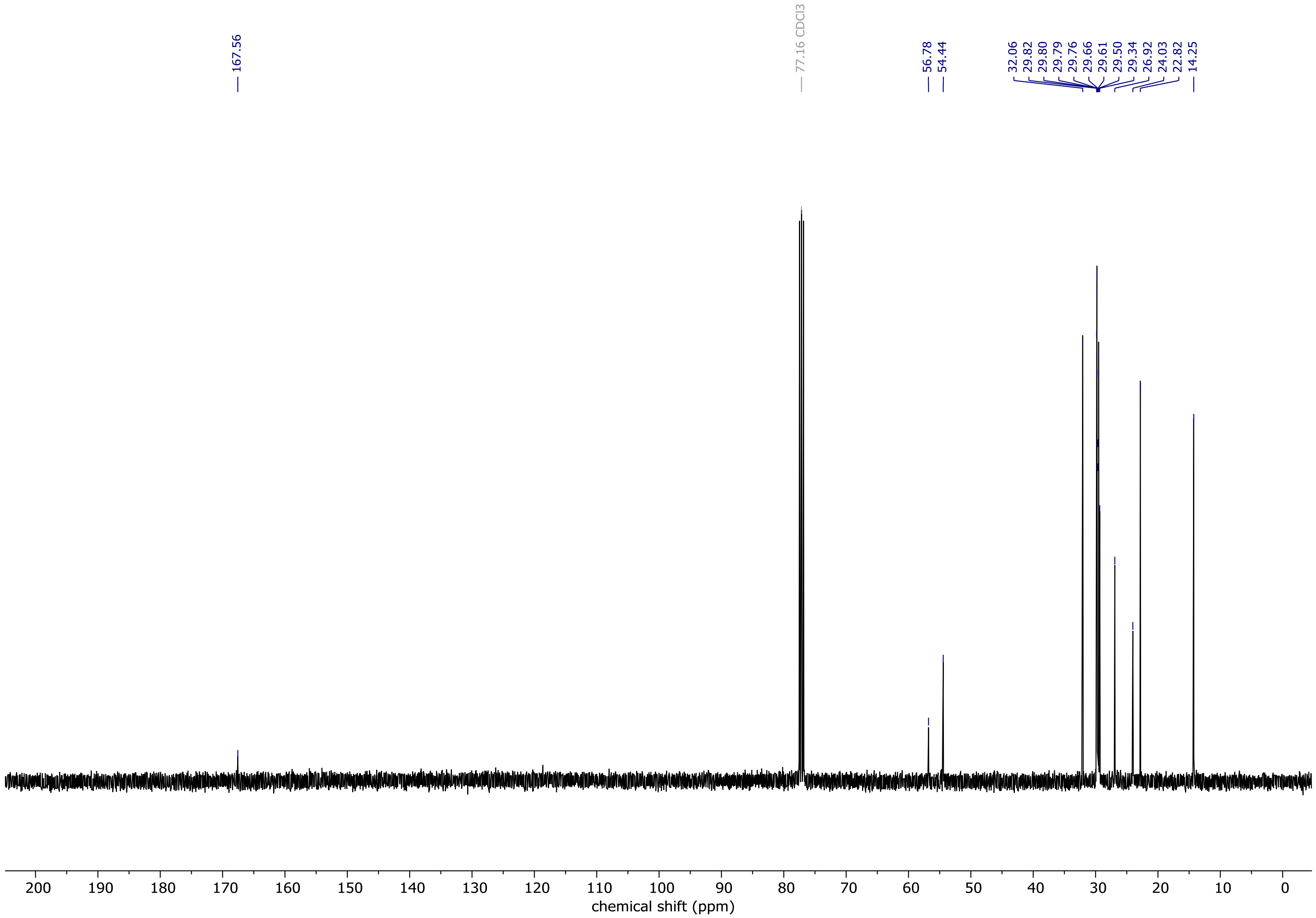

$^{13}C\{^{1}H\}$-NMR of compound **GlyC14** (101 MHz, $CDCl_3$, 25 °C).

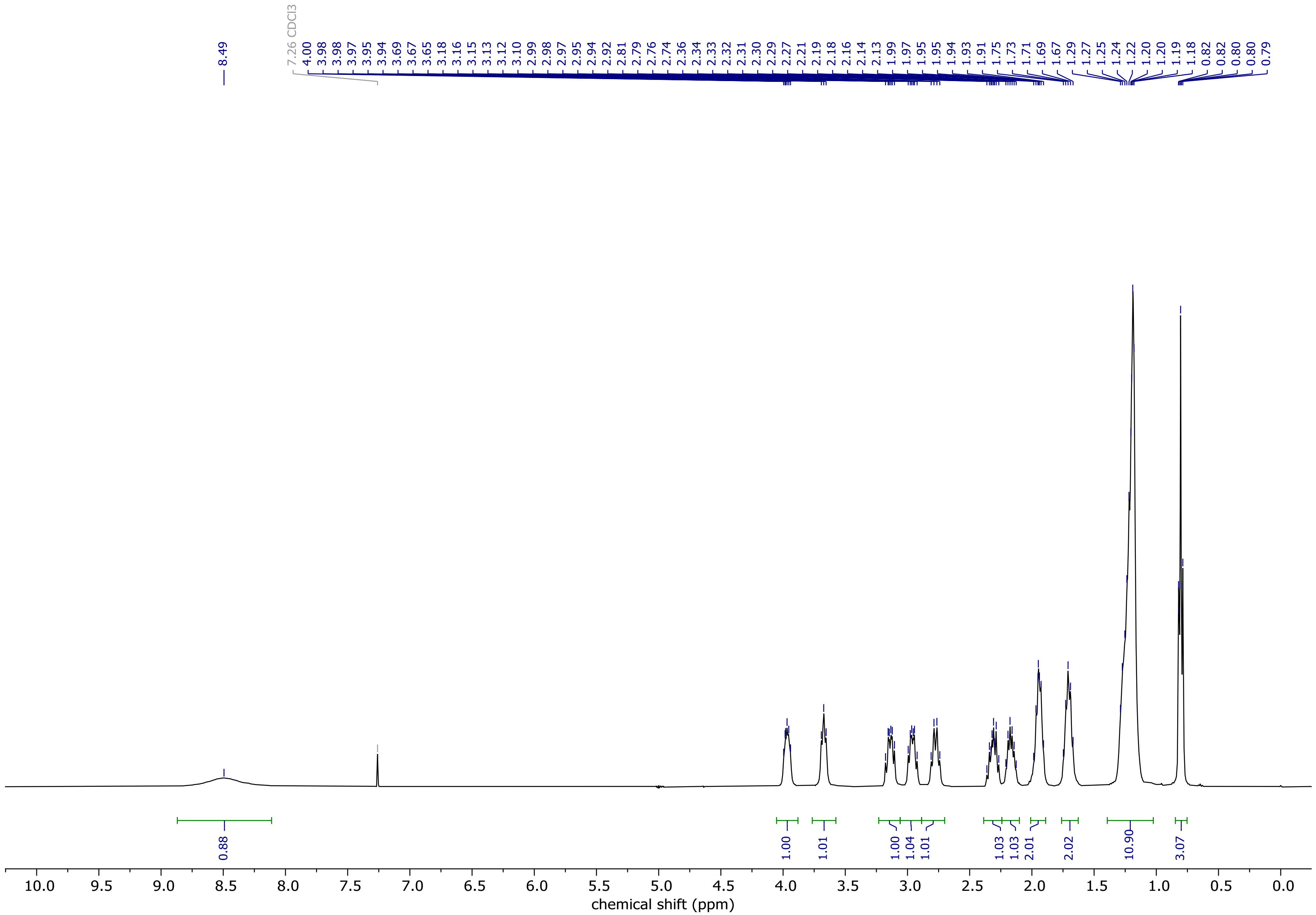

^{1}H-NMR of compound **ProC8** (400 MHz, $CDCl_3$, 25 °C).

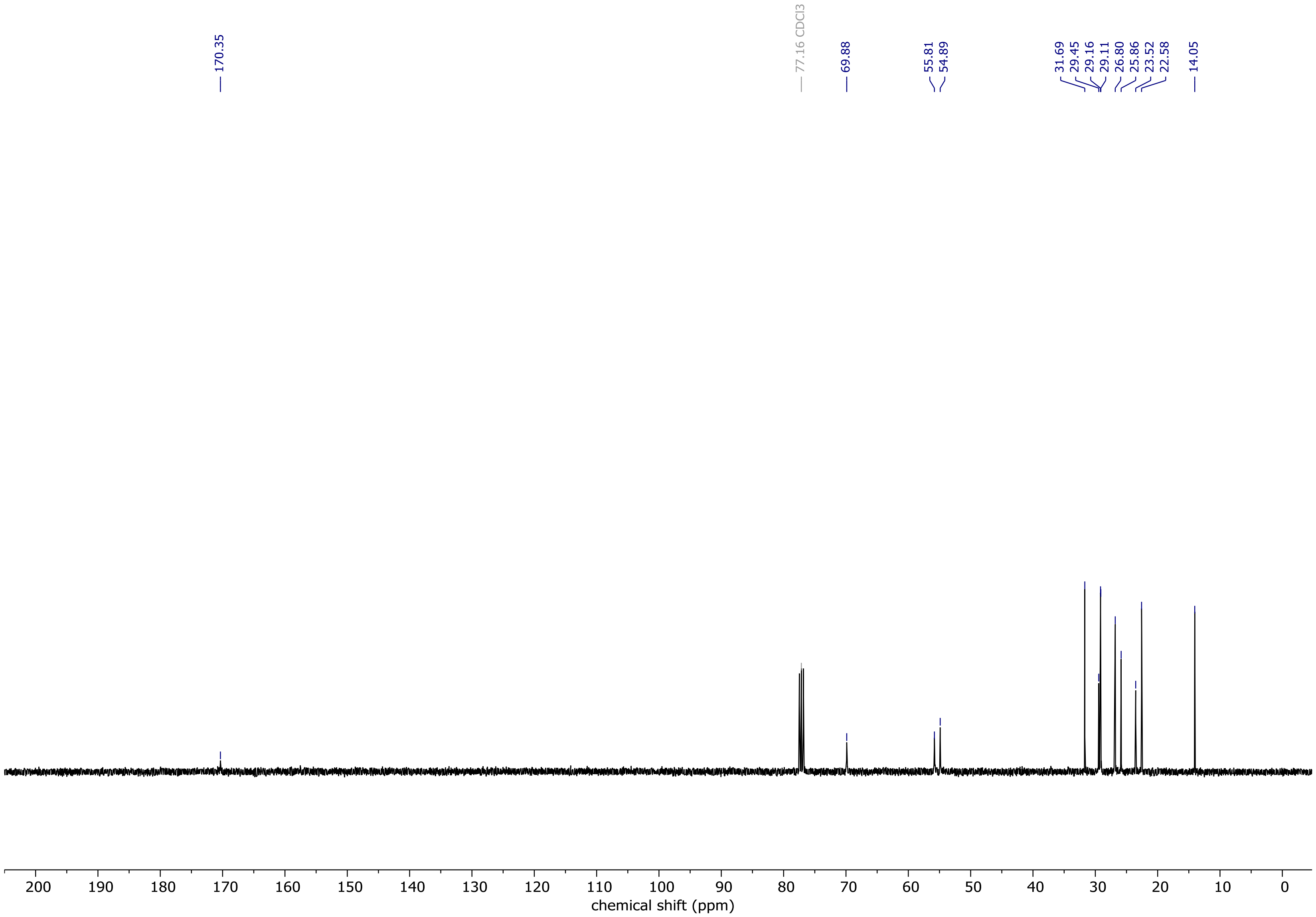

$^{13}C\{^1H\}$-NMR of compound **ProC8** (101 MHz, $CDCl_3$, 25 °C).

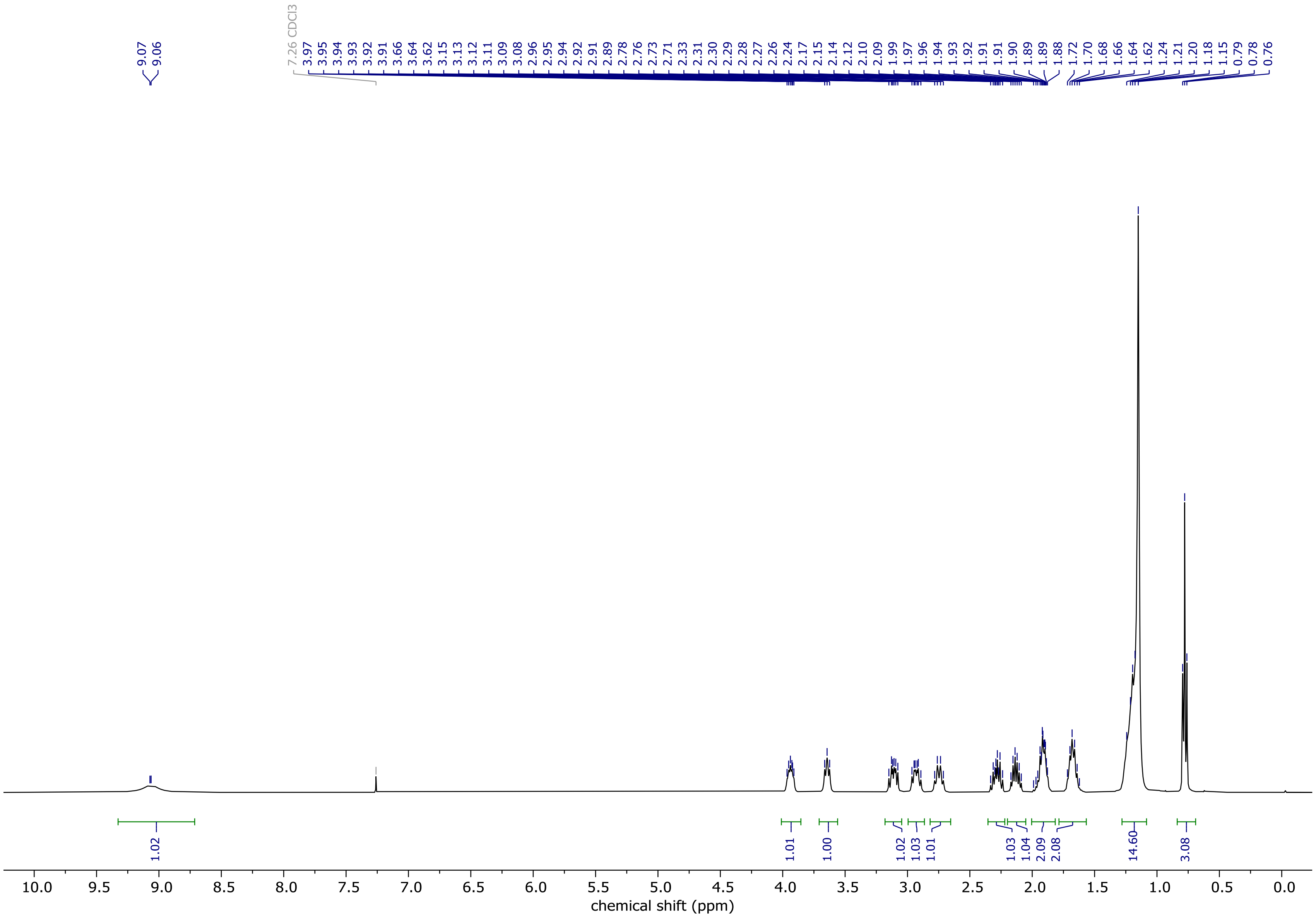

^{1}H-NMR of compound **ProC10** (400 MHz, $CDCl_3$, 25 °C).

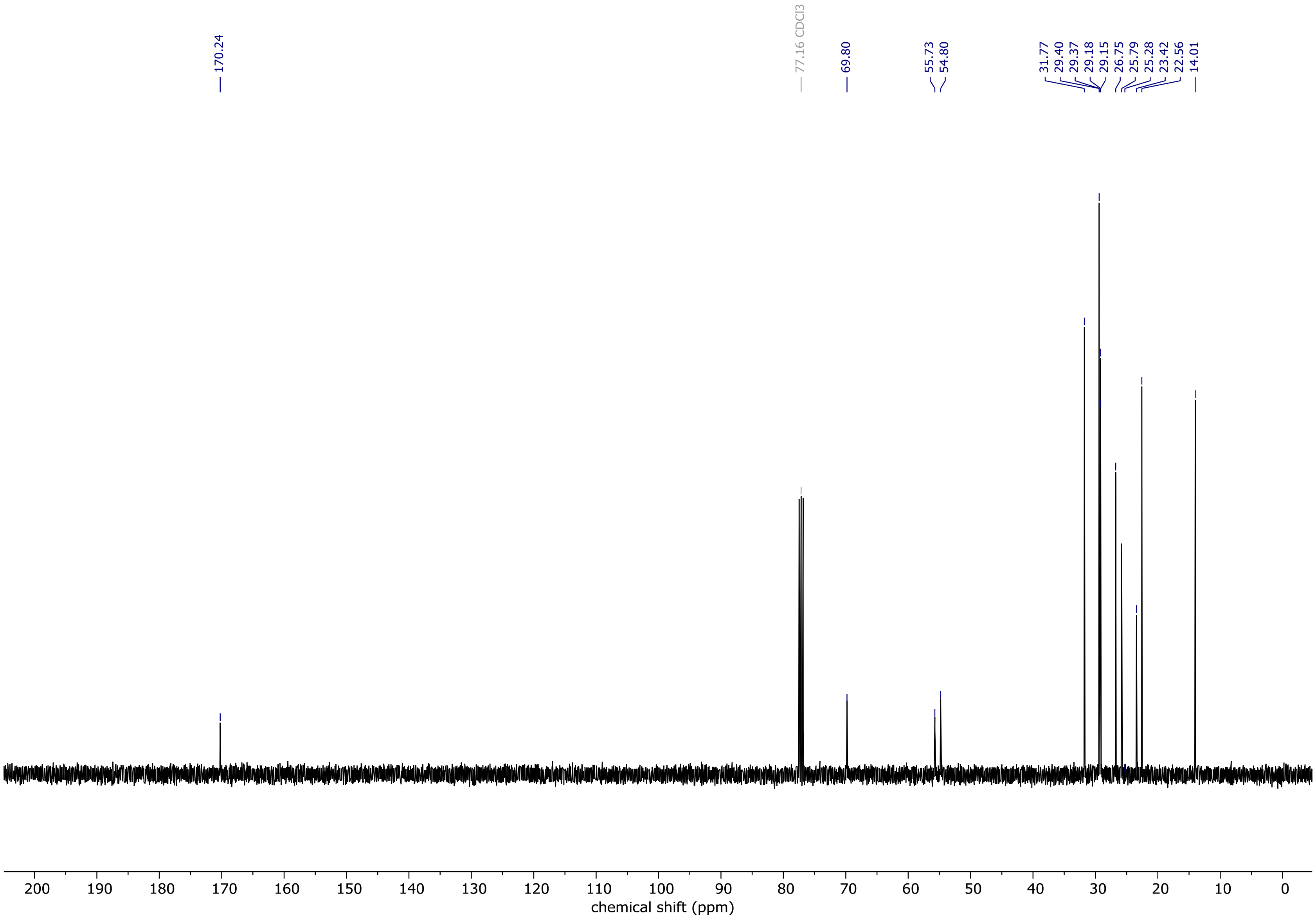

$^{13}C\{^{1}H\}$-NMR of compound **ProC10** (101 MHz, $CDCl_3$, 25 °C).

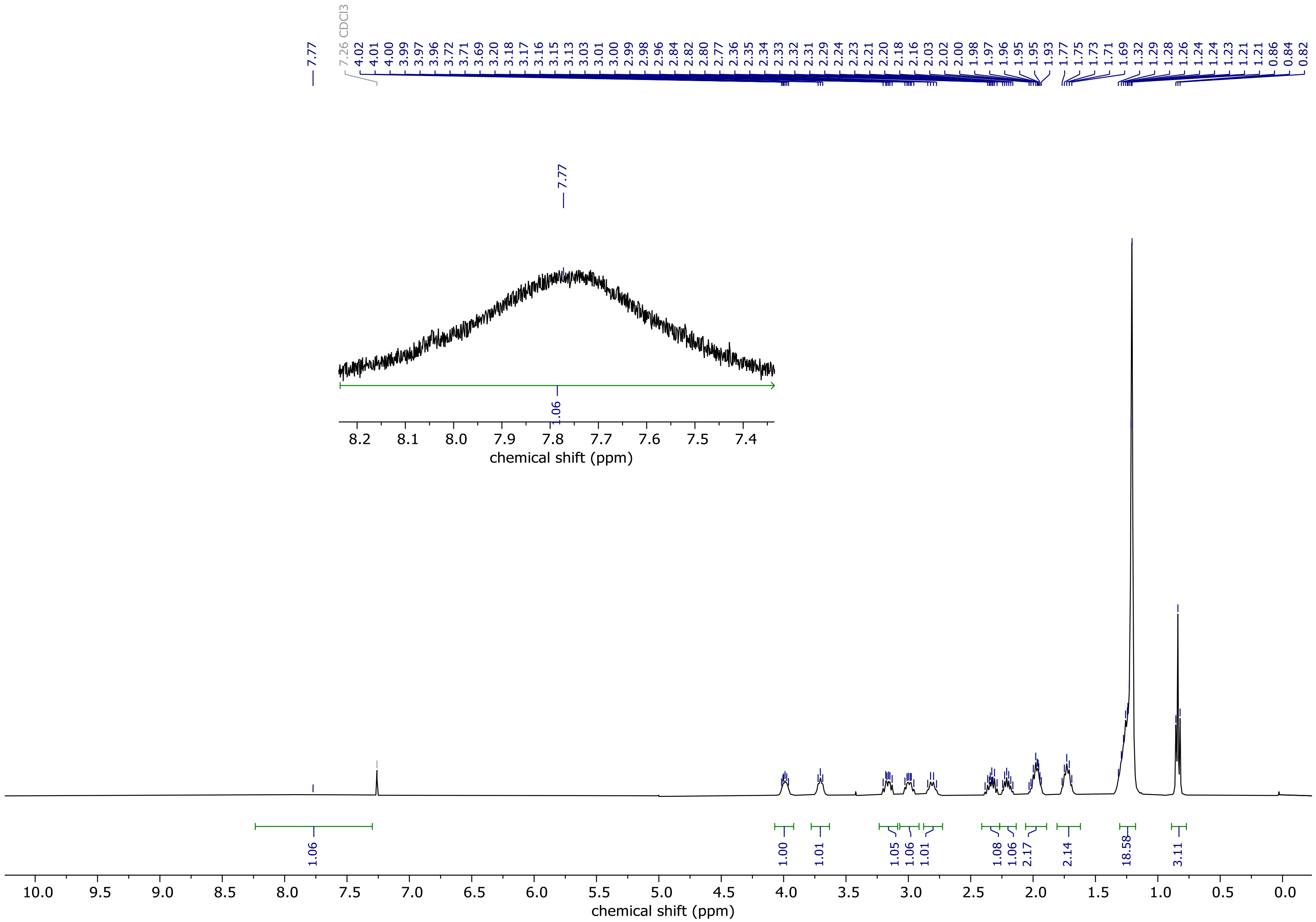

^{1}H-NMR of compound **ProC12** (400 MHz, $CDCl_3$, 25 °C).

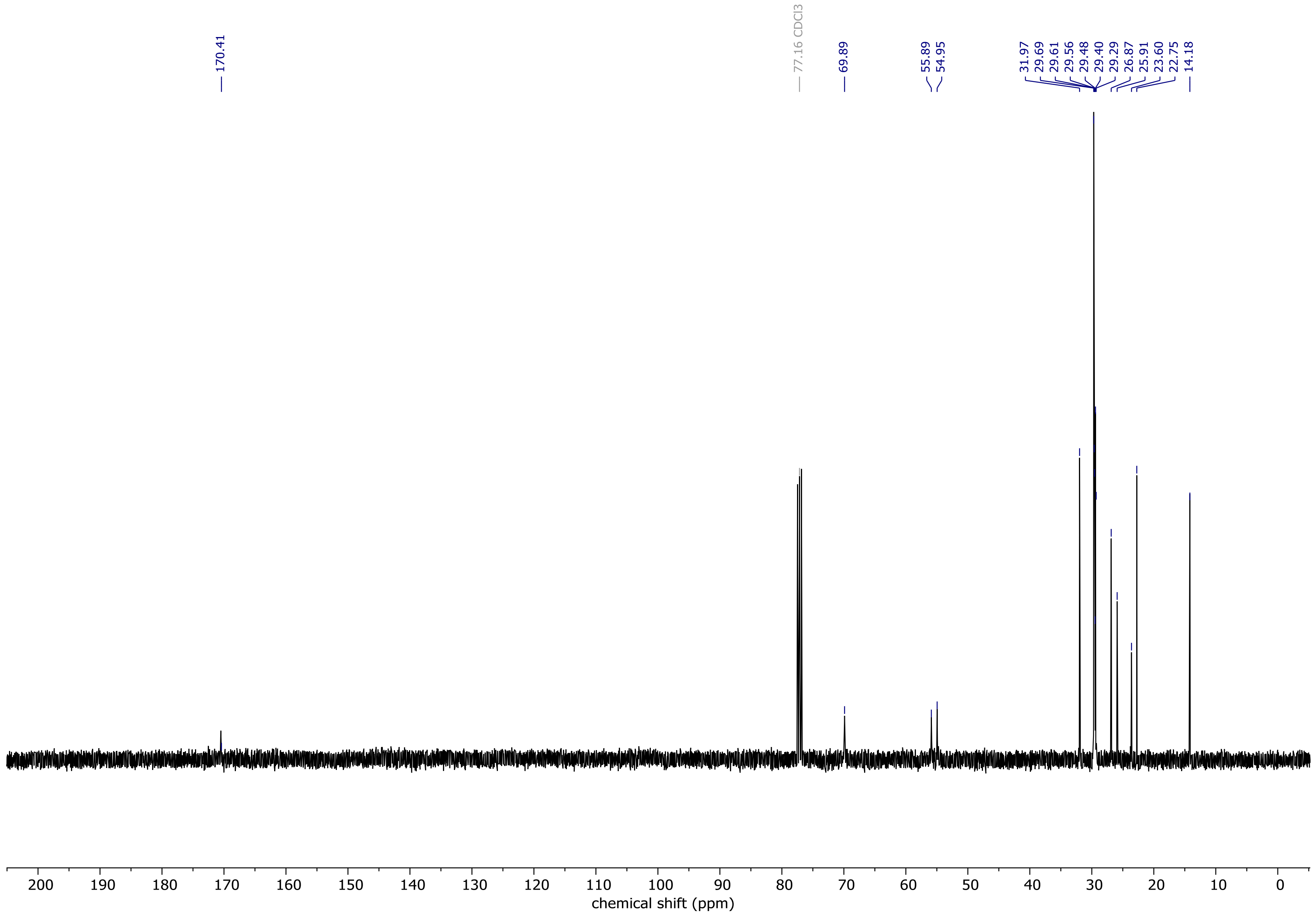

$^{13}C\{^{1}H\}$-NMR of compound **ProC12** (101 MHz, $CDCl_3$, 25 °C).

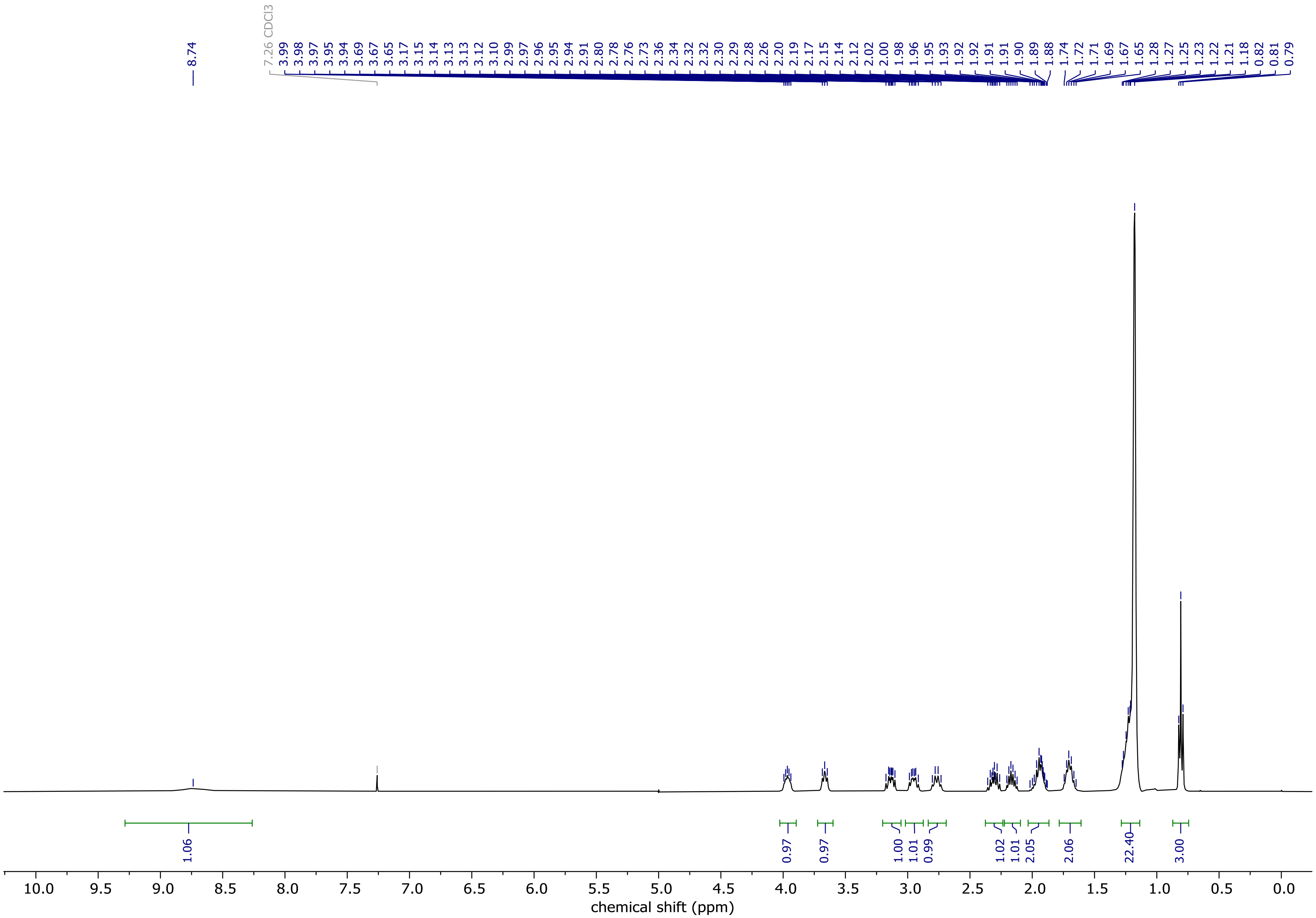

^{1}H-NMR of compound **ProC14** (400 MHz, $CDCl_3$, 25 °C).

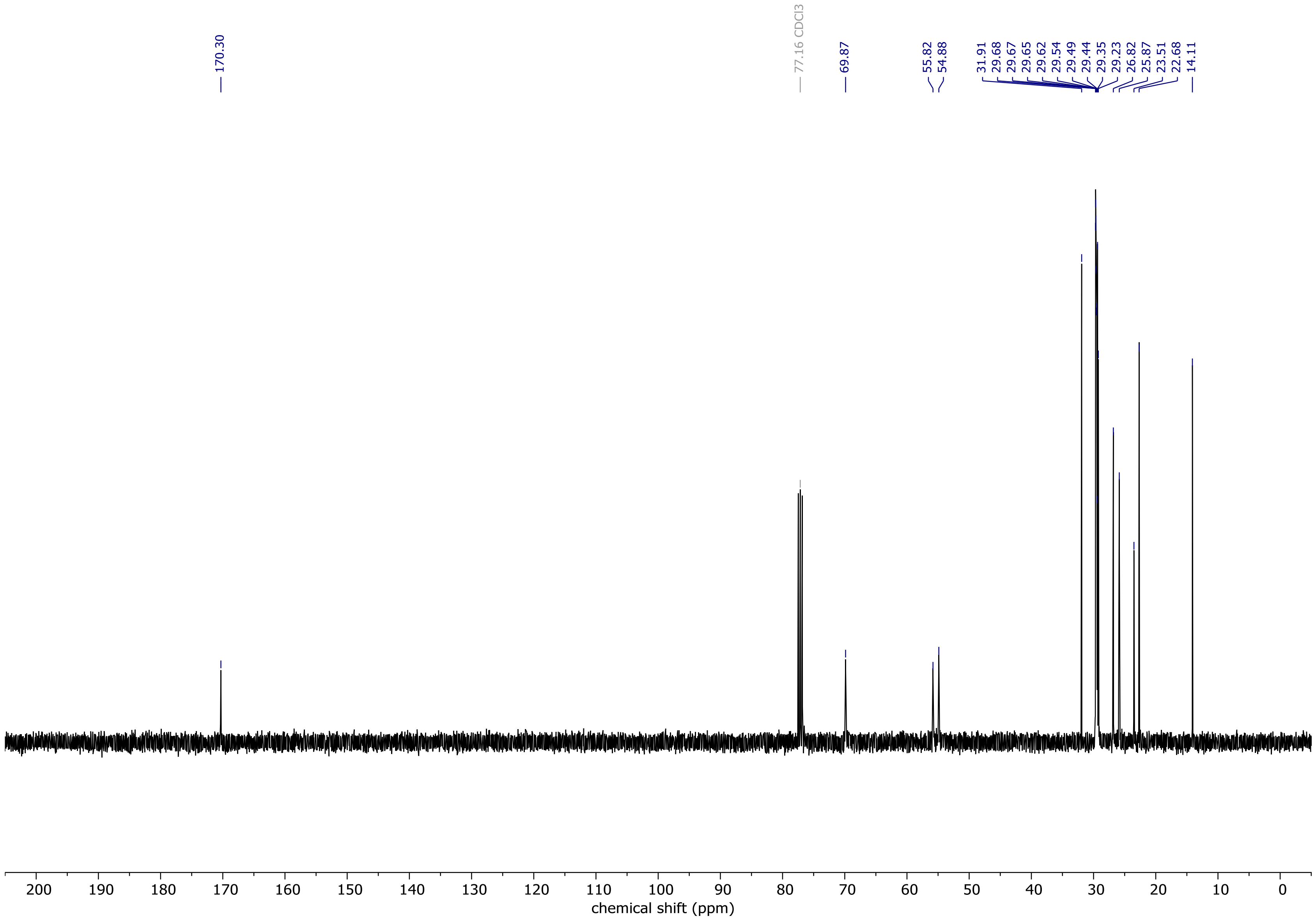

$^{13}C\{^{1}H\}$-NMR of compound **ProC14** (101 MHz, $CDCl_3$, 25 °C).

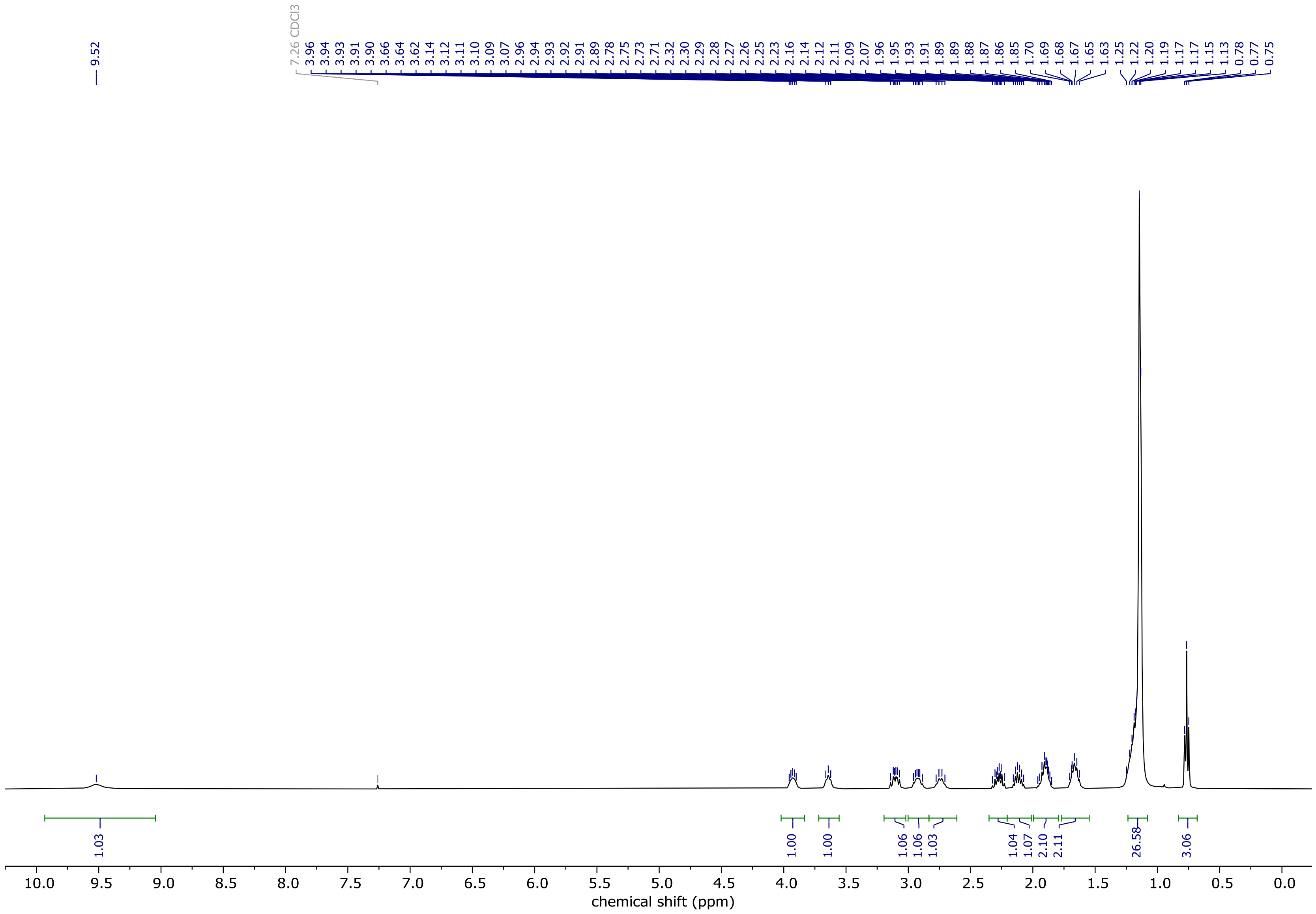

^{1}H-NMR of compound **ProC16** (400 MHz, $CDCl_3$, 25 °C).

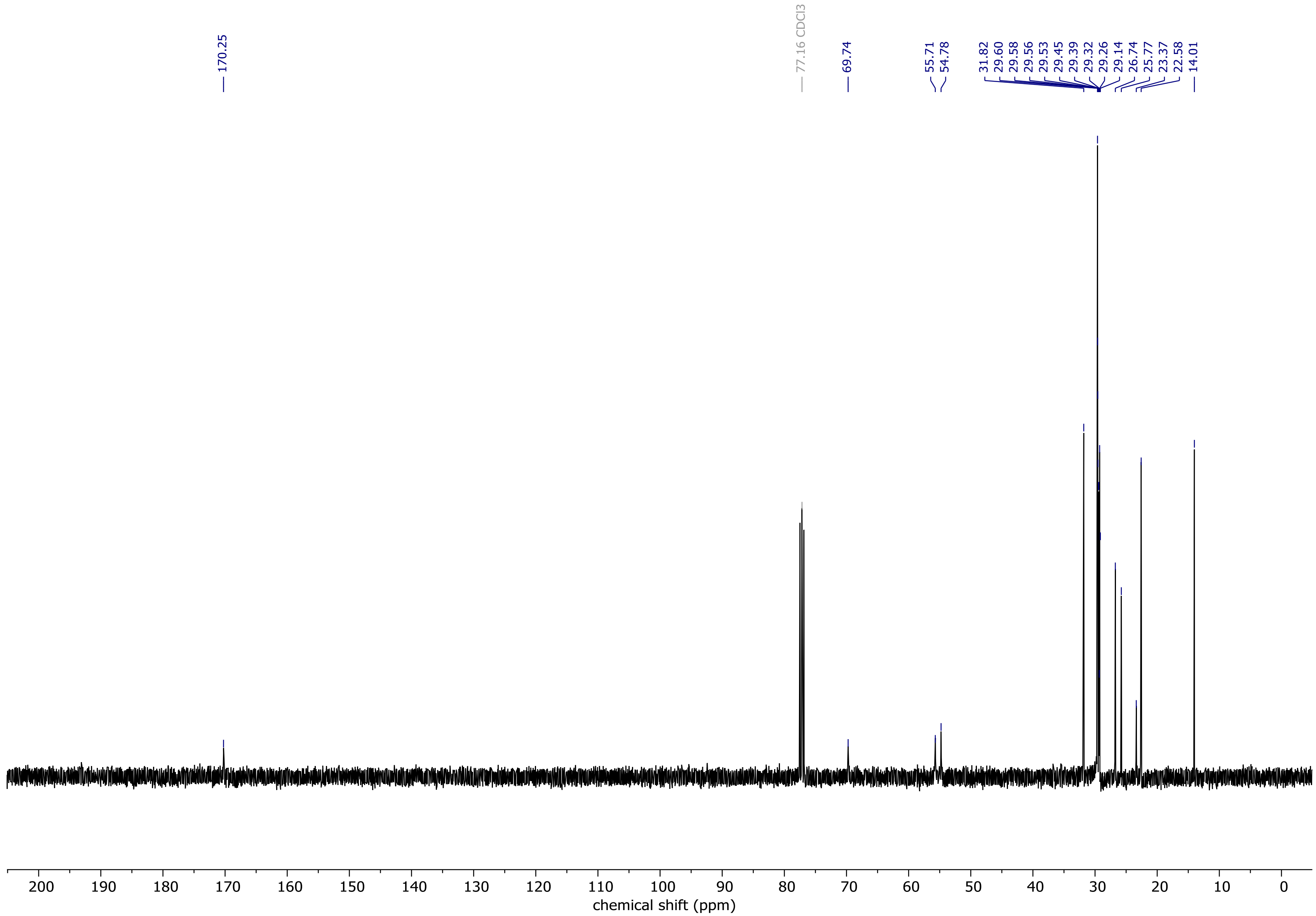

$^{13}C\{^{1}H\}$-NMR of compound **ProC16** (101 MHz, $CDCl_3$, 25 °C).

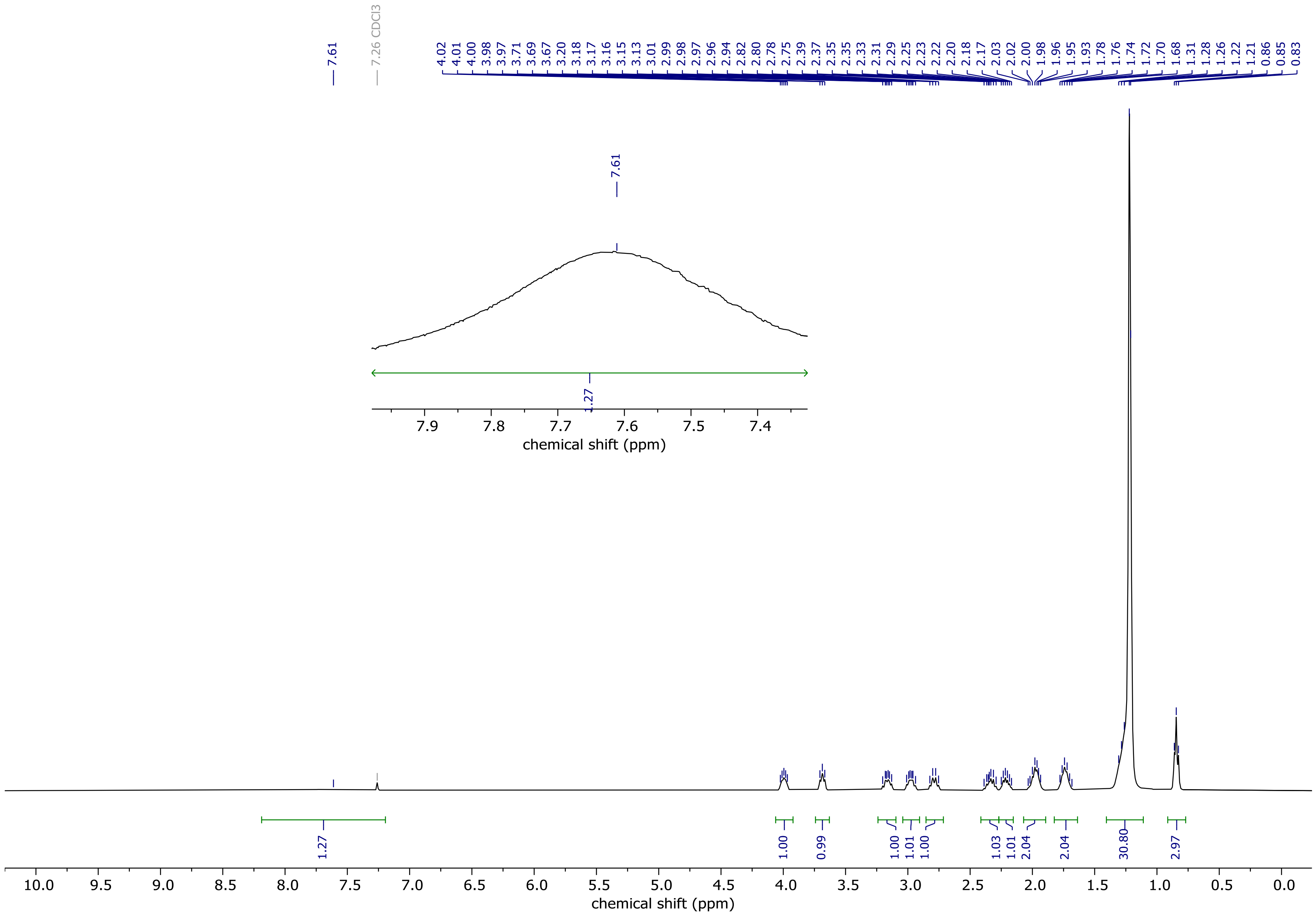

^{1}H-NMR of compound **ProC18** (400 MHz, $CDCl_3$, 25 °C).

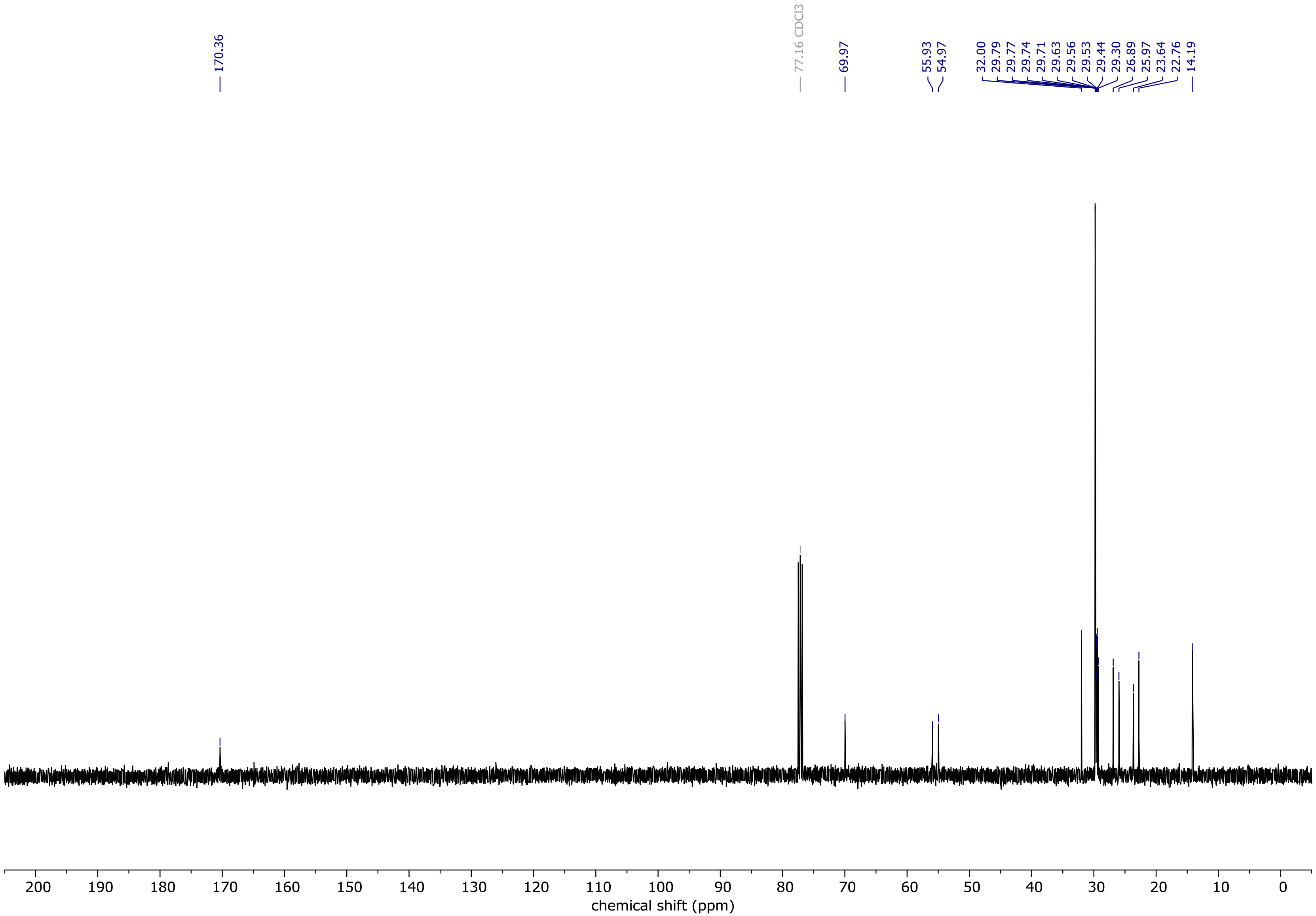

$^{13}C\{^1H\}$-NMR of compound **ProC18** (101 MHz, $CDCl_3$, 25 °C).

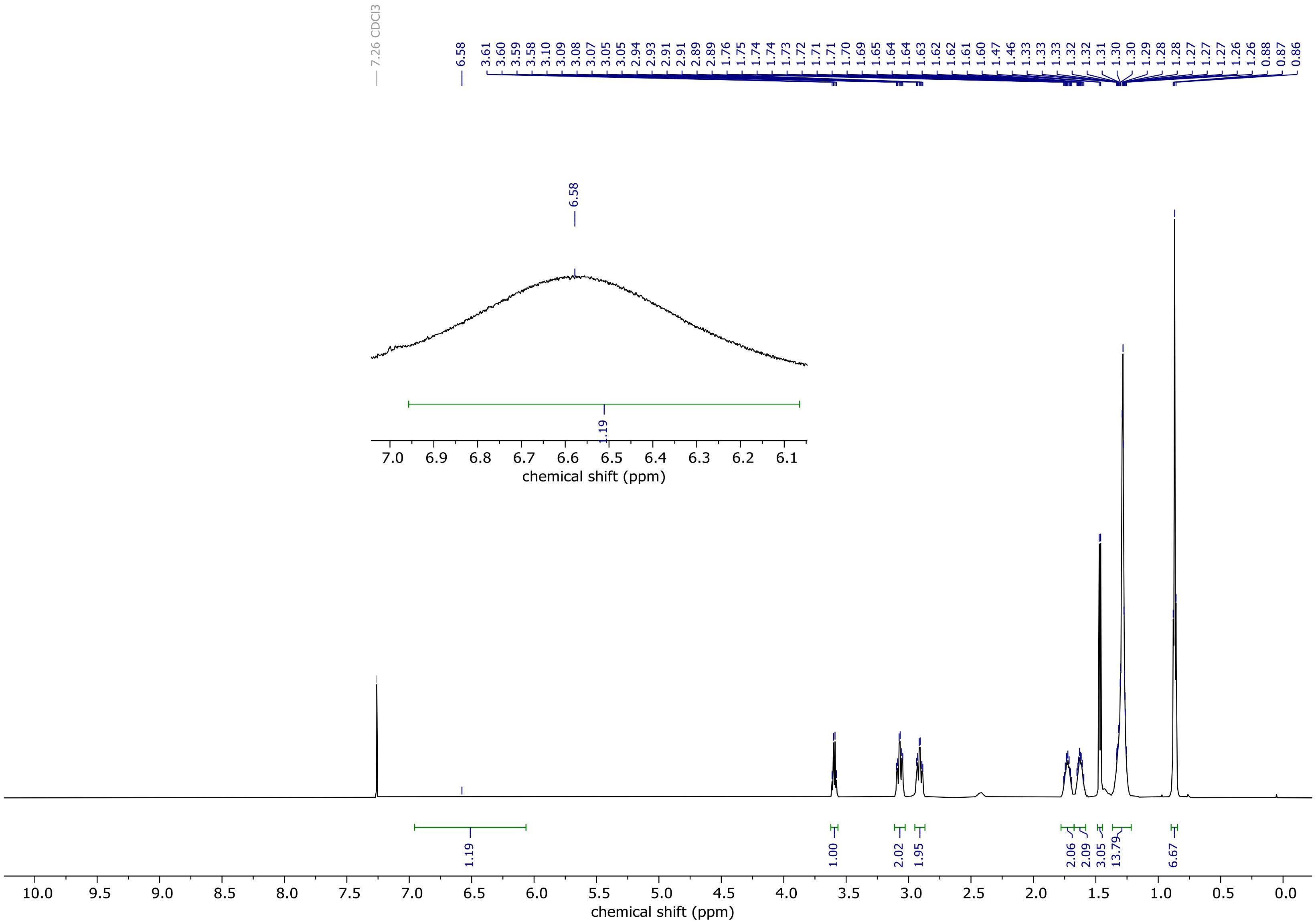

^{1}H-NMR of compound **AlaC6** (600 MHz, $CDCl_3$, 25 °C).

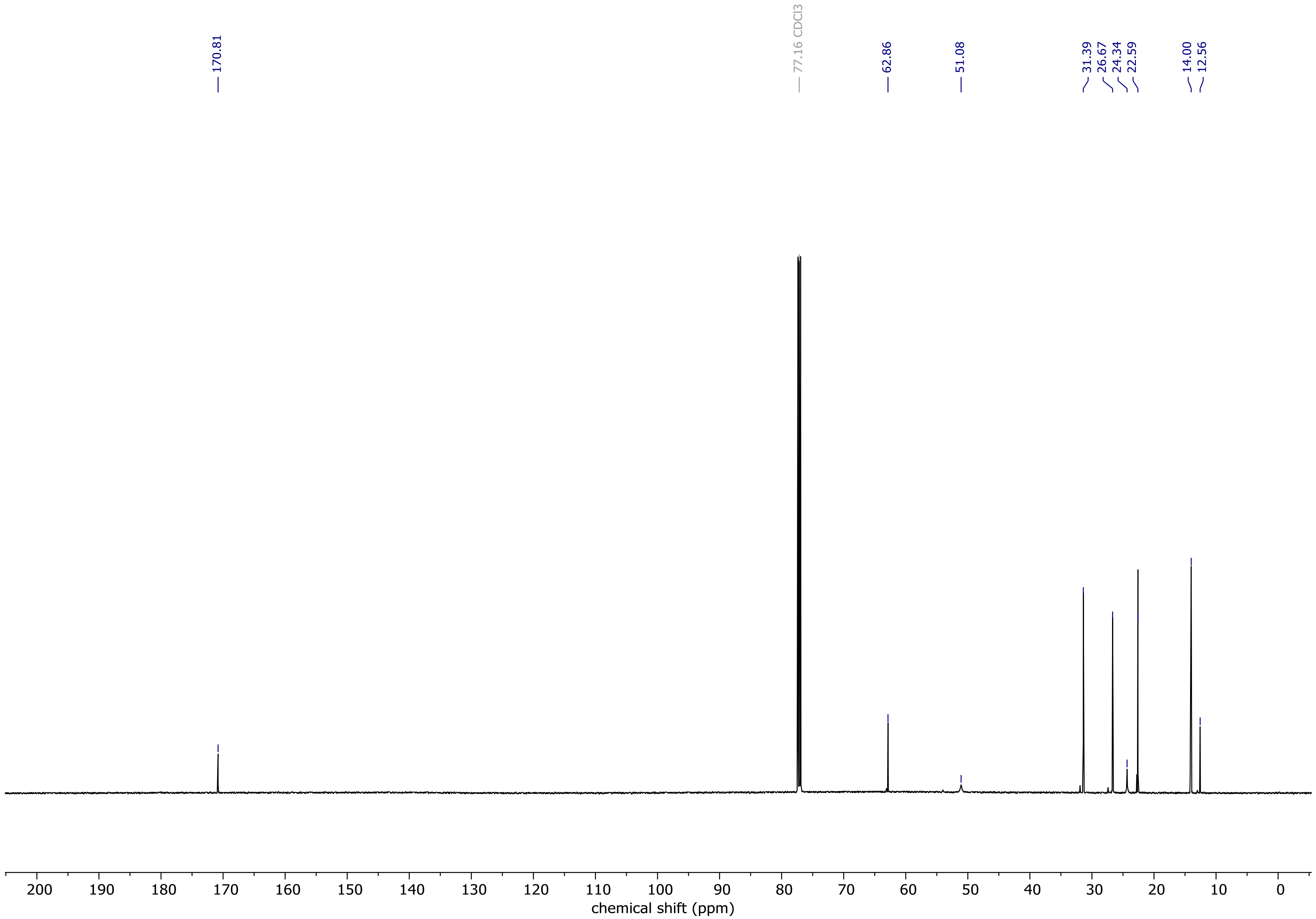

$^{13}C\{^{1}H\}$-NMR of compound **AlaC6** (151 MHz, $CDCl_3$, 25 °C).

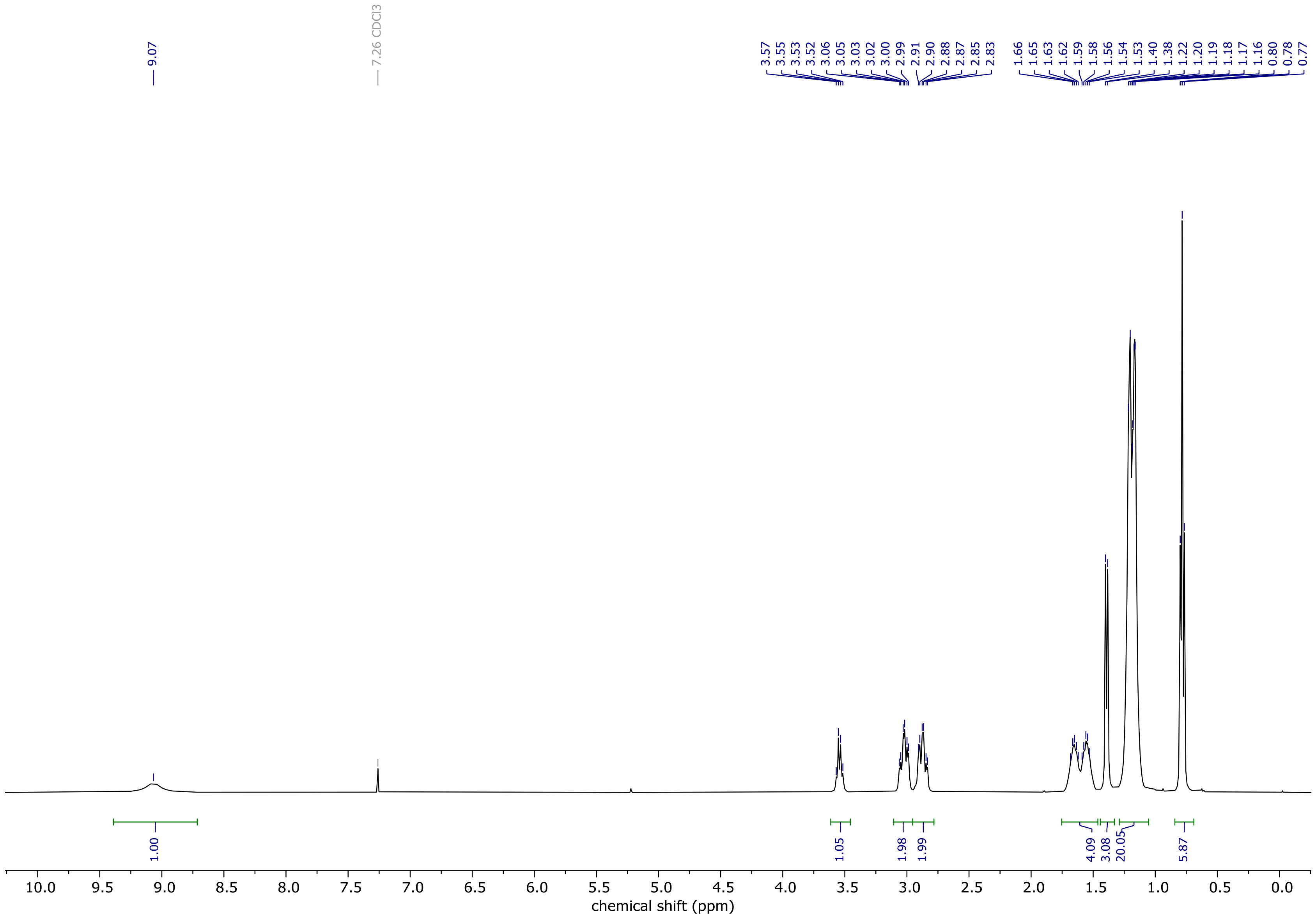

^{1}H-NMR of compound **AlaC8** (400 MHz, $CDCl_3$, 25 °C).

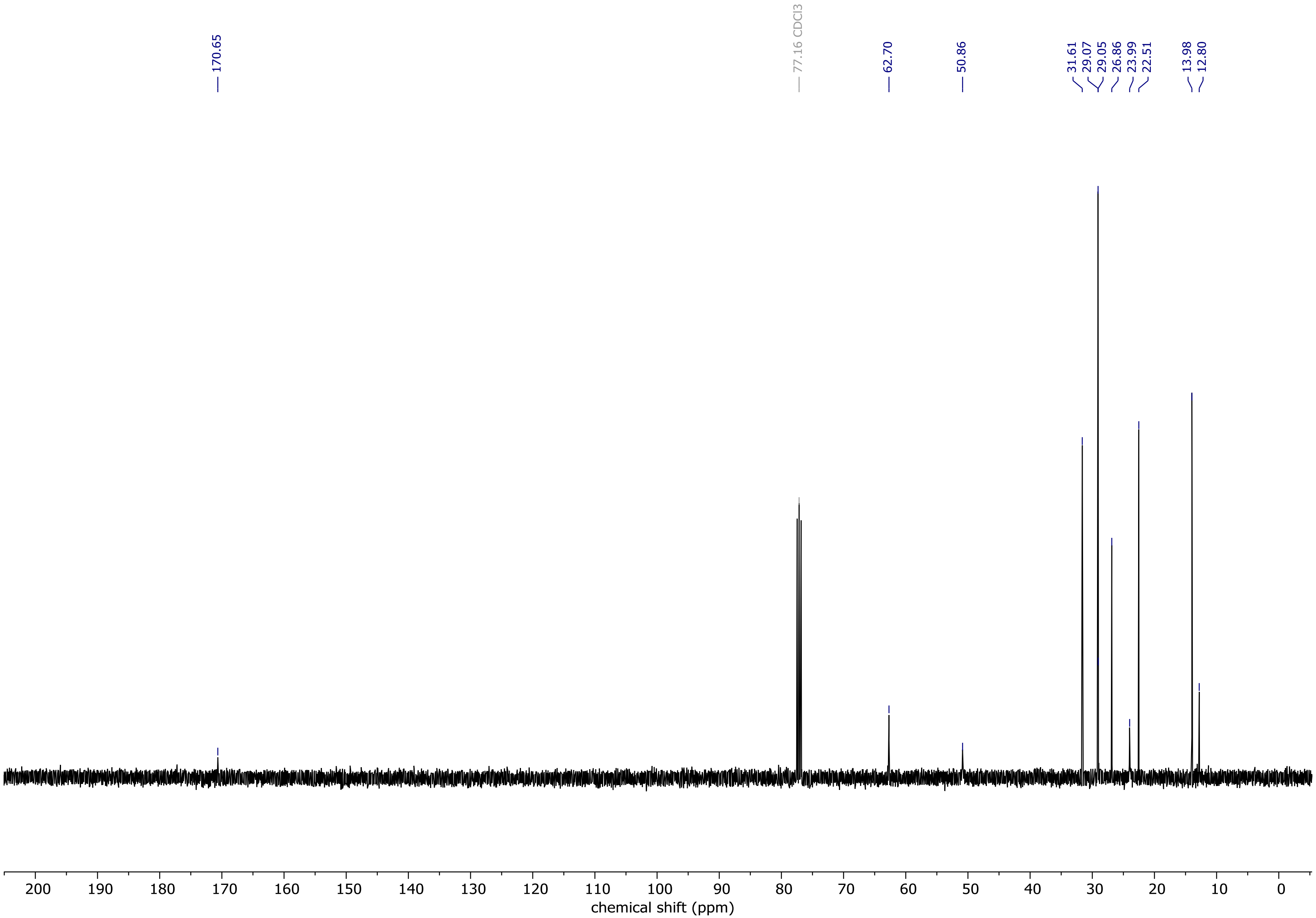

$^{13}C\{^{1}H\}$-NMR of compound **AlaC8** (101 MHz, $CDCl_3$, 25 °C).

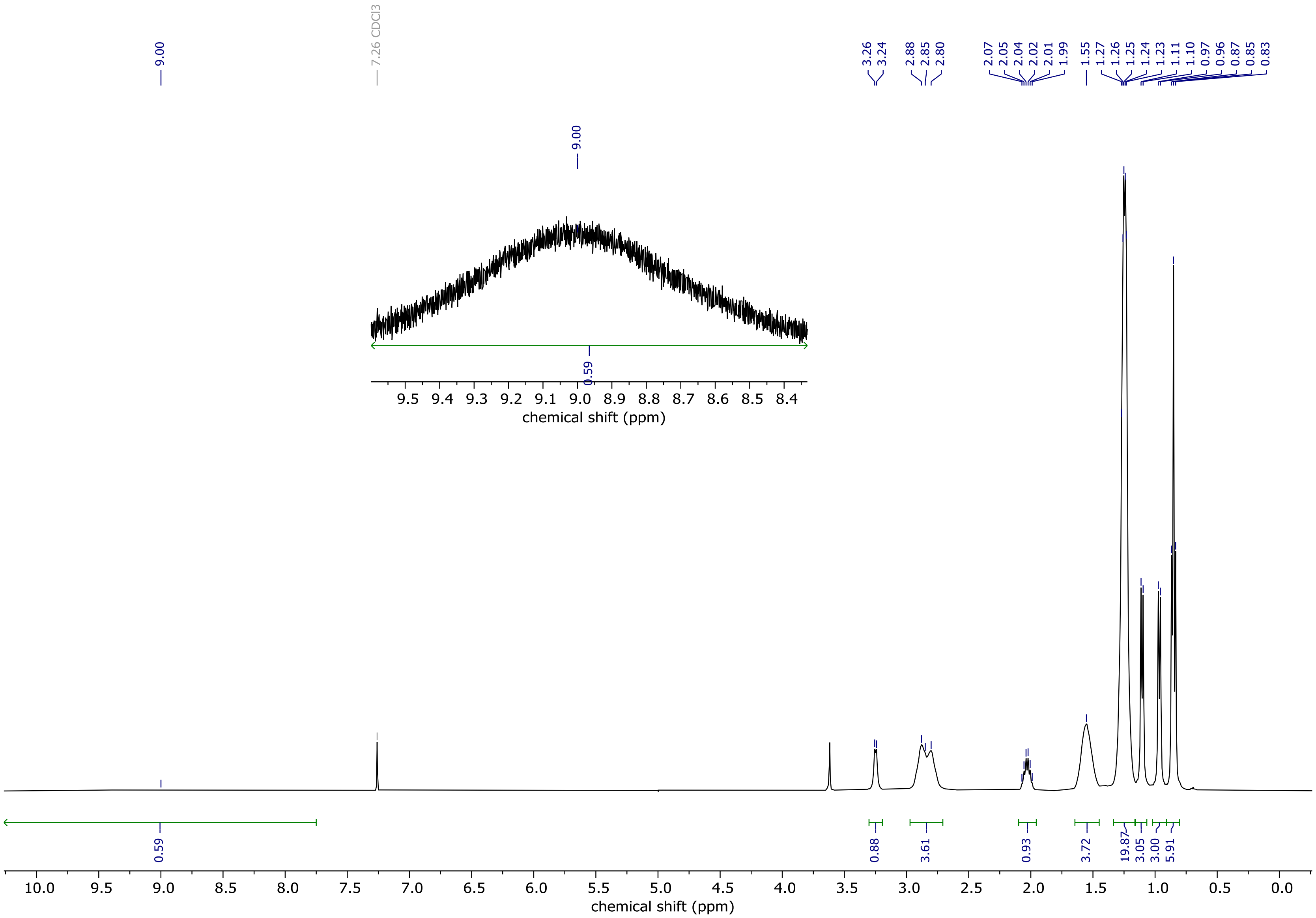

^{1}H-NMR of compound **ValC8** (400 MHz, $CDCl_3$, 25 °C).

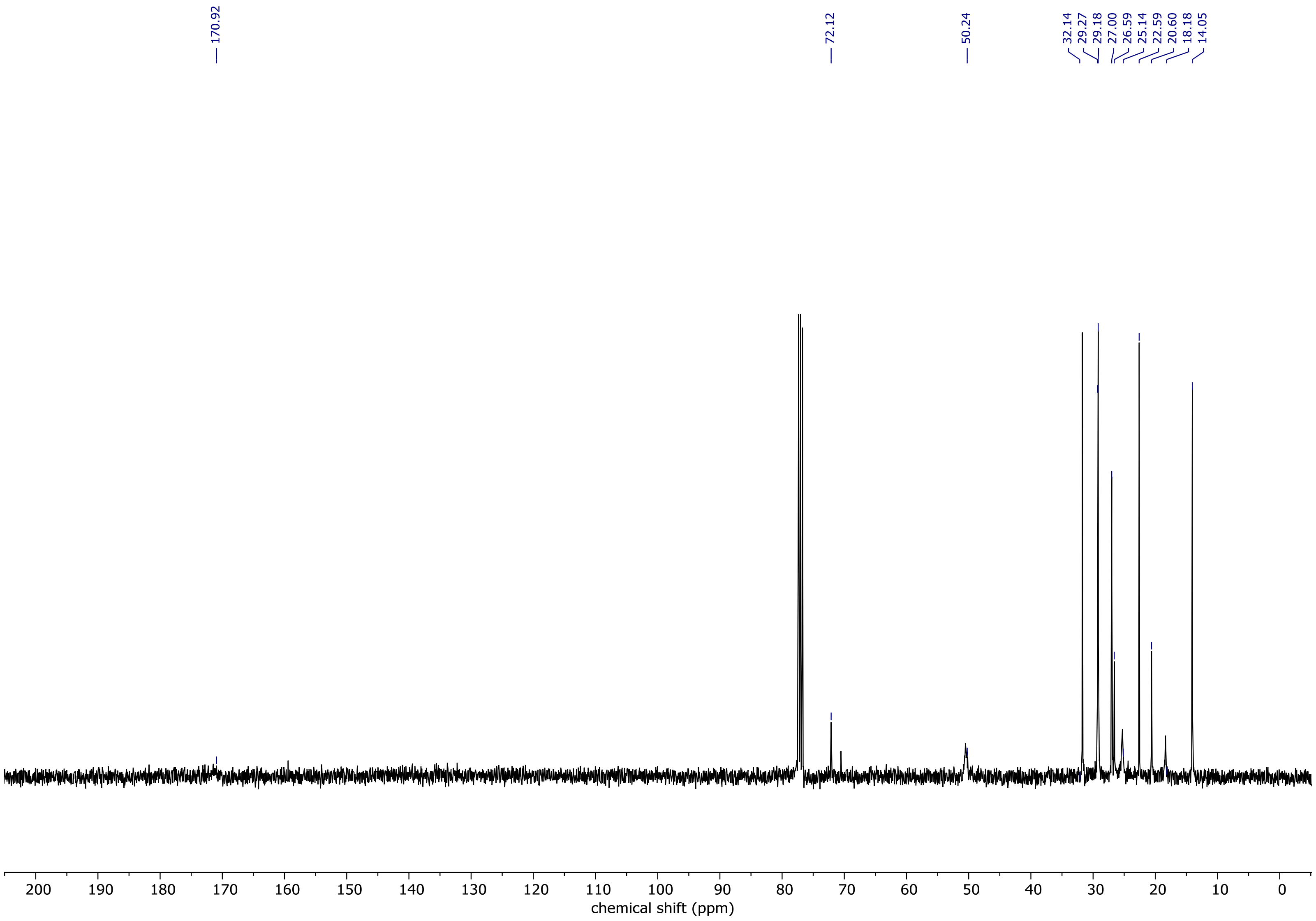

$^{13}C\{^{1}H\}$-NMR of compound **ValC8** (101 MHz, $CDCl_3$, 25 °C).

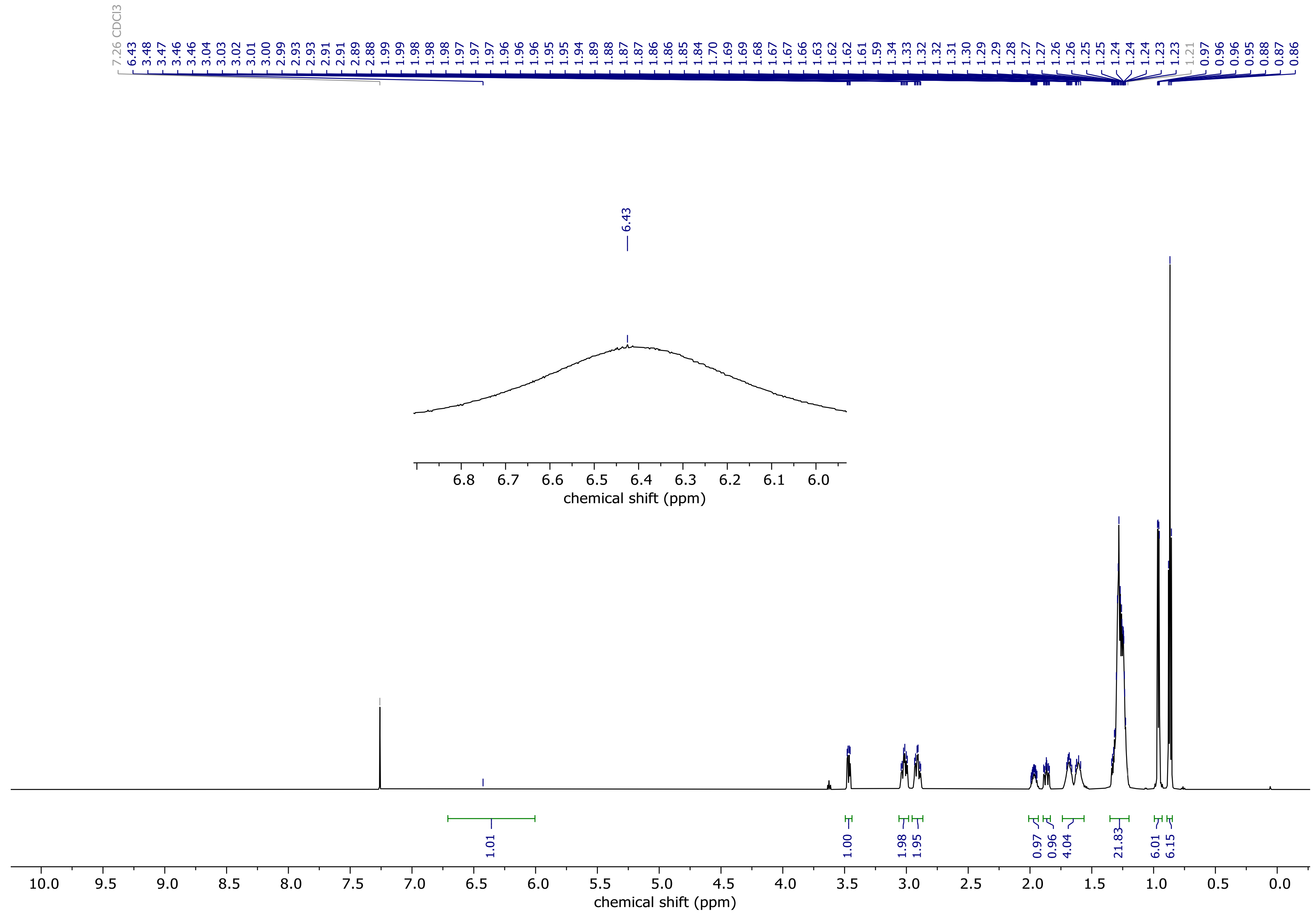

^{1}H-NMR of compound **LeuC8** (600 MHz, $CDCl_3$, 25 °C).

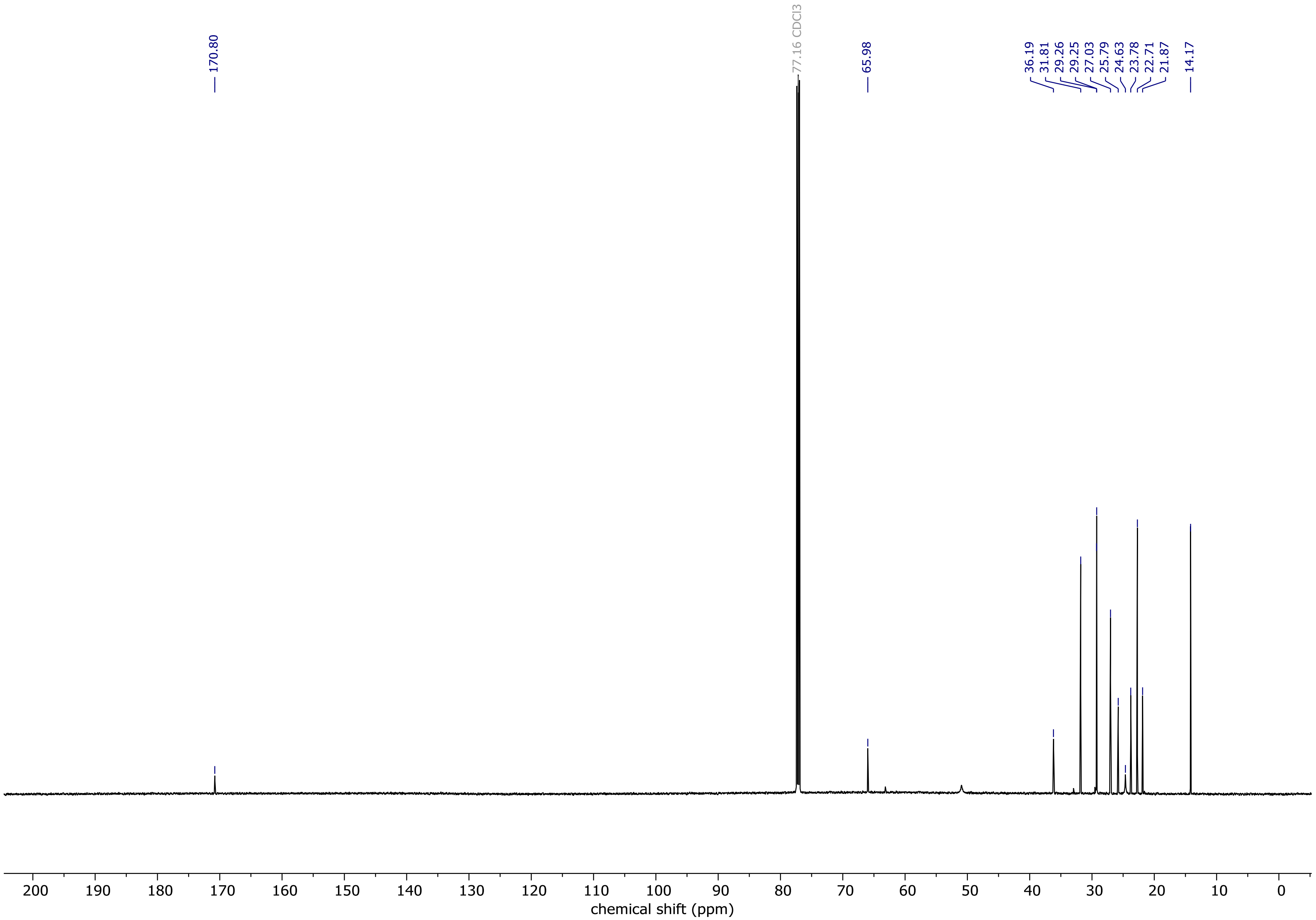

$^{13}C\{^{1}H\}$-NMR of compound **LeuC8** (151 MHz, $CDCl_3$, 25 °C).

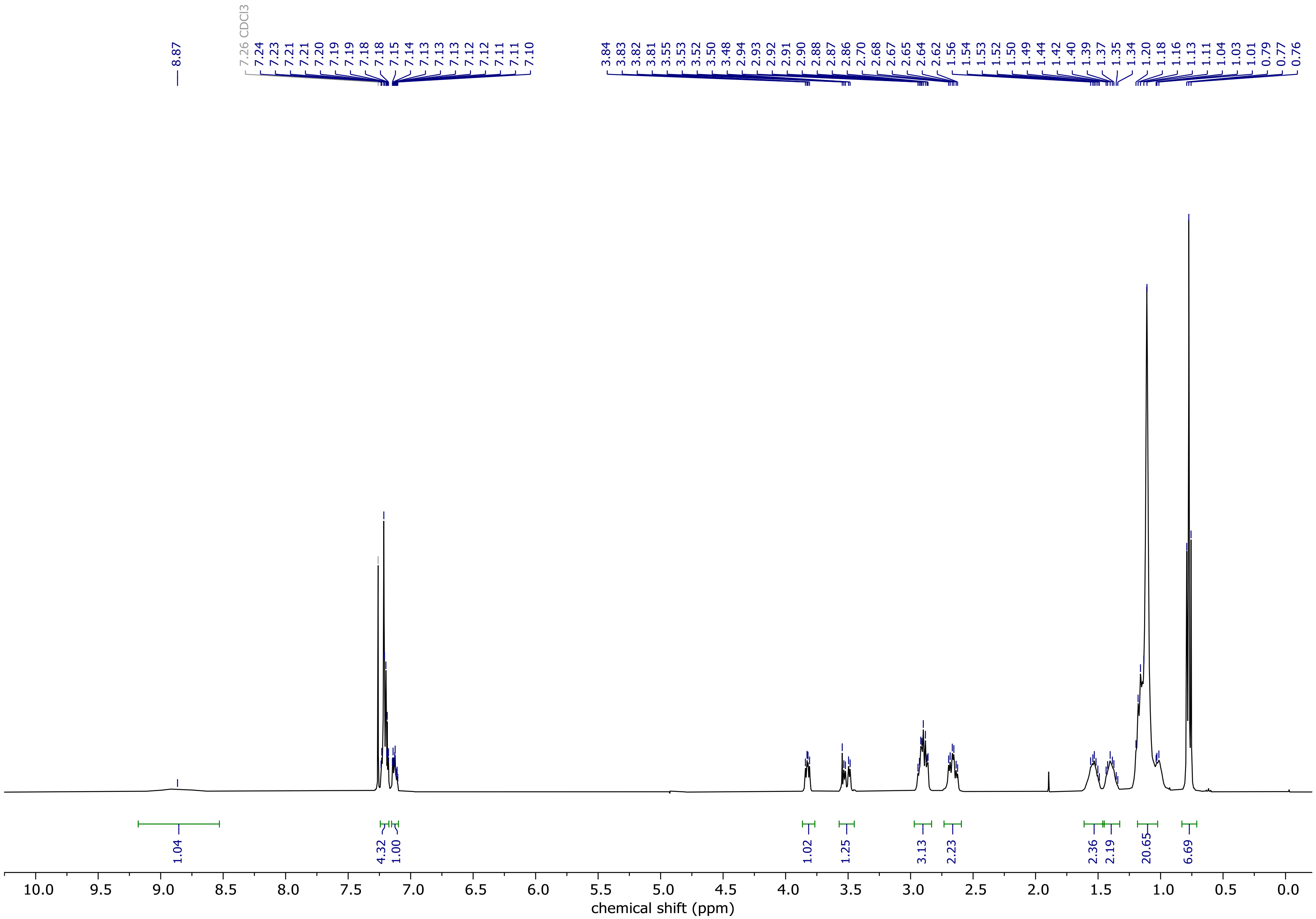

^{1}H-NMR of compound **PheC8** (400 MHz, $CDCl_3$, 25 °C).

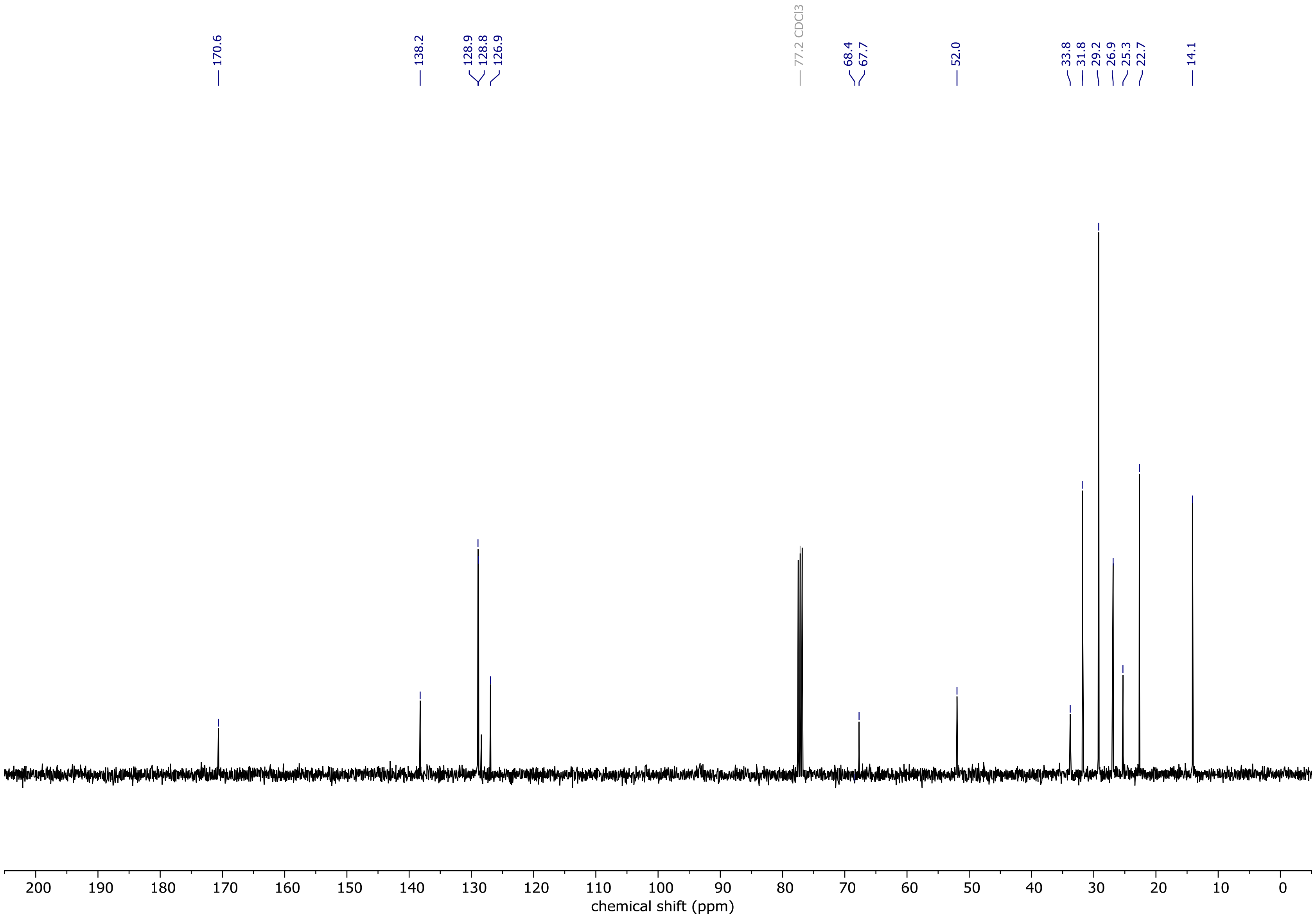

$^{13}C\{^{1}H\}$-NMR of compound **PheC8** (101 MHz, $CDCl_3$, 25 °C).

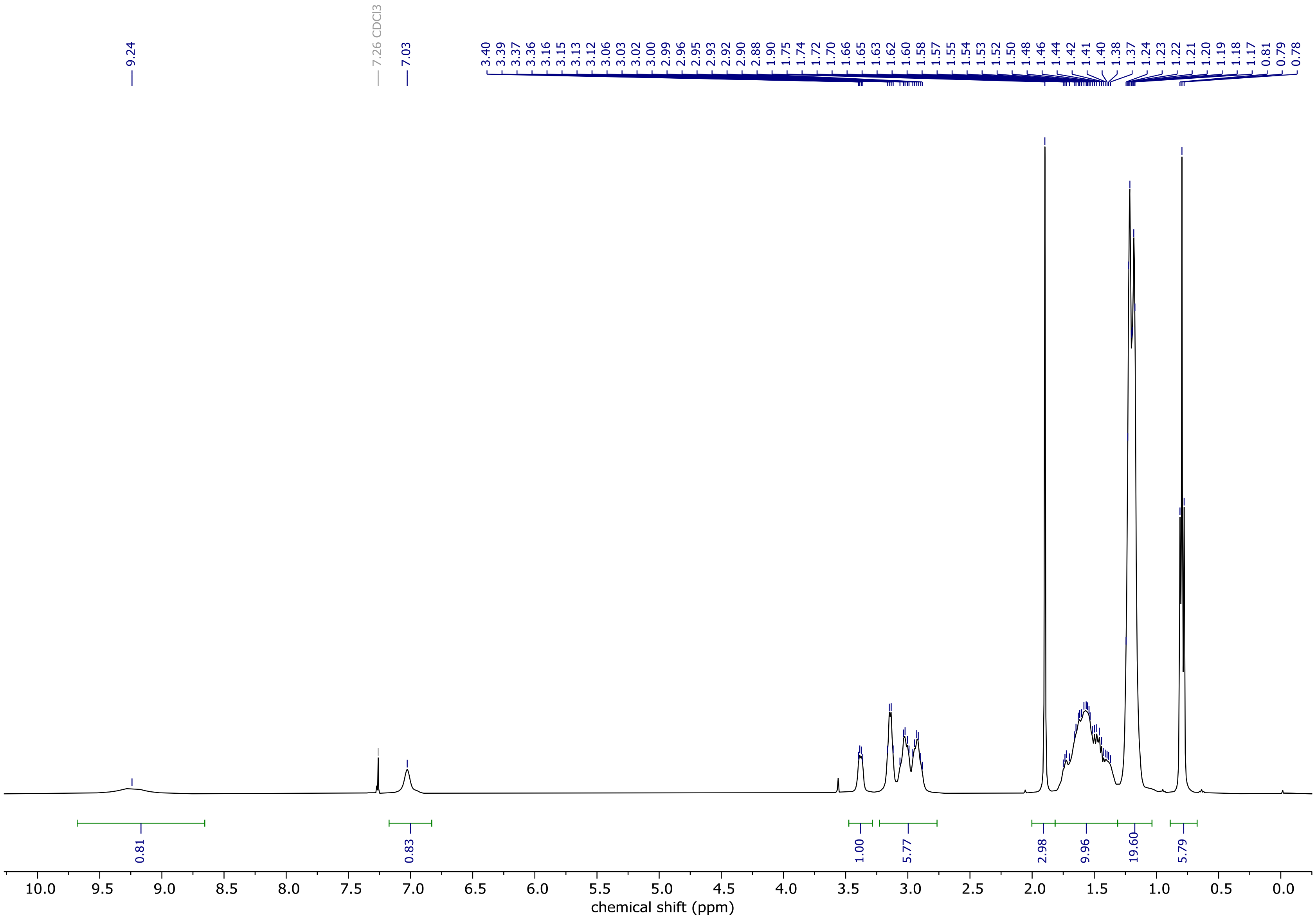

^{1}H-NMR of compound **LysC8** (400 MHz, $CDCl_3$, 25 °C).

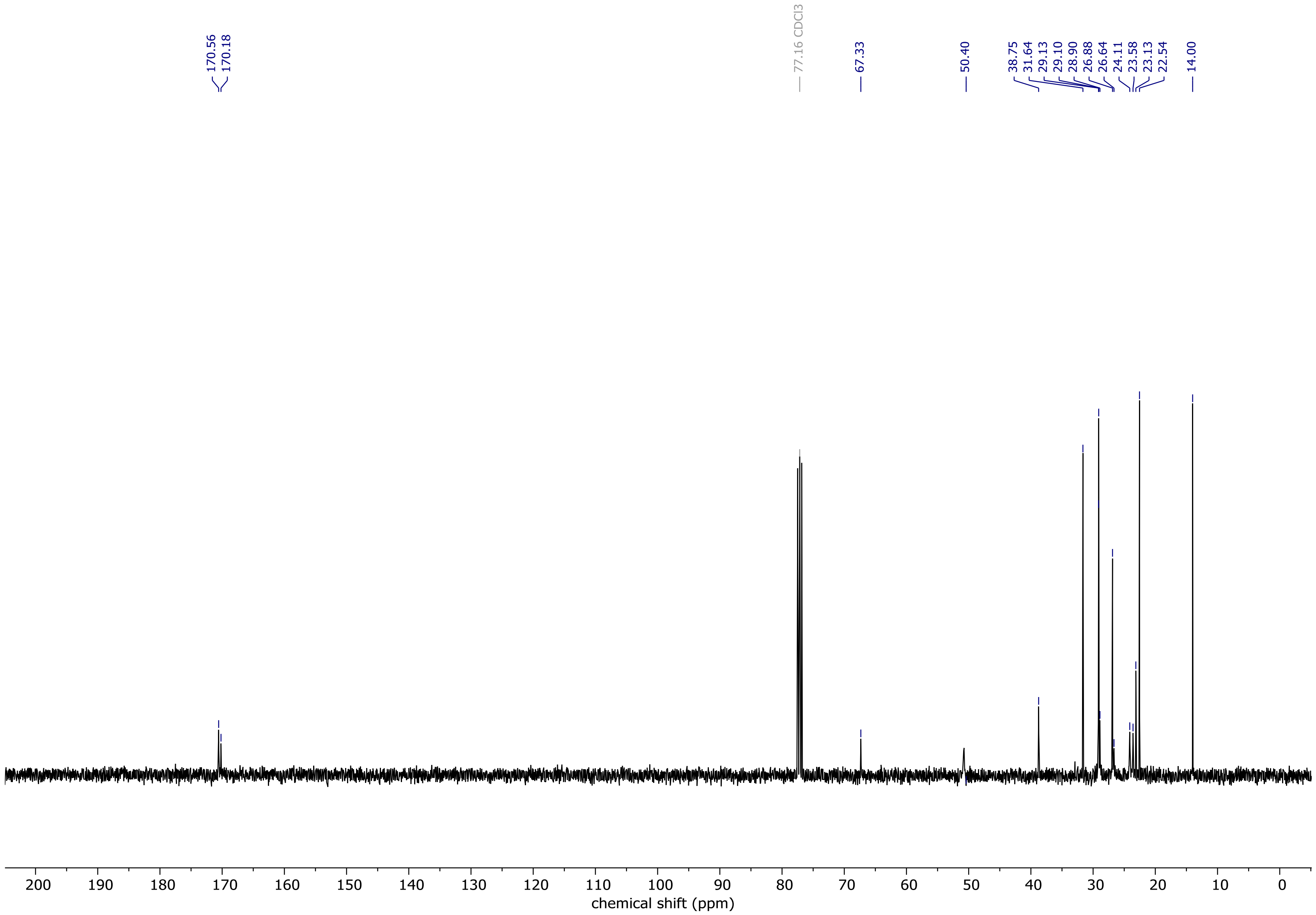

$^{13}C\{^1H\}$-NMR of compound **LysC8** (101 MHz, $CDCl_3$, 25 °C).

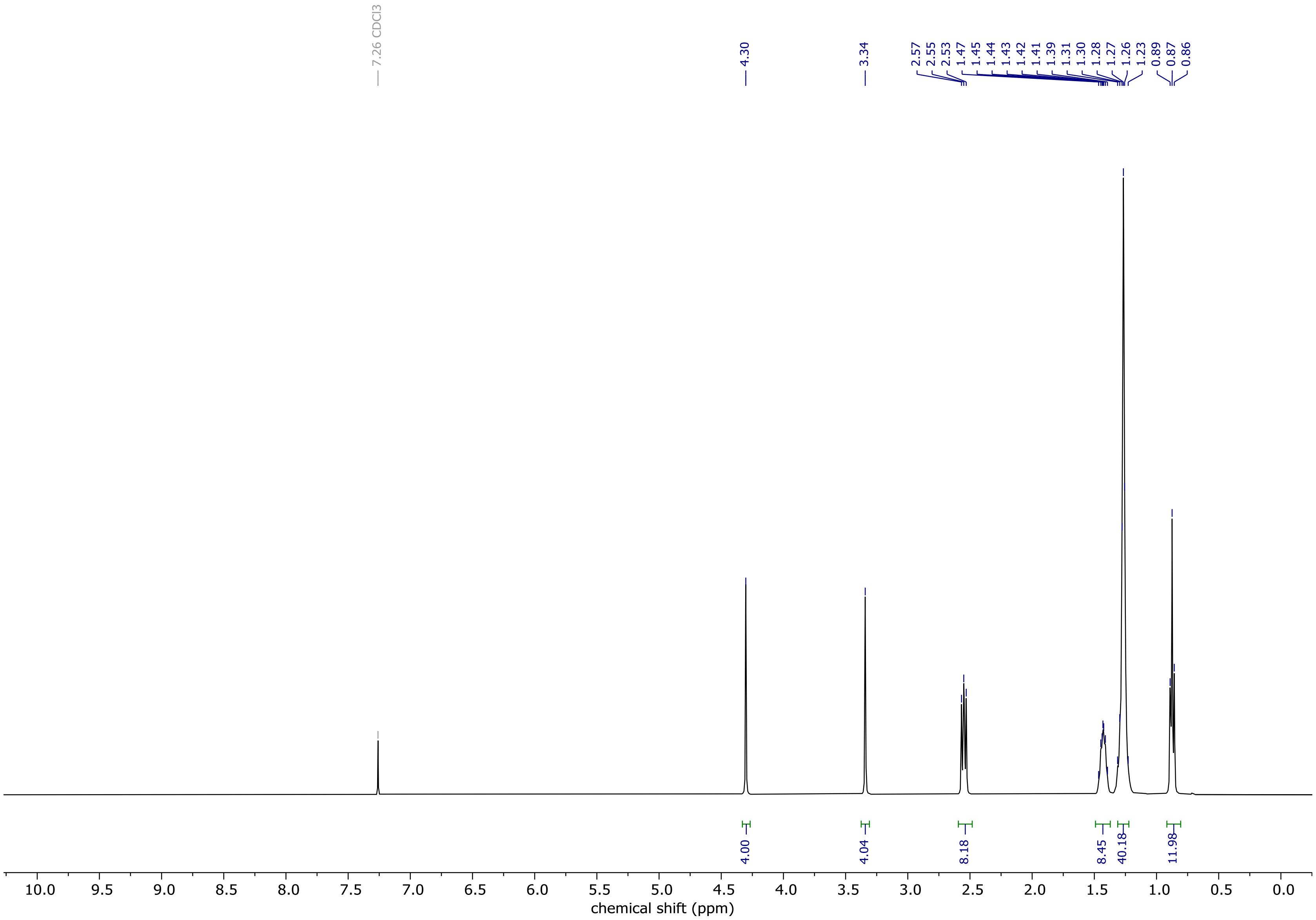

^{1}H-NMR of compound **GemC2** (400 MHz, $CDCl_3$, 25 °C).

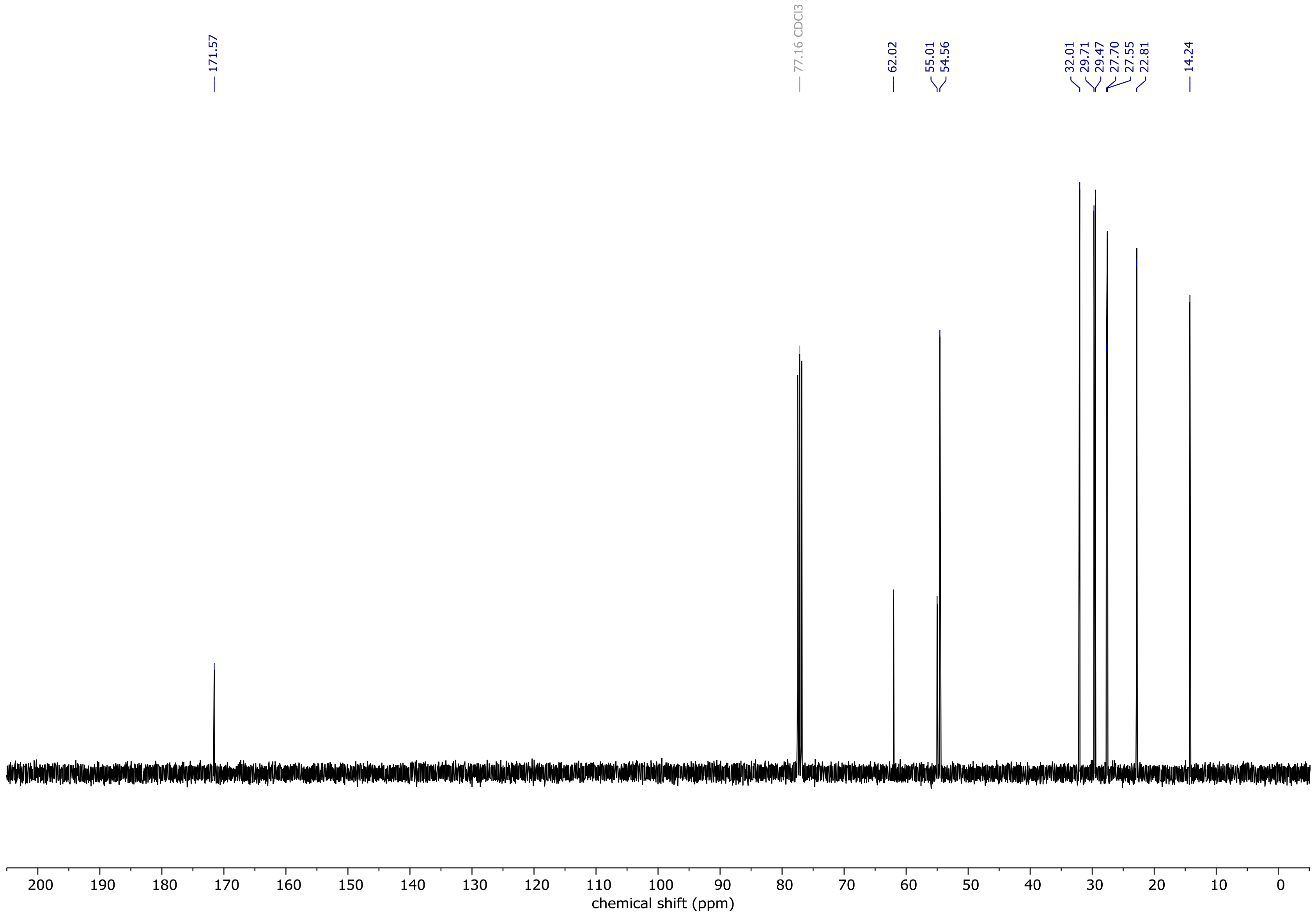

$^{13}C\{^{1}H\}$-NMR of compound **GemC2** (101 MHz, $CDCl_3$, 25 °C).

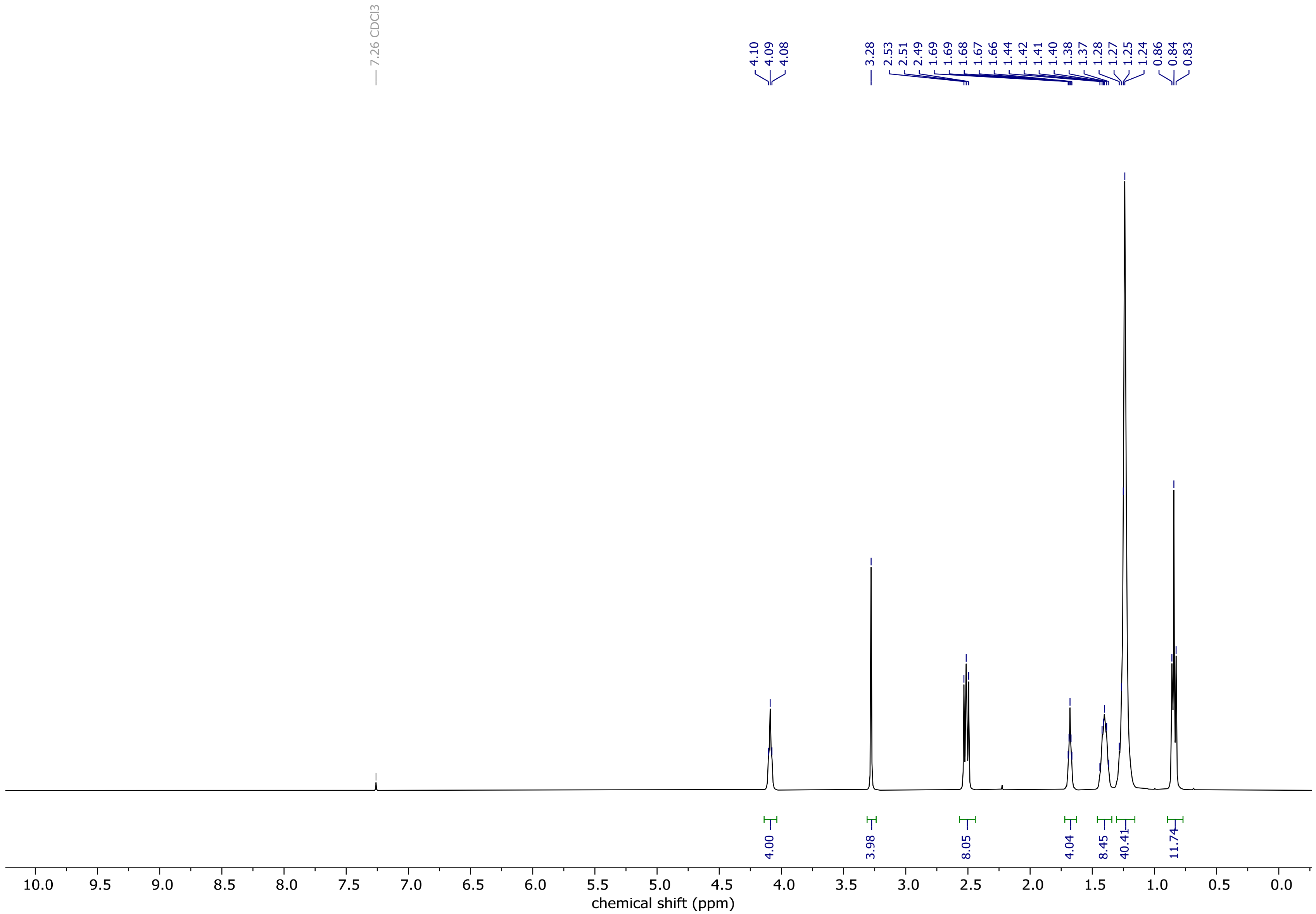

^{1}H-NMR of compound **GemC4** (400 MHz, $CDCl_3$, 25 °C).

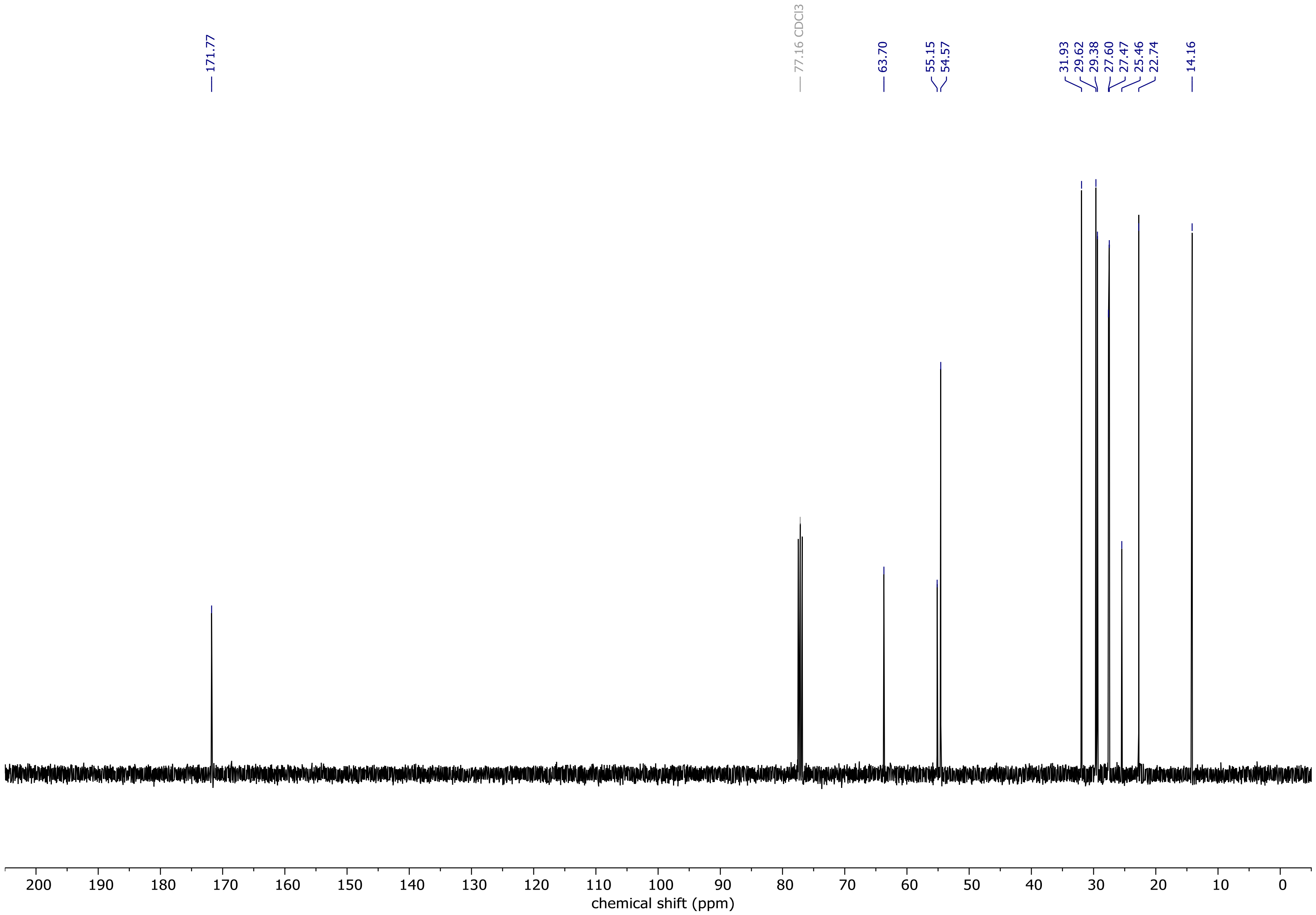

$^{13}C\{^1H\}$-NMR of compound **GemC4** (101 MHz, $CDCl_3$, 25 °C).

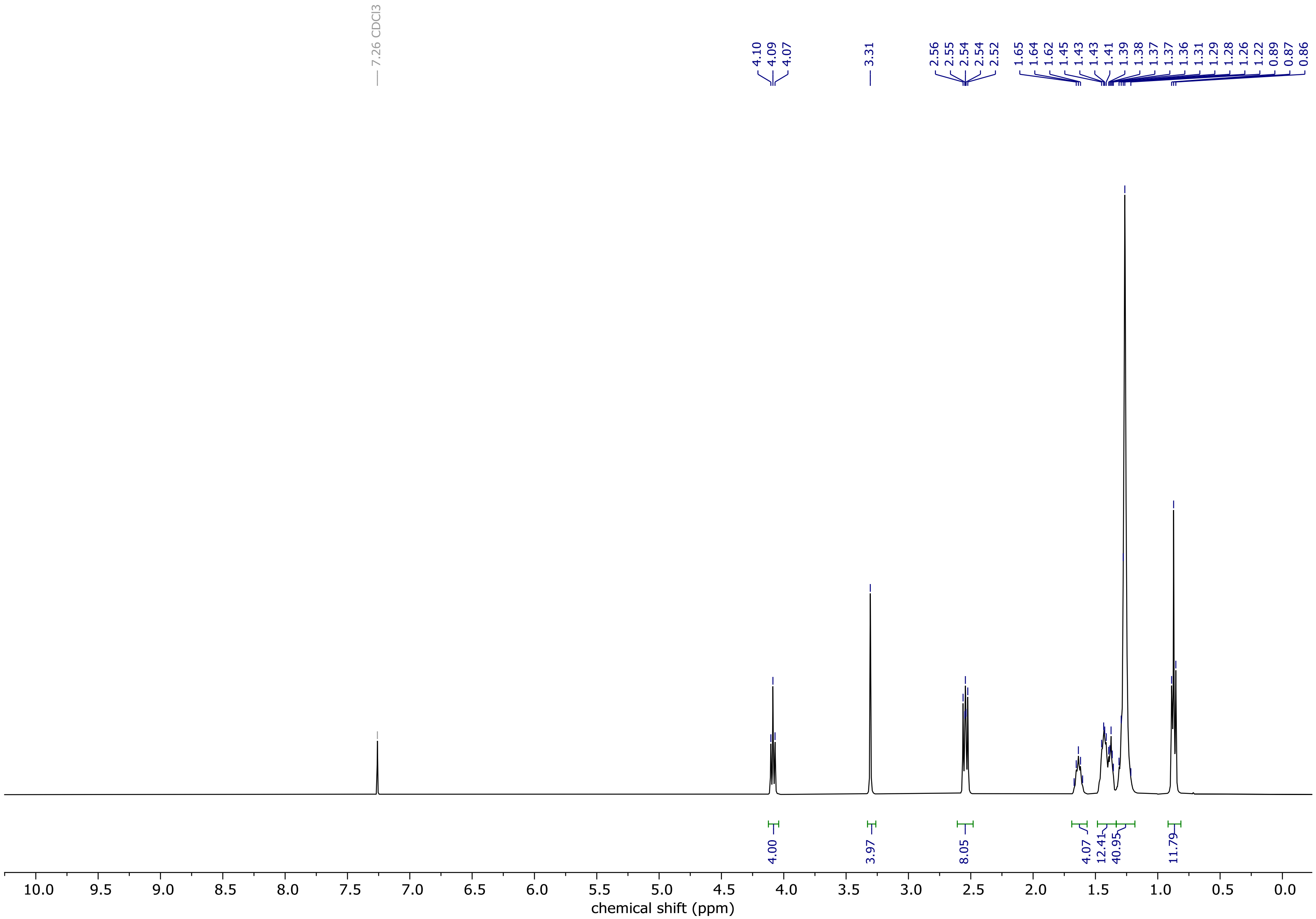

^{1}H-NMR of compound **GemC6** (400 MHz, $CDCl_3$, 25 °C).

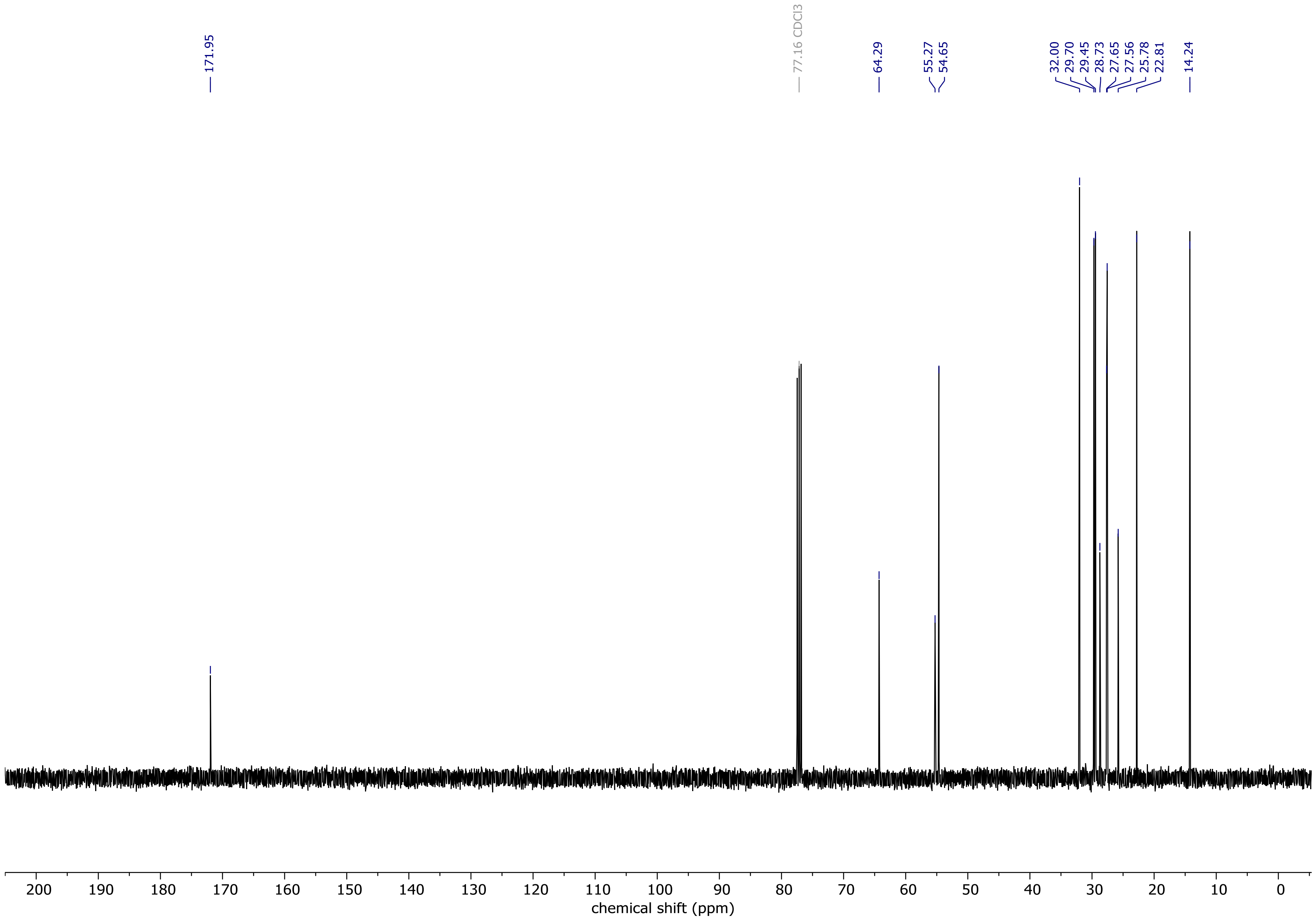

$^{13}C\{^{1}H\}$-NMR of compound **GemC6** (101 MHz, $CDCl_3$, 25 °C).

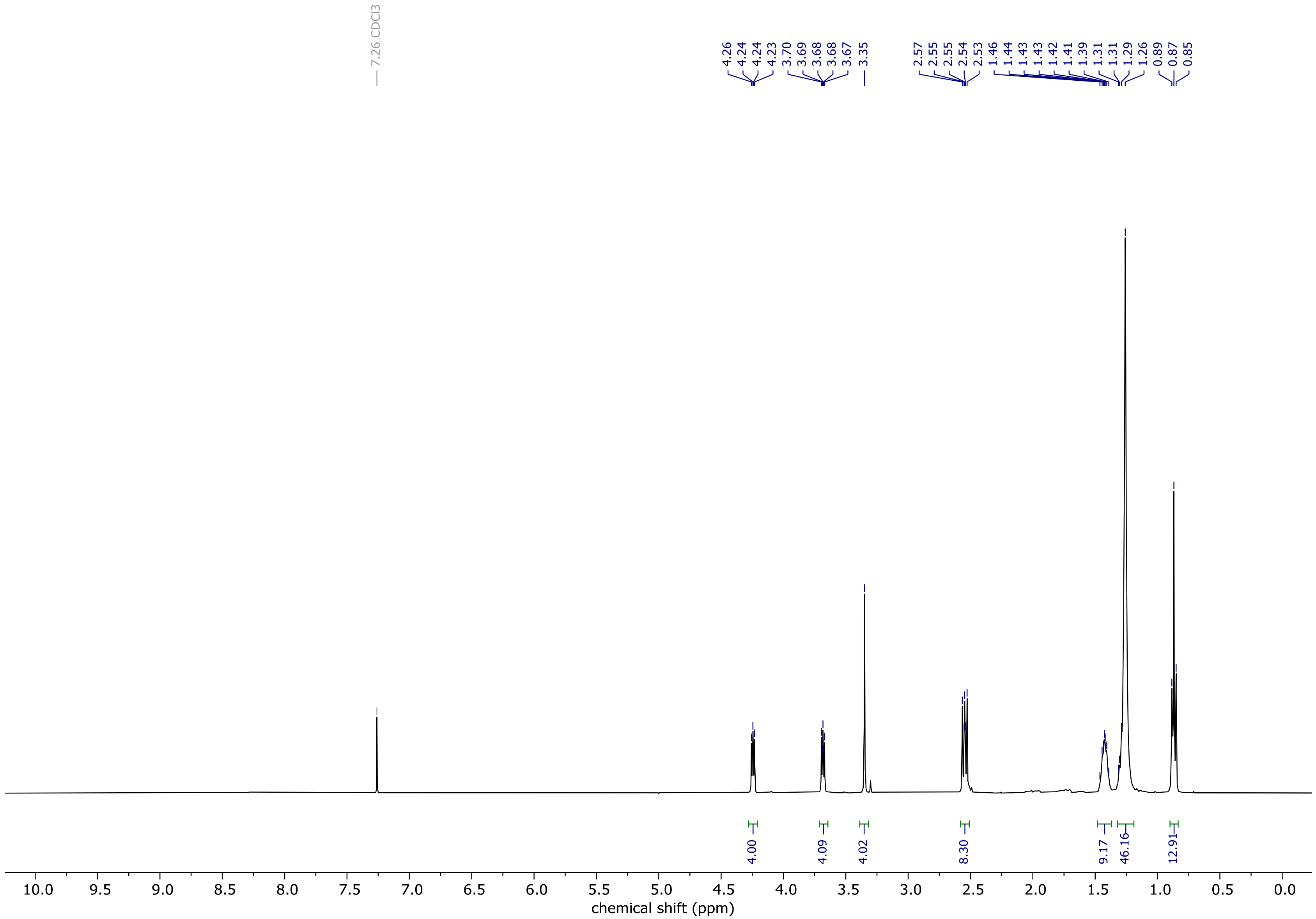

^{1}H-NMR of compound **GemGlyc** (400 MHz, $CDCl_3$, 25 °C).

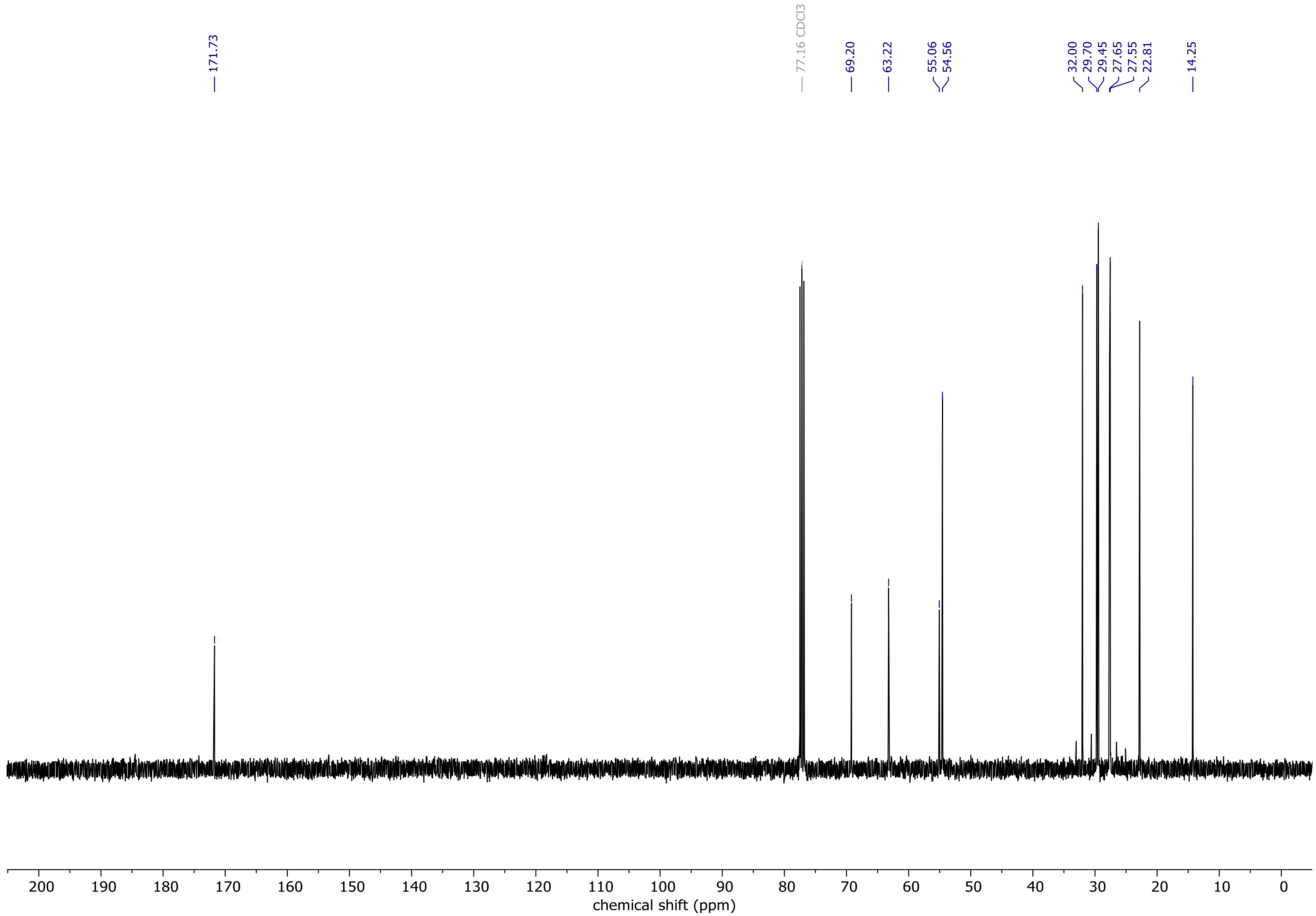

$^{13}C\{^{1}H\}$-NMR of compound **GemGlyc** (101 MHz, $CDCl_3$, 25 °C).

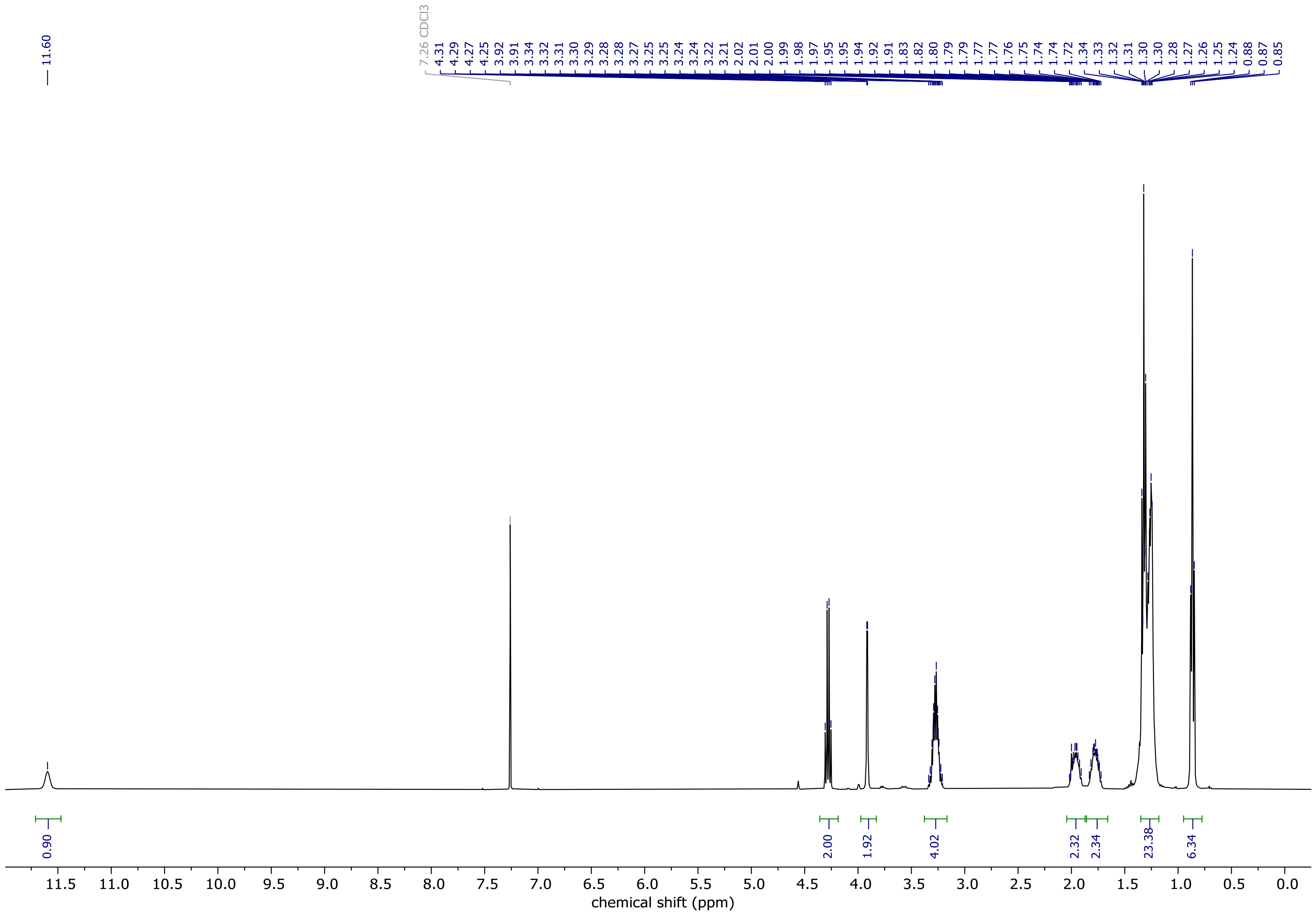

^{1}H-NMR of compound **GlyC8*EtBr** (400 MHz, $CDCl_3$, 25 °C).

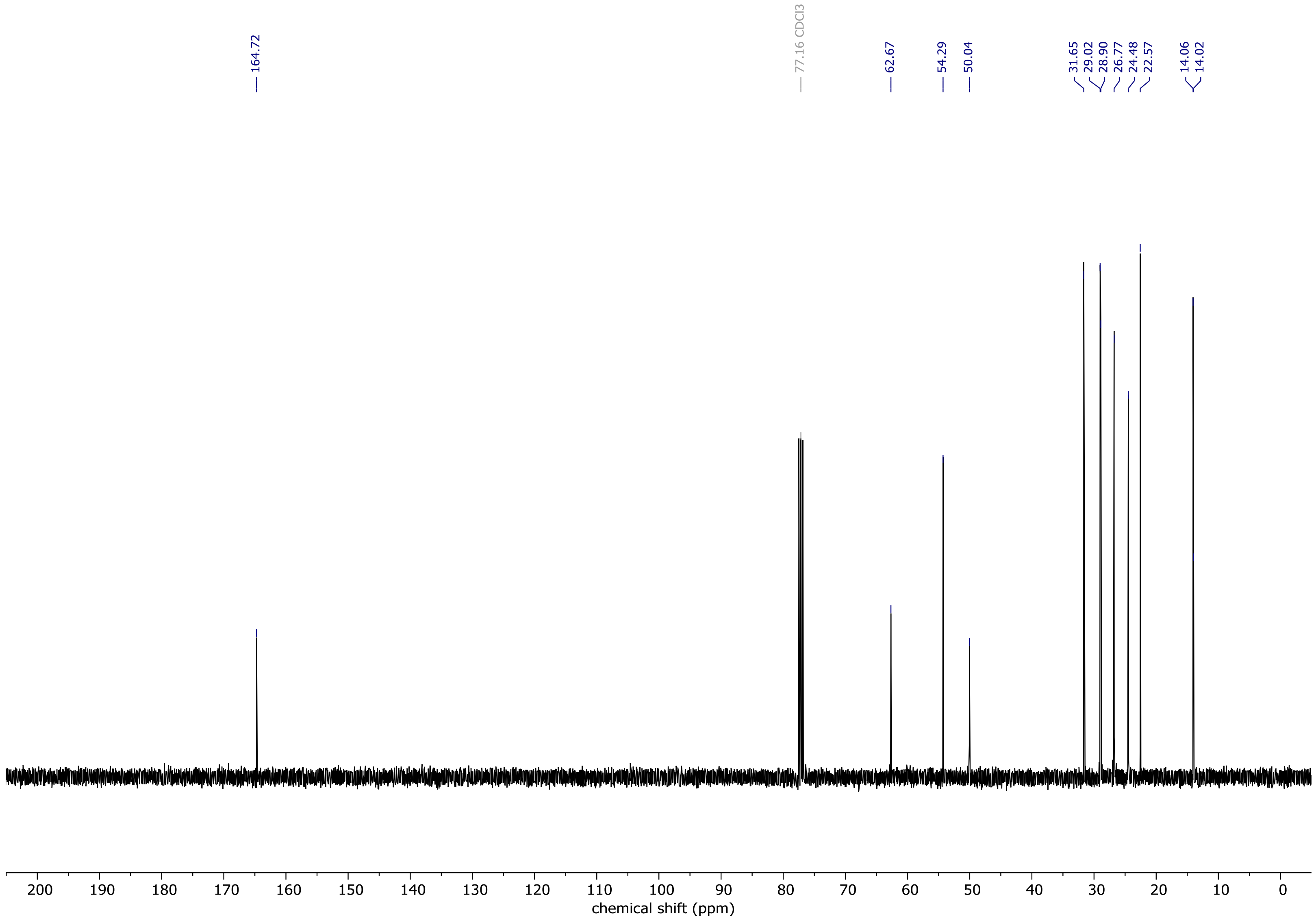

$^{13}C\{^{1}H\}$-NMR of compound **GlyC8*EtBr** (101 MHz, $CDCl_3$, 25 °C).

7. References

[42] OECD, *Test No. 301F: Ready Biodegradability: Manometric respirometry test* in *OECD Guidelines for the Testing of Chemicals, Section 3*, OECD, Paris, **1992**, pp. 48-55.
[43] B. Neises, W. Steglich, *Angew. Chem. Int. Ed. Engl.* **1978**, *17*, 522–524.
[44] OECD, *Revised Introduction to the OECD Guidelines for Testing of Chemicals, Section 3*, in *OECD Guidelines for the Testing of Chemicals, Section 3*, OECD, Paris, **2006**, pp. 1-17.
[45] M. J. Abraham, T. Murtola, R. Schulz, S. Páll, J. C. Smith, B. Hess, E. Lindahl, *SoftwareX* **2015**, *1–2*, 19–25.
[46] K. Vanommeslaeghe, E. Hatcher, C. Acharya, S. Kundu, S. Zhong, J. Shim, E. Darian, O. Guvench, P. Lopes, I. Vorobyov, A. D. Mackerell Jr., *J. Comput. Chem.* **2009**, *32*, 671–690.
[47] W. L. Jorgensen, J. Chandrasekhar, J. D. Madura, R. W. Impey, M. L. Klein, *J. Chem. Phys.* **1983**, *79*, 926–935.
[48] Gaussian 09, Revision A.02, M. J. Frisch, G. W. Trucks, H. B. Schlegel, G. E. Scuseria, M. A. Robb, J. R. Cheeseman, G. Scalmani, V. Barone, G. A. Petersson, H. Nakatsuji, X. Li, M. Caricato, A. Marenich, J. Bloino, B. G. Janesko, R. Gomperts, B. Mennucci, H. P. Hratchian, J. V. Ortiz, A. F. Izmaylov, J. L. Sonnenberg, D. Williams-Young, F. Ding, F. Lipparini, F. Egidi, J. Goings, B. Peng, A. Petrone, T. Henderson, D. Ranasinghe, V. G. Zakrzewski, J. Gao, N. Rega, G. Zheng, W. Liang, M. Hada, M. Ehara, K. Toyota, R. Fukuda, J. Hasegawa, M. Ishida, T. Nakajima, Y. Honda, O. Kitao, H. Nakai, T. Vreven, K. Throssell, J. A. Montgomery, Jr., J. E. Peralta, F. Ogliaro, M. Bearpark, J. J. Heyd, E. Brothers, K. N. Kudin, V. N. Staroverov, T. Keith, R. Kobayashi, J. Normand, K. Raghavachari, A. Rendell, J. C. Burant, S. S. Iyengar, J. Tomasi, M. Cossi, J. M. Millam, M. Klene, C. Adamo, R. Cammi, J. W. Ochterski, R. L. Martin, K. Morokuma, O. Farkas, J. B. Foresman, and D. J. Fox, Gaussian, Inc., Wallingford CT, **2016**.
[49] D. M. Chipman, *J. Chem. Phys.* **2000**, *112*, 5558–5565.
[50] U. Essmann, L. Perera, M. L. Berkowitz, T. Darden, H. Lee, L. G. Pedersen, *J. Chem. Phys.* **1995**, *103*, 8577–8593.
[51] B. Hess, H. Bekker, H. J. C. Berendsen, J. G. E. M. Fraaije, *J. Comp. Chem.* **1997**, *18*, 1463–1472.
[52] C. H. Bennett, *J. Comput. Phys.* **1976**, *22*, 245–268.
